# Rhodium-Catalyzed C(sp^2^)–H Alkoxycarbonylation/Acylation of Indolines with Anhydrides as a Carbonyl Source

**DOI:** 10.1021/acs.orglett.1c04195

**Published:** 2022-01-31

**Authors:** Hirotsugu Suzuki, Fumito Sasamori, Takanori Matsuda

**Affiliations:** Department of Applied Chemistry, Tokyo University of Science, 1-3 Kagurazaka, Shinjuku-ku, Tokyo 162-8601, Japan

## Abstract

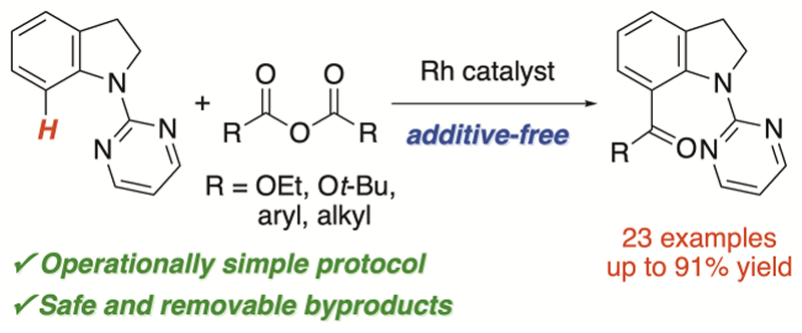

We developed rhodium-catalyzed alkoxylcarbonylation/acylation of indolines using anhydrides as a safe and easy-to-handle carbonyl source. This catalytic process represents an additive- and CO-free carbonylation, establishing a simple and straightforward protocol for synthesizing C7-carbonylated indolines. Notably, this reaction provides a successful example of C–H acylation of indolines that results in the formation of α-branched ketones, which were difficult to prepare by previously reported analogous catalytic reactions.

C7-Carbonylated indoles and their derivatives are an important class of biologically active compounds found in many natural products, pharmaceuticals, and agrochemicals (Scheme 1).^1^ Common reactions to access C7-carbonylated indoles are palladium-catalyzed carbonylation of 7-haloindoles using carbon monoxide (CO),^2^ Stille coupling using (1-ethoxyvinyl)stannane,^3^ and nucleophilic addition of C7-metalated indoles to carbonyl donors.^4^ Although these well-established protocols provide a reliable route to C7-carbonylated indoles, the prefunctionalization of starting materials makes the reaction less attractive. Moreover, these reactions usually require air- and moisture-sensitive organometallics and harmful reagents. Thus, the development of a simple and efficient procedure to access C7-carbonylated indoles is highly desirable.^5^

**Scheme 1:**
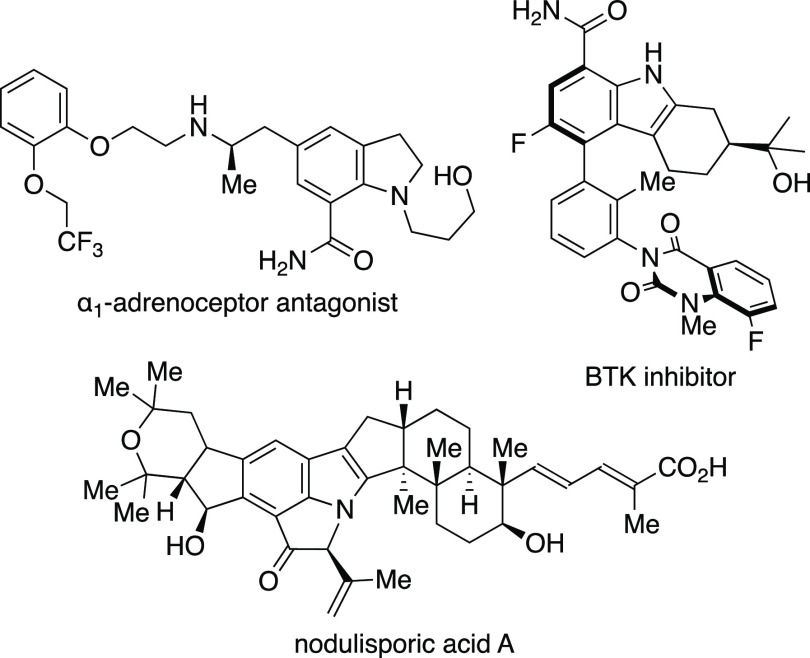
Selected Examples of Biologically Active C7-Carbonylated Indoles and Their Derivatives

C(sp^2^)–H functionalization of indolines is one of the most straightforward synthetic pathways for C7-functionalized indoles,^6,7^ leading to the investigation of a variety of organic transformations, including carbonylation reactions. In 2002, Chatani et al. reported ruthenium-catalyzed carbonylation of indolines with CO and alkenes.^8^ Subsequently, the oxidative amino- and alkoxycarbonylation of indolines under a CO atmosphere has been reported by other groups.^9^ In contrast, C(sp^2^)–H carbonylation of indolines using various carbonyl sources such as azodicarboxylates,^10^ isocyanates,^11^ α-keto acids,^12^ aldehydes,^13^ 1,2-diketones,^14^ and glyoxalates^15^ has been studied under CO-free conditions (Scheme 2a). Although these carbonyl sources are less toxic and easy to handle, the use of an external additive such as an oxidant and a base limits their application by producing a stoichiometric amount of unwanted byproducts. Consequently, this complicates the experimental procedure and narrows the substrate scope under oxidative or basic reaction conditions. Despite these critical problems, the additive-free C(sp^2^)–H carbonylation of indolines is yet to be addressed. We hypothesized that the use of dicarbonates and carboxylic acid anhydrides as a carbonyl source may provide a solution for the additive-free C–H carbonylation of indolines.^16,17^ Herein, we describe the additive-free C7-selective carbonylation of indolines using dialkyl dicarbonates and carboxylic acid anhydrides as a safe and easy-to-handle carbonyl source (Scheme 2b).

**Scheme 2:**
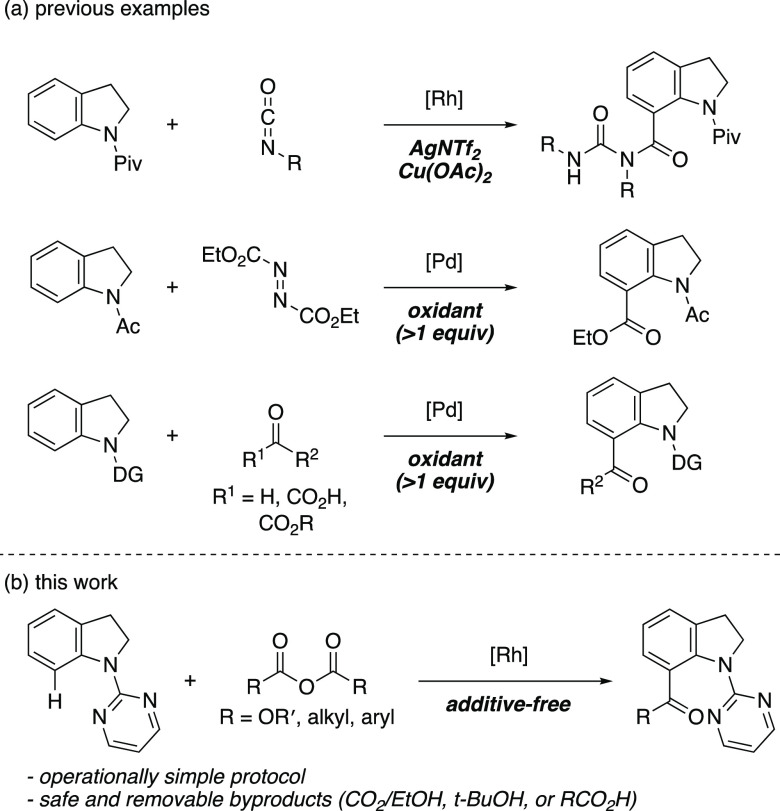
C7-Carbonylation of Indolines under CO-Free Conditions

First, the ethoxycarbonylation of 1-(pyrimidin-2-yl)indoline (**1a**) as a model substrate was investigated (Table 1). An initial reaction was performed using diethyl dicarbonate (**2a**) as an ethoxycarbonyl source in the presence of [Rh(cod)_2_]OTf. The reaction was conducted at 100 °C for 18 h and yielded the desired indoline-7-carboxylic acid ester **3aa** (entry 1). Based on these results, other rhodium catalysts such as [RhCl(cod)]_2_, RhCl(PPh_3_)_3_, [RhCl(CO)_2_]_2_, Rh(acac)(CO)_2_, [Cp*RhCl_2_]_2_, and [Cp*Rh(MeCN)_3_](SbF_6_)_2_ were tested; [RhCl(CO)_2_]_2_ proved to be the optimal catalyst for this process (entries 2–7). Acetonitrile gave the best results among the solvents examined (entries 8–12). A control experiment revealed that the rhodium catalyst was essential for this reaction (entry 13).

**Table 1:**
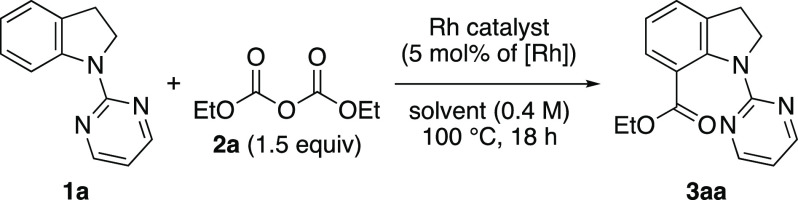
Optimization of Reaction Conditions^a^

entry	Rh catalyst	solvent	yield^b^ (%)
1	[Rh(cod)_2_]OTf	1,4-dioxane	25
2	[RhCl(cod)]_2_	1,4-dioxane	0
3	RhCl(PPh_3_)_3_	1,4-dioxane	0
4	[RhCl(CO)_2_]_2_	1,4-dioxane	86
5	Rh(acac)(CO)_2_	1,4-dioxane	8
6	[Cp*RhCl_2_]_2_	1,4-dioxane	0
7	[Cp*Rh(MeCN)_3_](SbF_6_)_2_	1,4-dioxane	0
8	[RhCl(CO)_2_]_2_	THF	83
9	[RhCl(CO)_2_]_2_	toluene	79
10	[RhCl(CO)_2_]_2_	DCE	91
11	[RhCl(CO)_2_]_2_	DMF	11
12	[RhCl(CO)_2_]_2_	MeCN	99 (88)
13	–	MeCN	0

a Standard conditions: **1a** (0.2 mmol), **2a** (0.3 mmol), and Rh catalyst (5 mol % of [Rh]) in the solvent (0.5 mL) at 100 °C for 18 h.

b Yields were determined by ^1^H NMR analysis using 1,2,4,5-tetramethylbenzene as an internal standard. Value in parentheses indicates isolated yield, which represents the average of two runs.

With the optimized reaction conditions in hand, the additive-free alkoxycarbonylation using various indoline derivatives was investigated (Table 2). Indolines bearing a methyl and a phenyl group at the 2- or 3-position resulted in the formation of the desired indoline-7-carboxylic acid esters in good to high yields (**3ba**–**da**). Introducing a methyl group at the 4-position did not influence the reactivity (**3ea**). Indolines bearing electron-donating and electron-withdrawing substituents at the C5 position delivered the desired products in 86–91% yields (**3fa**–**ia**). Although a 6-fluoro indoline produced the desired product in high yield (**3ja**), the reaction of a 6-methyl indoline was sluggish presumably due to steric hindrance (**3ka**). A carbazole transformed into monoester **3la** in moderate yield (36%) along with a small amount (10%) of the double alkoxycarbonylation product. Gratifyingly, **1a** was coupled with di-*tert*-butyl dicarbonate to provide a good yield of **3ab**.

**Table 2:**
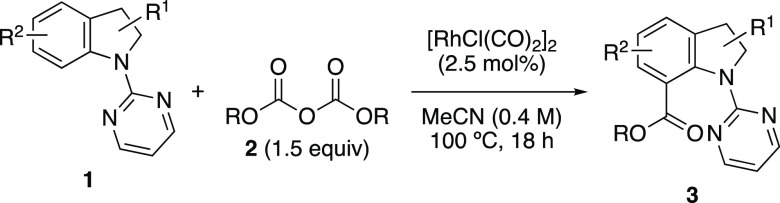

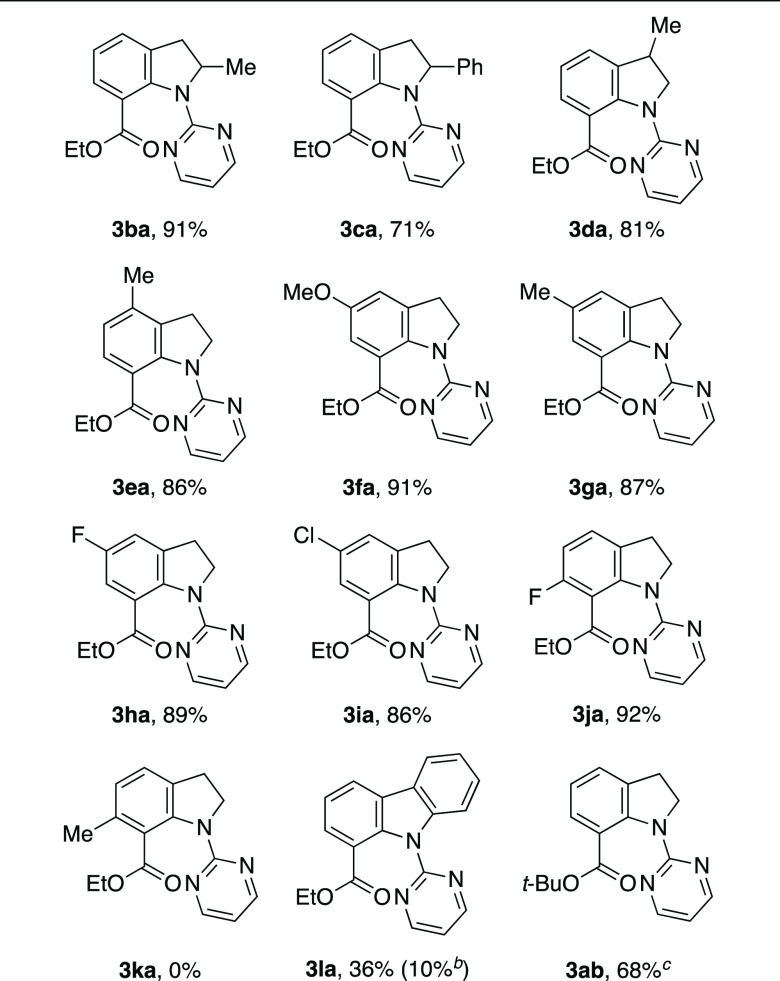
Substrate Scope of Indolines^a^

a Reaction conditions: **1** (0.2 mmol), **2** (0.3 mmol), and [RhCl(CO)_2_]_2_ (2.5 mol %) in MeCN (0.5 mL) at 100 °C for 18 h. Isolated yields represent the average of two runs.

b Yield of the double alkoxycarbonylation product.

c 2.0 equiv of Boc_2_O was used.

To demonstrate the efficacy of this transformation, the reaction of **1a** with **2a** in 1,4-dioxane^18^ was performed on a 5 mmol scale (Scheme 3). The reaction proceeded smoothly to furnish the product **3aa** in good yield. The pyrimidyl directing group in the product could be removed in two steps.^9b^

**Scheme 3:**
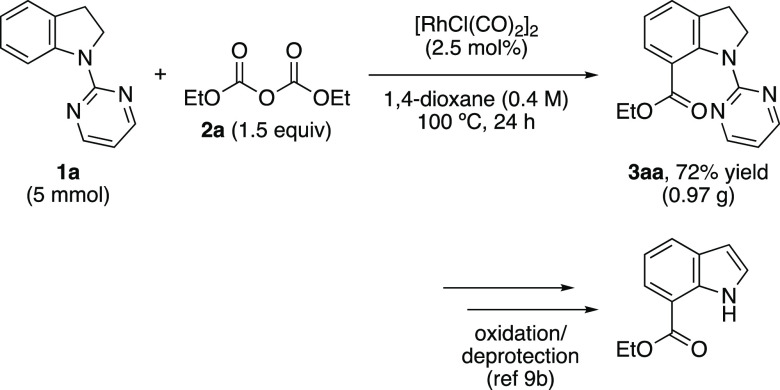
Large-Scale Reaction

A series of control experiments were performed to elucidate the reaction mechanism (Scheme 4). First, H/D exchange experiments were conducted by subjecting **1a** with D_2_O (5.0 equiv) to the standard reaction conditions in the presence or absence of diethyl dicarbonate (**2a**). A significant H/D scrambling was observed at the C7 position in both cases, which supports the reversibility of the C–H activation step. Furthermore, the kinetic isotope effect experiment (*k*_H_/*k*_D_ = 1.0) revealed that the C–H bond cleavage might not be involved in the rate-determining step.

**Scheme 4:**
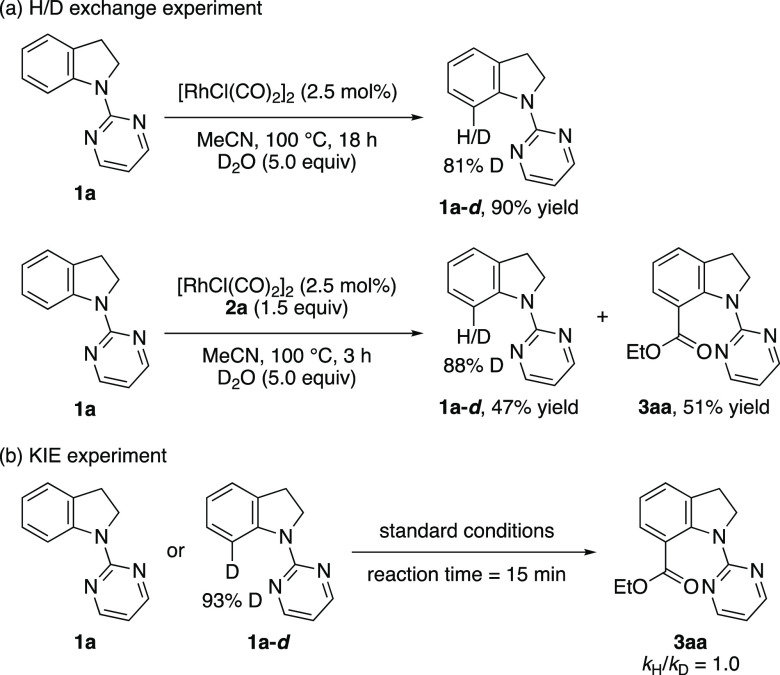
Control Experiments

Based on the experimental results and previous reports in the literature,^16,19^ the reaction mechanism for the additive-free alkoxycarbonylation is proposed (Figure 1). The C–O bond of dicarbonate **2** undergoes oxidative addition to rhodium(I) **A** to form a rhodium(III) intermediate **B** along with the extrusion of CO_2_. The C7-selective C–H activation of indoline **1** by intermediate **B** provides six-membered rhodacycle **C**, which subsequently transforms into the desired product **3** via reductive elimination, and the active catalyst **A** is regenerated. However, another reaction mechanism involving initial oxidative addition of **1a** to **A** to form a rhodium(III) intermediate cannot be ruled out at this stage.^5,19b^

**Figure 1:**
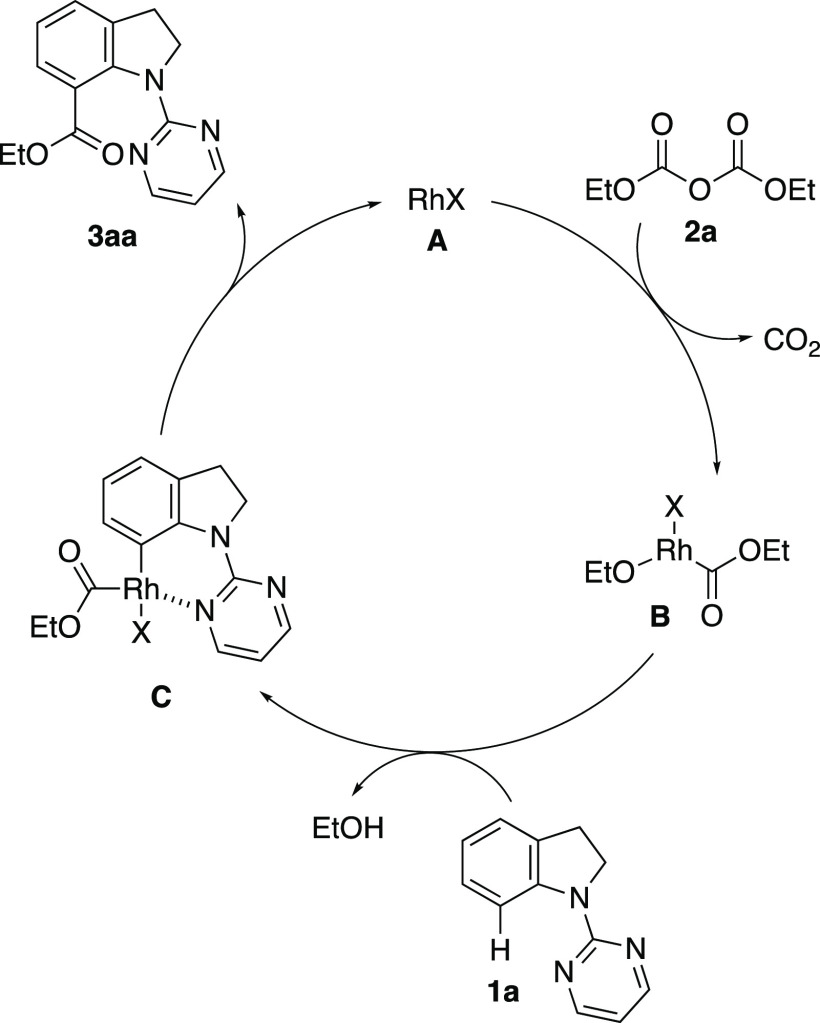
Proposed reaction mechanism for the rhodium-catalyzed additive-free alkoxycarbonylation of **1a** with **2a**.

Next, the C7-selective acylation of indolines using symmetrical carboxylic acid anhydrides was investigated. Although carboxylic acid anhydrides are known to be good acyl sources in the C3-selective Friedel–Crafts acylation of indoles,^20^ the corresponding C7-acylation has scarcely been reported.^5^ This is due to the decarbonylation process that occurs at high temperatures (>130 °C).^19^ It was assumed that the C7-acylation of indolines using a carboxylic acid anhydride might proceed without decarbonylation if the optimized reaction conditions were applied. The reaction of indoline **1a** with acetic anhydride was initially examined under the optimal conditions for the alkoxycarbonylation. Unfortunately, the desired acylated indoline **5aa** was obtained in moderate yield along with a small amount of the methylation product, 7-methyl-1-(pyrimidin-2-yl)indoline, which formed via decarbonylation. Thus, the reaction conditions were slightly modified, and the optimal conditions for the acylation were identified as follows: 5 mol % of [Rh(CO)_2_Cl]_2_ in DMF at 80 °C for 24 h.^21^

Subsequently, the scope of acylation with various symmetrical carboxylic acid anhydrides was investigated (Table 3). Indoline **1a** was coupled with acetic anhydride and propionic anhydride to form the corresponding 7-acylated indolines **5aa** and **5ab**, respectively, in good yields. Notably, acylation of **1a** with cyclohexanecarboxylic anhydride provides the α-branched ketone **5ac** in 79% yield. This is a successful example of the direct catalytic alkacylation of indolines that yields α-branched ketones, for which efficient coupling reactions have not been reported to date.^12−15^ Benzoic anhydrides and their derivatives also served as good coupling partners for this acylation. Benzoic anhydride reacted smoothly with **1a** to afford the desired product **5ad** in 78% yield. Methyl-substituted benzoic anhydrides rendered the desired products **5ae**–**ag** with good efficiency. Varying the electronic properties of benzoic anhydrides did not significantly affect the reactivity (**5ah**–**aj**). A heteroaroyl group was also introduced into **1a** to form **5ak** in good yield. Thus, our additive-free protocol was applied to a variety of anhydrides without any undesired side reactions.

**Table 3:**
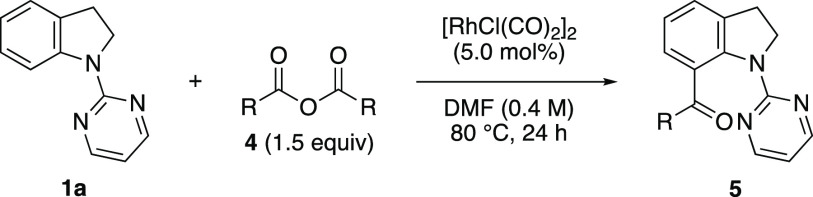

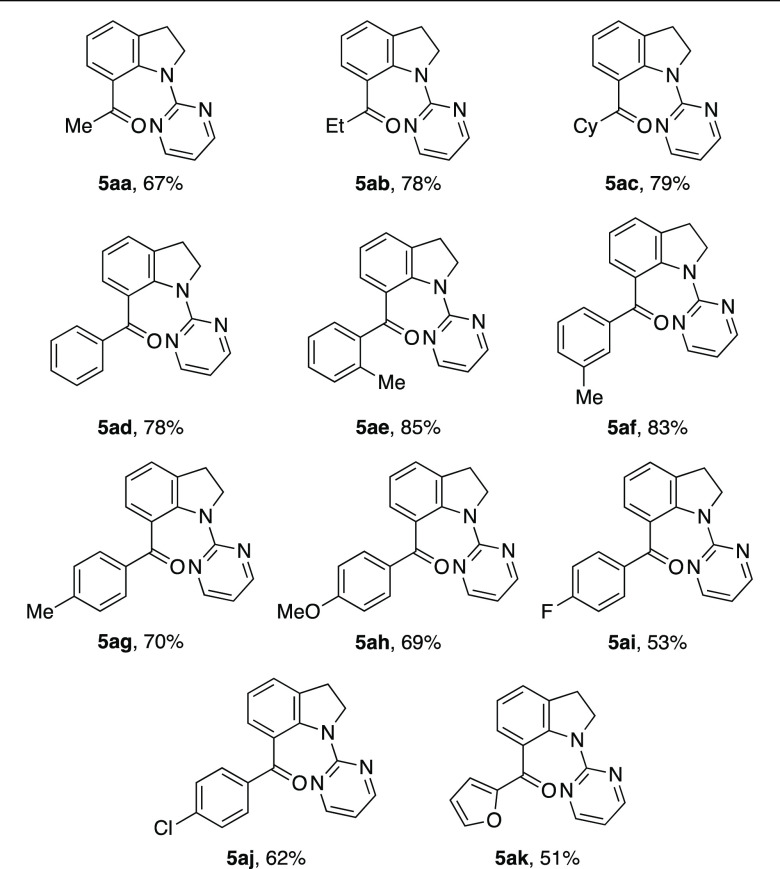
Substrate Scope of Carboxylic Acid Anhydrides^a^

a Reaction conditions: **1a** (0.2 mmol), **4** (0.3 mmol), and [RhCl(CO)_2_]_2_ (5.0 mol %) in DMF (0.5 mL) at 80 °C for 24 h. Isolated yields represent the average of two runs.

In summary, we performed additive-free alkoxycarbonylation of indolines using dialkyl dicarbonates as the alkoxycarbonyl source. Furthermore, this additive-free protocol was applied to the acylation of indolines with a variety of aliphatic and aromatic carboxylic acid anhydrides. Unlike previously reported catalytic reactions, our reaction system achieved the formation of α-branched ketones via the acylation of indolines. We believe that these findings will advance the catalytic alkoxycarbonylation/acylation of C(sp^2^)–H bonds under additive- and CO-free conditions.

## Supporting Information Available

Experimental procedures, characterization data, and copies of NMR spectra for new compounds (PDF)
