# Supplementary material for: Rhodium-Catalyzed C(sp2)–H Alkoxycarbonylation/Acylation of Indolines with Anhydrides as a Carbonyl Source
Source: Org Lett. 2022 Jan 31;24(5):1141–5. doi: 10.1021/acs.orglett.1c04195 (PMC8848290; doi:10.1021/acs.orglett.1c04195)

Supporting Information

Rhodium-Catalyzed C(sp^2)–H Alkoxycarbonylation/Acylation of Indolines with Anhydrides as a Carbonyl Source

Hirotsugu Suzuki, Fumito Sasamori, and Takanori Matsuda*

Department of Applied Chemistry, Tokyo University of Science, 1-3 Kagurazaka, Shinjuku-ku, Tokyo 162-8601, Japan

E-mail: mtd@rs.tus.ac.jp

Table of Contents

1. General Information S1
2. Rhodium-Catalyzed Alkoxycarbonylation of Indolines S2
3. Rhodium-Catalyzed Acylation of Indolines with Carboxylic Acid Anhydrides S8
4. A Large-Scale Synthesis S14
5. Preliminary Mechanistic Studies S15
6. References S18
7. Copies of ^{1}H, ^{13}C, and ^{19}F NMR Spectra for the Products S19

1. General Information

All reactions were performed in oven-dried glassware using Schlenk techniques under argon atmosphere, unless otherwise noted. ^{1}H, ^{13}C and ^{19}F NMR spectra were recorded on a JEOL ECA 500II (500 MHz for ^{1}H, 125 MHz for ^{13}C and 470 MHz for ^{19}F), ESC 300 (300 MHz for ^{1}H and 75 MHz for ^{13}C) or ECZ 400 (400 MHz for ^{1}H and 100 MHz for ^{13}C) spectrometer in $CDCl_3$. Tetramethylsilane (TMS) served as an internal standard (for ^{1}H, $\delta = 0$), and $CDCl_3$ served as an internal standard (for ^{13}C, $\delta = 77.0$). IR spectra were recorded on an FT/IR-4600 (JASCO Co., Ltd.). ESI MS were measured on a Bruker ESI-TOF-MS. Preparative thin-layer chromatography (PTLC) was performed on Wakogel® B-5F. Flash column chromatography was performed on Wakogel® C-200 (75-150μm). Tetrahydrofuran (THF) was purchased from Kanto Chemical as "Dehydrated Solvent System". Other solvents were purchased from FUJIFILM Wako Pure Chemicals and Nacalai Tesque and used without further purification. $RhCl(PPh_3)_3$ was purchase from Sigma-Aldrich. $[RhCl(CO)_2]_2$ was purchased from Kanto Chemical. $[Rh(acac)(CO)_2]_2$ was purchased from Acros Organics. $[Rh(cod)_2]OTf$[1] and $[RhCl(cod)]_2$[2] were synthesized according to the literature. 1-(Pyrimidin-2-yl)indolines **1** was synthesized according to the literature.[3] Diethyl dicarbonate was purchased from BLD Pharmatech Ltd. and used without further purification. Acetic anhydride and propionic anhydride were purchased from FUJIFILM Wako Pure Chemicals and distilled before using. Benzoic anhydride was purchased from Tokyo Chemical Industry. Other carboxylic acid anhydrides were synthesized according to the literature.[4] 1-(Pyrimidin-2-yl)indoline-7-*d* (**1a-*d***) was synthesized according to the literature.[5]

2. Rhodium-Catalyzed Alkoxycarbonylation of Indolines

2.1. General Procedure for the Rhodium-Catalyzed Alkoxycarbonylation of Indolines 1 with Dialkyl Dicarbonates

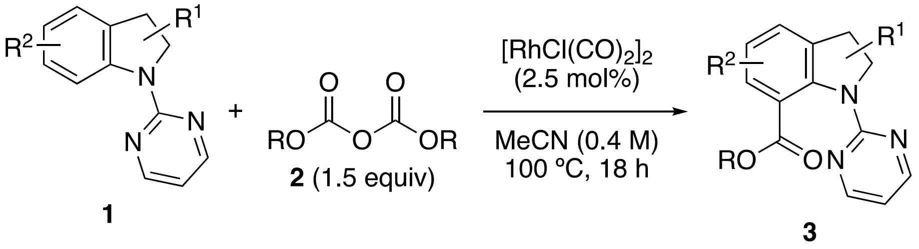

To an oven-dried test tube containing $[RhCl(CO)_2]_2$ (1.9 mg, 5.0×10^{-3} mmol) and indoline **1** (0.2 mmol) in acetonitrile (MeCN, 0.5 mL) was added dialkyl dicarbonate **2** (0.3 mmol) at room temperature and stirred under argon atmosphere at 100 ℃ in an oil bath for 18 h. The reaction mixture was cooled to room temperature and concentrate in *vacuo*. The residue was purified by preparative thin-layer chromatography to give the product **3**.

2.2. Characterization of Products

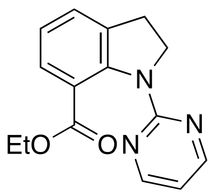

Ethyl 1-(pyrimidin-2-yl)indoline-7-carboxylate (3aa):

The title compound was obtained as a red solid (1st run: 47.9 mg, 89%; 2nd run: 46.1 mg, 86%); Purified by preparative TLC (Hexane/AcOEt = 2:1); mp: 116–117 °C; ^{1}H NMR (500 MHz, $CDCl_3$) δ: 8.39 (d, *J* = 4.6 Hz, 2H), 7.57 (d, *J* = 8.0 Hz, 1H), 7.32 (d, *J* = 7.4 Hz, 1H), 7.01 (t, *J* = 7.7 Hz, 1H), 6.71 (t, *J* = 4.6 Hz, 1H), 4.44 (d, *J* = 8.3 Hz, 2H), 4.00 (q, *J* = 7.3 Hz, 2H), 3.17 (t, *J* = 8.3 Hz, 2H), 0.99 (t, *J* = 7.2 Hz, 3H); ^{13}C NMR (125 MHz, $CDCl_3$) δ: 168.0, 159.8, 156.9, 141.3, 134.5, 127.8, 127.4, 122.3, 121.7, 112.6, 60.3, 50.8, 28.2, 13.7. The spectral data matched those reported in the literature.[6]

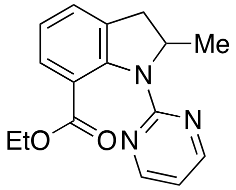

Ethyl 2-methyl-1-(pyrimidin-2-yl)indoline-7-carboxylate (3ba):

The title compound was obtained as a white solid (1st run: 50.2 mg, 89%; 2nd run: 52.9 mg, 93%); Purified by preparative TLC (Hexane/AcOEt = 2:1); mp: 122–123 °C; ^{1}H NMR (500 MHz, $CDCl_3$) δ: 8.37 (d, *J* = 4.6 Hz, 2H), 7.58 (d, *J* = 7.4 Hz, 1H), 7.32 (d, *J* = 7.4 Hz, 1H), 7.02 (t, *J* = 7.4 Hz, 1H), 6.68 (t, *J* = 4.9 Hz, 1H), 4.94–5.00 (m, 1H), 4.09 (ddd, *J* =

6.3, 10.3, 13.7 Hz, 1H), 3.88 (ddd, J = 6.3, 10.3, 13.7 Hz, 1H), 3.45 (dd, J = 16.0, 9.2 Hz, 1H), 2.62 (d, J = 15.5 Hz, 1H), 1.47 (t, J = 7.7 Hz, 3H), 0.97 (t, J = 7.2 Hz, 3H); ^{13}C NMR (125 MHz, $CDCl_3$) δ: 168.1, 159.3, 156.9, 140.1, 133.4, 127.8, 127.7, 122.4, 122.1, 112.5, 60.1, 58.3, 35.9, 21.0, 13.6; IR (neat): $\tilde{\nu}$ = 2976, 1714, 1576, 1555, 1485, 1462, 1438 1289, 1136 cm^{-1}; HRMS (ESI) *m/z*: $[M + Na]^+$ Calcd for $C_{16}H_{17}N_3O_2Na$ 306.1213; found 306.1199.

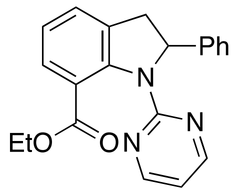

Ethyl 2-phenyl-1-(pyrimidin-2-yl)indoline-7-carboxylate (3ca):

The title compound was obtained as a white solid (1st run: 50.9 mg, 73%; 2nd run: 47.2 mg, 69%); Purified by preparative TLC ($CHCl_3$/AcOEt = 50:1); mp: 176–177 °C; ^{1}H NMR (500 MHz, $CDCl_3$) δ: 8.35 (d, J = 4.6 Hz, 2H), 7.63 (d, J = 7.4 Hz, 1H), 7.49 (d, J = 7.4 Hz, 2H), 7.30 (t, J = 7.7 Hz, 2H), 7.24–7.20 (m, 2H), 7.02 (t, J = 7.4 Hz, 1H), 6.69 (t, J = 4.9 Hz, 1H), 6.03 (d, J = 9.2 Hz, 1H), 4.18 (dq, J = 10.9, 6.9 Hz, 1H), 3.92 (dq, J = 10.9, 7.4 Hz, 1H), 3.83 (dd, J = 15.5, 9.7 Hz, 1H), 3.00 (d, J = 15.5 Hz, 1H), 1.01 (t, J = 7.2 Hz, 3H); ^{13}C NMR (125 MHz, $CDCl_3$) δ: 168.0, 159.4, 157.1, 143.2, 141.0, 132.7, 128.5, 127.8, 127.8, 126.9, 125.4, 123.0, 122.0, 112.9, 64.6, 60.3, 38.2, 13.7; IR (neat): $\tilde{\nu}$ = 2986, 1721, 1575, 1552, 1464, 1435, 1286, 1210, 1133 cm^{-1}; HRMS (ESI) *m/z*: $[M + Na]^+$ Calcd for $C_{21}H_{19}N_3O_2Na$ 368.1369; found 368.1382.

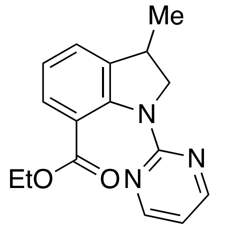

Ethyl 3-methyl-1-(pyrimidin-2-yl)indoline-7-carboxylate (3da):

The title compound was obtained as a red oil (1st run: 45.9 mg, 81%; 2nd run: 45.7 mg, 81%); Purified by preparative TLC (Hexane/AcOEt = 2:1); ^{1}H NMR (500 MHz, $CDCl_3$) δ: 8.39 (d, J = 4.6 Hz, 2H), 7.58 (d, J = 7.4 Hz, 1H), 7.29 (d, J = 7.4 Hz, 1H), 7.05 (t, J = 7.4 Hz, 1H), 6.71 (t, J = 4.6 Hz, 1H), 4.63 (dd, J = 10.9, 9.2 Hz, 1H), 3.95-4.06 (m, 3H), 3.47 (td, J = 14.7, 7.4 Hz, 1H), 1.35 (d, J = 6.9 Hz, 3H), 1.00 (t, J =7.2 Hz, 3H); ^{13}C NMR (125 MHz, $CDCl_3$) δ: 168.2, 159.8, 156.9, 140.8, 139.6, 127.9, 126.3, 122.5, 121.5, 112.5, 60.3, 58.7, 34.9, 19.4, 13.7; IR (neat): $\tilde{\nu}$ = 2961, 2873, 1716, 1577, 1552, 1456, 1431, 1282, 1125 cm^{-1}; HRMS (ESI) *m/z*: $[M + Na]^+$ Calcd for $C_{16}H_{17}N_3O_2Na$ 306.1213; found 306.1218.

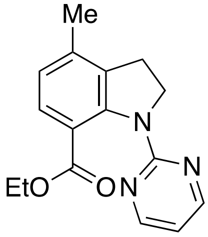

Ethyl 4-methyl-1-(pyrimidin-2-yl)indoline-7-carboxylate (3ea):

The title compound was obtained as a red oil (1st run: 48.3 mg, 85%; 2nd run: 49.4 mg, 87%); Purified by preparative TLC (Hexane/AcOEt = 2:1); ^{1}H NMR (500 MHz, $CDCl_3$) δ: 8.39 (d, J = 4.6 Hz, 2H), 7.51 (d, J = 8.0 Hz, 1H), 6.85 (d, J = 8.0 Hz, 1H), 6.70 (t, J = 4.6 Hz, 1H), 4.45 (t, J = 8.3 Hz, 2H), 3.98 (q, J = 7.1 Hz, 2H), 3.06 (t, J = 8.3 Hz, 2H), 2.27 (s, 3H), 0.97 (t, J =7.2 Hz, 3H); ^{13}C NMR (125 MHz, $CDCl_3$) δ: 168.2, 160.0, 156.9, 141.0, 137.4, 132.9, 128.0, 123.5, 119.3, 112.5, 60.1, 50.7, 27.1, 18.9, 13.7; IR (neat): $\tilde{\nu}$ = 2981, 1715, 1575, 1557, 1463, 1417, 1282, 1127 cm^{-1}; HRMS (ESI) *m/z*: [M + Na]$^+$ Calcd for $C_{16}H_{17}N_3O_2Na$ 306.1213; found 306.1223.

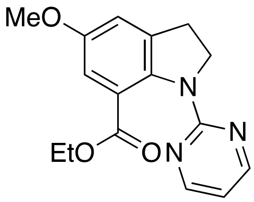

Ethyl 5-methoxy-1-(pyrimidin-2-yl)indoline-7-carboxylate (3fa):

The title compound was obtained as a red oil (1st run: 53.2 mg, 89%; 2nd run: 55.6 mg, 93%); Purified by preparative TLC (Hexane/AcOEt = 2:1); ^{1}H NMR (500 MHz, $CDCl_3$) δ: 8.36 (d, J = 4.6 Hz, 2H), 7.10 (d, J = 2.9 Hz, 1H), 6.93 (m, 1H), 6.67 (t, J = 4.6 Hz, 1H), 4.44 (t, J = 8.0 Hz, 2H), 4.01 (q, J = 7.3 Hz, 2H), 3.81 (s, 3H), 3.12 (t, J =8.3 Hz, 2H), 0.99 (t, J =7.3 Hz, 3H); ^{13}C NMR (125 MHz, $CDCl_3$) δ: 168.0, 159.8, 156.9, 155.4, 136.4, 135.1, 121.9, 115.0, 112.1, 111.1, 60.4, 55.8, 50.8, 28.5, 13.7; IR (neat): $\tilde{\nu}$ = 2979, 1723, 1581, 1552, 1481, 1419, 1380, 1290, 1227, 1124, 1043 cm^{-1}; HRMS (ESI) *m/z*: [M + Na]$^+$ Calcd for $C_{16}H_{17}N_3O_3Na$ 322.1162; found 322.1176.

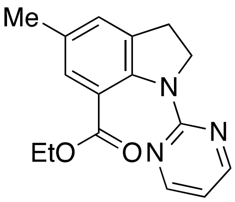

Ethyl 5-methyl-1-(pyrimidin-2-yl)indoline-7-carboxylate (3ga):

The title compound was obtained as a brown oil (1st run: 49.9 mg, 88%; 2nd run: 48.9 mg, 86%); Purified by preparative TLC (Hexane/AcOEt = 2:1); ^{1}H NMR (500 MHz, $CDCl_3$) δ: 8.37 (d, J = 5.2 Hz, 2H), 7.38 (s, 1H), 7.14 (s, 1H), 6.68 (t, J = 4.9 Hz, 1H), 4.42 (t, J = 8.0 Hz, 2H), 4.00 (q, J = 7.1 Hz, 2H), 3.12 (t, J = 8.3 Hz, 2H), 2.33 (s, 3H), 0.99 (t, J =7.2

Hz, 3H); ^{13}C NMR (125 MHz, $CDCl_3$) δ: 168.3, 159.8, 156.9, 139.0, 134.6, 132.2, 128.2, 127.9, 121.4, 112.3, 60.2, 50.8, 28.2, 20.6, 13.7; IR (neat): $\tilde{\nu}$ = 2979, 2900, 1731, 1576, 1548, 1472, 1412, 1275, 1221, 1177 cm^{-1}; HRMS (ESI) *m/z*: [M + Na]$^+$ Calcd for $C_{16}H_{17}N_3O_2Na$ 306.1213; found 306.1219.

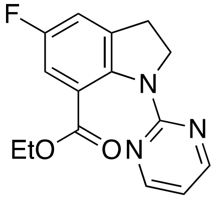

Ethyl 5-fluoro-1-(pyrimidin-2-yl)indoline-7-carboxylate (3ha):

The title compound was obtained as a pale yellow solid (1st run: 51.4 mg, 90%; 2nd run: 49.8 mg, 87%); Purified by preparative TLC (Hexane/Et_2O = 1:3); mp: 129–131 °C; ^{1}H NMR (500 MHz, $CDCl_3$) δ: 8.38 (d, *J* = 4.6 Hz, 2H), 7.28–7.26 (m, 1H), 7.05 (dt, *J* = 7.6, 1.3 Hz, 1H), 6.72 (t, *J* = 4.6 Hz, 1H), 4.47 (t, *J* = 8.3 Hz, 2H), 4.02 (q, *J* = 7.1 Hz, 2H), 3.15 (t, *J* = 8.3 Hz, 2H), 1.01 (t, *J* = 7.2 Hz, 3H); ^{13}C NMR (125 MHz, $CDCl_3$) δ: 167.0 ($J_{C–F}$ = 2.4 Hz), 159.7, 158.2 ($J_{C–F}$ = 239.6 Hz), 156.9, 137.6 ($J_{C–F}$ = 2.4 Hz), 136.8 ($J_{C–F}$ = 8.4 Hz), 122.3 ($J_{C–F}$ = 7.1 Hz), 115.9 ($J_{C–F}$ = 23.9 Hz), 114.9 ($J_{C–F}$ = 25.0 Hz), 112.7, 60.6, 51.0, 28.4 ($J_{C–F}$ = 2.4 Hz), 13.7; ^{19}F NMR (470 MHz, $CDCl_3$) δ: −120.65; IR (neat): $\tilde{\nu}$ = 2979, 1718, 1576, 1555, 1455, 1414, 1289, 1202, 1168 cm^{-1}; HRMS (ESI) *m/z*: [M + Na]$^+$ Calcd for $C_{15}H_{14}N_3O_2FNa$ 310.0962; found 310.0965.

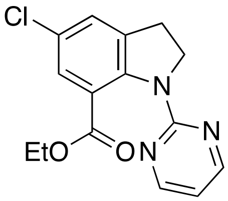

Ethyl 5-chloro-1-(pyrimidin-2-yl)indoline-7-carboxylate (3ia):

The title compound was obtained as a brown oil (1st run: 53.2 mg, 88%; 2nd run: 50.3 mg, 83%); Purified by preparative TLC (Hexane/AcOEt = 2:1); ^{1}H NMR (500 MHz, $CDCl_3$) δ: 8.39 (d, *J* = 4.6 Hz, 2H), 7.54 (d, *J* = 2.3 Hz, 1H), 7.28 (br s, 1H), 6.74 (t, *J* = 4.9 Hz, 1H), 4.45 (t, *J* = 8.3 Hz, 2H), 4.01 (q, *J* = 7.3 Hz, 2H), 3.15 (t, *J* = 8.6 Hz, 2H), 0.99 (t, *J* = 7.2 Hz, 3H); ^{13}C NMR (125 MHz, $CDCl_3$) δ: 166.9, 159.5, 156.9, 140.1, 136.6, 127.4, 127.3, 127.1, 122.4, 112.9, 60.6, 50.9, 28.0, 13.7; IR (neat): $\tilde{\nu}$ = 2980, 1727, 1578, 1556, 1471, 1413, 1261, 1152 cm^{-1}; HRMS (ESI) *m/z*: [M + Na]$^+$ Calcd for $C_{15}H_{14}N_3O_2ClNa$: 326.0667; found: 326.0655.

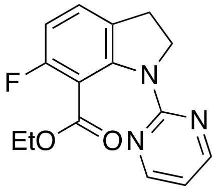

Ethyl 6-fluoro-1-(pyrimidin-2-yl)indoline-7-carboxylate (3ja):

The title compound was obtained as a pale yellow oil (1st run: 51.5 mg, 90%; 2nd run: 53.3 mg, 93%); Purified by preparative TLC (Hexane/Et_2O = 1:3); ^1H NMR (500 MHz, $CDCl_3$) δ: 8.40 (d, J = 4.6 Hz, 2H), 7.19 (dd, J = 8.6, 5.2 Hz, 1H), 6.76–6.69 (m, 2H), 4.44 (t, J = 8.3 Hz, 2H), 4.07 (q, J = 7.3 Hz, 2H), 3.11 (t, J = 8.3 Hz, 2H), 1.09 (t, J = 7.2 Hz, 3H); ^{13}C NMR (125 MHz, $CDCl_3$) δ: 164.1, 159.9 ($J_{C–F}$ = 248.0 Hz), 159.1, 156.9, 142.1($J_{C–F}$ = 6.0 Hz), 129.5 ($J_{C–F}$ = 2.4 Hz), 126.7 ($J_{C–F}$ = 10.8 Hz), 112.9, 111.2 ($J_{C–F}$ = 17.9 Hz), 109.2 ($J_{C–F}$ = 23.9 Hz), 60.6, 51.3, 27.5, 13.8; ^{19}F NMR (470 MHz, $CDCl_3$) δ: −117.53; IR (neat): $\tilde{\nu}$ = 2987, 2900, 1714, 1557, 1470, 1438, 1113 cm^{-1}; HRMS (ESI) *m/z*: $[M + Na]^+$ Calcd for $C_{15}H_{14}N_3O_2FNa$ 310.0962; found 310.0959.

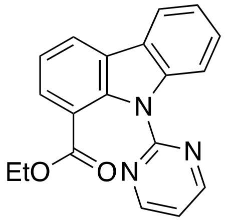

Ethyl 9-(pyrimidin-2-yl)-9*H*-carbazole-1-carboxylate (3la):

The title compound was obtained as a red oil (1st run: 24.1 mg, 38%; 2nd run: 21.4 mg, 34%); Purified by preparative TLC ($CHCl_3$/AcOEt = 50:1); ^1H NMR (500 MHz, $CDCl_3$) δ: 8.79 (d, J = 4.6 Hz, 2H), 8.49 (d, J = 8.6 Hz, 1H), 8.21 (dd, J = 7.4, 1.1 Hz, 1H), 8.07 (d, J = 7.4 Hz, 1H), 7.90 (dd, J = 7.4, 1.1 Hz, 1H), 7.49 (td, J = 7.7, 1.1 Hz, 1H), 7.38–7.40 (m, 2H), 7.16 (t, J = 4.9 Hz, 1H), 3.91 (q, J = 7.1 Hz, 2H), 1.04 (t, J = 7.5 Hz, 3H); ^{13}C NMR (125 MHz, $CDCl_3$) δ: 167.7, 159.4, 157.8, 140.6, 136.6, 127.9, 127.2, 127.2, 124.8, 123.3, 122.5, 121.7, 120.2, 119.8, 117.4, 113.8, 60.7, 13.8; IR (neat): $\tilde{\nu}$ = 3057, 2981, 1724, 1566, 1452, 1425, 1280, 1140 cm^{-1}; HRMS (ESI) *m/z*: $[M + Na]^+$ Calcd for $C_{19}H_{15}N_3O_2Na$ 340.1056; found 340.1043.

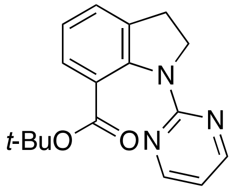

***tert*-Butyl 1-(pyrimidin-2-yl)indoline-7-carboxylate (3ab):**

The title compound was obtained as a colorless oil (1st run: 38.9 mg, 66%; 2nd run: 41.4 mg, 70%); Purified by preparative TLC (Hexane/AcOEt = 4:1); ^1H NMR (500 MHz, $CDCl_3$) δ: 8.41 (d, J = 5.2 Hz, 2H), 7.57 (d, J = 7.4 Hz, 1H), 7.30 (d,

J = 7.4 Hz, 1H), 7.00 (t, *J* = 7.4 Hz, 1H), 6.70 (t, *J* = 4.6 Hz, 1H), 4.44 (t, *J* = 8.3 Hz, 2H), 3.13 (t, *J* = 8.3 Hz, 2H), 1.38 (s, 9H); ^{13}C NMR (125 MHz, $CDCl_3$) δ: 166.7, 160.3, 157.3, 141.5, 134.7, 127.9, 127.2, 123.2, 122.4, 112.6, 80.5, 51.2, 28.4, 28.0. The spectral data matched those reported in the literature.[6]

3. Rhodium-Catalyzed Acylation of Indolines with Carboxylic Acid Anhydrides

3.1. Optimization of Reaction Conditions for Rhodium-Catalyzed Acylation of 1-(Pyrimidin-2-yl)indoline (1a) with Acetic Anhydride (4a)[a]

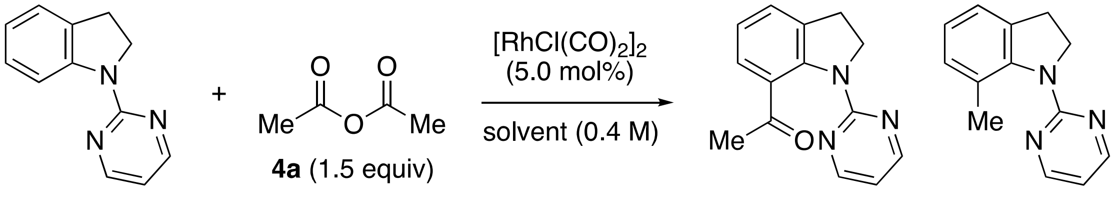

1a **5aa** **5aa′**

entry	solvent	temperature (°C)	time (h)	yield of **5aa** (%)[b]	yield of **5aa′** (%)[b]
1	DME	100	18	38	<5
2	DCE	100	18	16	trace
3	DMF	100	18	51	trace
4	toluene	100	18	31	trace
5	THF	100	18	21	trace
6	1,4-dioxane	100	18	49	6
7	MeCN	100	18	33	trace
8	DMF	130	18	54	18
9[c]	DMF	100	18	68	8
10[c]	DMF	80	18	69	0
11[c]	DMF	80	24	80 (67)	0

[a] Reaction conditions: **1a** (0.2 mmol), **4a** (0.3 mmol), and $[RhCl(CO)_2]_2$ (2.5 mol%) in the solvent (0.5 mL) unless otherwise noted. [b] Yields were determined by ^{1}H NMR analysis using 1,1,2,2-tetrachloroethane as an internal standard. Value in parentheses indicates isolated yield, which represents the average of two runs. [c] 5.0 mol% of $[RhCl(CO)_2]_2$ was used.

3.2. General Procedure for the Rhodium-Catalyzed Acylation of 1-(Pyrimidin-2-yl)indoline (1a) with Carboxylic Acid Anhydrides 4

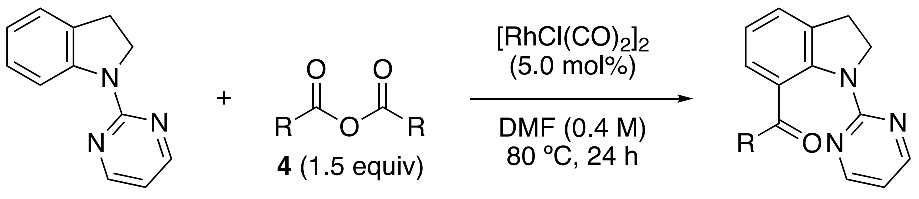

1a **5**

An oven-dried test tube was charged with $[RhCl(CO)_2]_2$ (3.9 mg, 1.0 × 10^{-2} mmol), 1-(pyrimidin-2-yl)indoline (**1a**, 39.4 mg, 0.200 mmol), carboxylic acid anhydride **4** (0.3 mmol) and DMF (0.5 mL). The solution was stirred under argon atmosphere at 80 °C in an oil bath. After 24 h, the reaction mixture was cooled to room temperature and quenched with 1 M NaOH solution (1.0 mL). The mixture was extracted with EtOAc (15 mL × 3) and washed H_2O (10 mL×3). The organic phase was dried over Na_2SO_4 and concentrated in *vacuo*. The residue was purified by preparative thin-layer chromatography to give the product **5**.

3.3. Characterization of Products

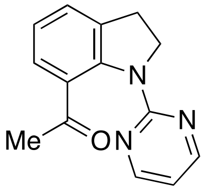

1-[1-(Pyrimidin-2-yl)indolin-7-yl]ethan-1-one (5aa):

The title compound was obtained as a brown oil (1st run: 31.3 mg, 66%; 2nd run: 32.3 mg, 68%); Purified by preparative TLC ($CHCl_3$/AcOEt = 20:1); ^{1}H NMR (300 MHz, $CDCl_3$) δ: 8.41 (d, *J* = 4.8 Hz, 2H), 7.36 (d, *J* = 7.6 Hz, 1H), 7.31 (d, *J* = 7.6 Hz, 1H), 7.04 (t, *J* = 7.6 Hz, 1H), 6.74 (t, *J* = 4.8 Hz, 1H), 4.47 (t, *J* = 8.4 Hz, 2H), 3.20 (t, 8.3 Hz, 2H), 2.33 (s, 3H); ^{13}C NMR (125 MHz, $CDCl_3$) δ: 200.1, 158.9, 156.9, 139.9, 134.2, 130.2, 126.8, 125.6, 122.7, 113.0, 49.9, 28.9, 28.2. The spectral data matched those reported in the literature.[7]

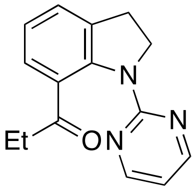

1-[1-(Pyrimidin-2-yl)indolin-7-yl]propan-1-one (5ab):

The title compound was obtained as an orange solid (1st run: 39.4 mg, 78%; 2nd run: 38.7 mg, 77%); Purified by preparative TLC ($CHCl_3$/AcOEt = 4:1); mp: 127–128 °C; ^{1}H NMR (500 MHz, $CDCl_3$) δ: 8.35 (d, *J* = 5.2 Hz, 2H), 7.30 (dt, *J* = 10.5, 4.6 Hz, 2H), 7.03 (dd, *J* = 9.5, 5.4 Hz, 1H), 6.71 (t, *J* = 4.9 Hz, 1H), 4.45 (t, *J* = 8.0 Hz, 2H), 3.19 (t, *J* =

8.0 Hz, 2H), 2.67 (q, J = 7.3 Hz, 2H), 1.03 (t, J = 7.2 Hz, 3H); ^{13}C NMR (125 MHz, $CDCl_3$) δ: 203.2, 158.9, 157.0, 139.8, 134.1, 129.9, 126.6, 125.9, 122.8, 112.8, 49.8, 34.4, 28.2, 8.2; IR (neat): $\tilde{\nu}$ = 2980, 2937, 2890, 1686, 1451,1426, 1038, 808, 758 cm^{-1}; HRMS (ESI) *m/z*: $[M + H]^+$ Calcd for $C_{15}H_{16}N_3O$ 254.1288; found: 254.1299.

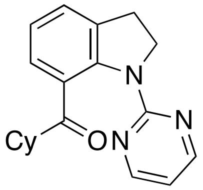

Cyclohexyl[1-(pyrimidin-2-yl)indolin-7-yl]methanone (5ac):

The title compound was obtained as a pale yellow oil (1st run: 49.5 mg, 80%; 2nd run: 48.1 mg, 78%); Purified by preparative TLC (Hexane/AcOEt = 4:1); ^{1}H NMR (500 MHz, $CDCl_3$) δ: 8.36 (d, J = 4.6 Hz, 2H), 7.32–7.27 (m, 2H), 7.02 (t, J = 8.0 Hz, 1H), 6.72 (t, J = 4.9 Hz, 1H), 4.46 (t, J = 8.0 Hz, 2H), 3.19 (t, J = 8.3 Hz, 2H), 2.66 (tt, J = 11.5, 3.2 Hz, 1H), 1.68–1.64 (m, 2H), 1.59–1.54 (m, 3H), 1.35–1.27 (m, 2H), 1.16–1.02 (m, 3H); ^{13}C NMR (125 MHz, $CDCl_3$) δ: 205.4, 159.1, 157.2, 139.8, 133.9, 129.1, 127.2, 126.5, 122.8, 112.8, 49.7, 48.4, 28.8, 28.2, 26.0, 25.8; IR (neat): $\tilde{\nu}$ = 2934, 2856, 1686, 1579, 1556, 1481, 1460, 1282 cm^{-1}; HRMS (ESI) *m/z*: $[M + H]^+$ Calcd for $C_{19}H_{22}N_3O$ 308.1757; found 308.1746.

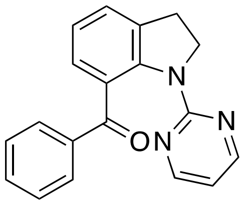

Phenyl[1-(pyrimidin-2-yl)indolin-7-yl]methanone (5ad):

The title compound was obtained as a yellow solid (1st run: 47.6 mg, 79%; 2nd run: 46.5 mg, 77%); Purified by preparative TLC ($CHCl_3$/AcOEt = 9:1); mp: 116–117 °C; ^{1}H NMR (500 MHz, $CDCl_3$) δ: 7.94 (d, J = 4.0 Hz, 2H), 7.88–7.89 (m, 2H), 7.46–7.48 (m, 1H), 7.39 (t, J = 7.4 Hz, 2H), 7.33 (dd, J = 7.4, 1.1 Hz, 1H), 7.22 (d, J = 7.4 Hz, 1H), 7.01 (t, J = 7.4 Hz, 1H), 6.47 (t, J = 4.9 Hz, 1H), 4.40 (t, J = 8.0 Hz, 2H), 3.24 (t, J = 8.3 Hz, 2H); ^{13}C NMR (125 MHz, $CDCl_3$) δ: 194.4, 157.8, 156.4, 140.6, 137.7, 134.0, 131.8, 129.7, 127.9, 127.3, 127.1, 126.4, 121.9, 112.5, 49.5, 28.3. The spectral data matched those reported in the literature.[7]

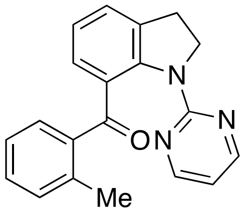

[1-(Pyrimidin-2-yl)indolin-7-yl](*o*-tolyl)methanone (5ae):

The title compound was obtained as a yellow solid (1st run: 54.0 mg, 86%; 2nd run: 52.0 mg, 83%); Purified by preparative TLC (Hexane/AcOEt = 2:1); mp: 123–125 °C;^{1}H NMR (500 MHz, $CDCl_3$) δ: 8.12 (d, J = 4.6 Hz, 2H), 7.70 (t, J = 4.0 Hz, 1H), 7.30–7.34 (m, 2H), 7.20 (t, J = 7.4 Hz, 2H), 7.14–7.17 (m, 1H), 6.97 (t, J = 7.4 Hz, 1H), 6.53 (t, J = 4.6 Hz, 1H), 4.42 (t, J = 8.0 Hz, 2H), 4.42 (t, J = 8.3 Hz, 2H), 2.51 (s, 3H); ^{13}C NMR (125 MHz, $CDCl_3$) δ: 196.7, 158.6, 156.6, 141.1, 139.4, 136.7, 134.4, 131.5, 131.3, 130.8, 129.0, 127.8, 126.9, 124.9, 122.1, 112.6, 50.3, 28.3, 21.0; IR (neat): $\tilde{\nu}$ = 3299, 3036, 2961, 1660, 1575, 1456, 1258, 1003, 734 cm^{-1}; HRMS (ESI) *m/z*: $[M + H]^+$ Calcd for $C_{20}H_{18}N_3O$ 316.1444; found 316.1431.

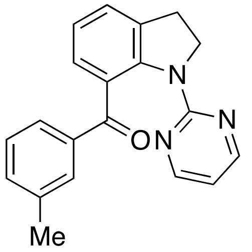

[1-(Pyrimidin-2-yl)indolin-7-yl](*m*-tolyl)methanone (5af):

The title compound was obtained as a yellow amorphous (1st run: 52.9 mg, 84%; 2nd run: 51.4mg, 82%); Purified by preparative TLC (Hexane/AcOEt = 2:1); ^{1}H NMR (500 MHz, $CDCl_3$) δ: 7.95 (d, J = 4.6 Hz, 2H), 7.73 (br s, 1H), 7.68–7.70 (m, 1H), 7.33 (dd, J = 7.4, 1.1 Hz, 1H), 7.27–7.30 (m, 2H), 7.20 (d, J = 7.4 Hz, 1H), 7.00 (t, J = 7.7 Hz, 1H), 6.48 (t, J = 4.9 Hz, 1H), 4.38–4.42 (m, 2H), 3.25 (t, J = 8.6 Hz, 2H), 2.37 (s, 3H); ^{13}C NMR (125 MHz, $CDCl_3$) δ: 194.6, 157.8, 156.4, 140.5, 137.7, 137.6, 134.0, 132.6, 130.2, 127.7, 127.5, 127.2, 127.1, 126.3, 121.8, 112.5, 49.5, 28.2, 21.3; IR (neat): $\tilde{\nu}$ = 3044, 2957, 2892, 1653, 1598, 1580, 1557, 1456, 1435, 1286, 1135 cm^{-1}; HRMS (ESI) *m/z*: $[M + Na]^+$ Calcd for $C_{20}H_{17}N_3ONa$ 338.1264; found 338.1274.

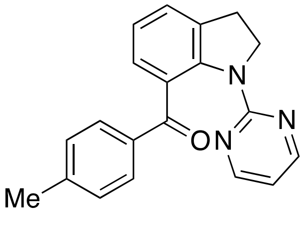

[1-(Pyrimidin-2-yl)indolin-7-yl](*p*-tolyl)methanone (5ag):

The title compound was obtained as a yellow solid (1st run: 45.5 mg, 72%; 2nd run: 42.9 mg, 68%); Purified by preparative TLC (Hexane/AcOEt = 2:1); mp: 153–154 °C; ^{1}H NMR (500 MHz, $CDCl_3$) δ: 7.96 (d, J = 4.0 Hz, 2H), 7.79 (d, J = 8.0 Hz, 2H), 7.32 (d, J = 6.9 Hz, 1H), 7.20 (t, J = 6.9 Hz, 3H), 7.01 (t, J = 7.4 Hz, 1H), 6.48 (t, J = 4.9 Hz, 1H), 4.40 (t, J = 8.3 Hz, 2H), 3.25 (t, J = 8.3 Hz, 2H), 2.39 (s, 3H); ^{13}C NMR (125 MHz, $CDCl_3$) δ: 194.3, 157.8, 156.4, 142.4, 140.5, 135.2, 134.0, 129.9, 128.6, 127.6, 127.1, 126.3, 121.8, 112.5, 49.5, 28.3, 21.6. The spectral data matched those reported in the literature.[7]

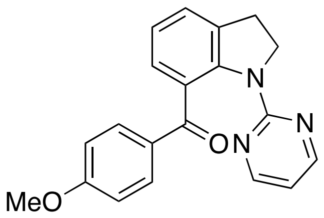

(4-Methoxyphenyl)[1-(pyrimidin-2-yl)indolin-7-yl]methanone (5ah):

The title compound was obtained as a colorless solid (1st run: 47.2 mg, 71%; 2nd run: 44.4 mg, 67%); Purified by preparative TLC (Hexane/AcOEt = 1:1); mp: 158–159 °C; ^{1}H NMR (500 MHz, $CDCl_3$) δ: 7.96 (d, J = 4.6 Hz, 2H), 7.86–7.88 (m, 2H), 7.32 (dd, J = 6.9, 1.1 Hz, 1H), 7.21 (d, J = 6.9 Hz, 1H), 7.01 (t, J = 7.4 Hz, 1H), 6.89 (d, J = 4.9 Hz, 2H), 4.40 (t, J = 8.3 Hz, 2H), 3.85 (s, 3H), 3.24 (t, J = 8.3 Hz, 3H); ^{13}C NMR (125 MHz, $CDCl_3$) δ: 193.6, 162.5, 157.9, 156.4, 140.5, 134.0, 131.9, 130.7, 127.6, 127.1, 126.2, 121.9, 113.1, 112.5, 55.3, 49.6, 28.3. The spectral data matched those reported in the literature.[7]

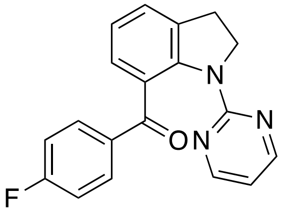

(4-Fluorophenyl)[1-(pyrimidin-2-yl)indolin-7-yl]methanone (5ai):

The title compound was obtained as a pale yellow solid (1st run: 35.0 mg, 55%; 2nd run: 32.8 mg, 51%); Purified by preparative TLC (Hexane/AcOEt = 2:1); mp: 177–178 °C; ^{1}H NMR (500 MHz, $CDCl_3$) δ: 7.97–7.90 (m, 4H), 7.35 (d, J = 6.9 Hz, 1H), 7.20 (d, J = 7.4 Hz, 1H), 7.09–7.01 (m, 3H), 6.51 (t, J = 4.9 Hz, 1H), 4.41 (t, J = 8.3 Hz, 2H), 3.26 (t, J = 8.3 Hz, 2H); ^{13}C NMR (125 MHz, $CDCl_3$) δ: 193.0, 164.8 ($J_{C–F}$ = 253.0 Hz), 157.8, 156.4, 140.5, 134.1, 134.1 ($J_{C–F}$ = 4.8 Hz), 132.2 ($J_{C–F}$ = 9.7 Hz), 127.0, 127.0, 126.6, 122.0, 115.0 ($J_{C–F}$ = 21.5 Hz), 112.6, 49.5, 28.2; ^{19}F NMR (470 MHz, $CDCl_3$) δ: −107.1; IR (neat): $\tilde{\nu}$ = 3059, 2897, 1659, 1600, 1462, 1274, 1222, 1009 cm^{-1}; HRMS (ESI) m/z: $[M + H]^+$ Calcd for $C_{19}H_{15}N_3OF$ 320.1194; found 320.1192.

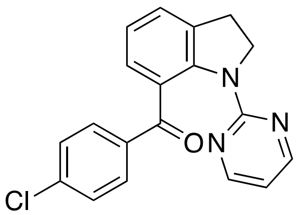

(4-Chlorophenyl)[1-(pyrimidin-2-yl)indolin-7-yl]methanone (5aj):

The title compound was obtained as a yellow solid (1st run: 42.0 mg, 63%; 2nd run: 40.4 mg, 60%); Purified by preparative TLC (Hexane/AcOEt = 2:1); mp: 135–136 °C; ^{1}H NMR (500 MHz, $CDCl_3$) δ: 7.97 (d, J = 4.6 Hz, 2H), 7.83–7.84 (m, 2H), 7.35–7.37 (m, 3H), 7.19 (t, J = 4.0 Hz, 1H), 7.02 (t, J = 7.4 Hz, 1H), 6.51 (t, J = 4.6 Hz, 1H), 4.41 (t, J = 8.0 Hz, 2H), 3.26 (t, J = 8.3 Hz, 2H). ^{13}C NMR (125 MHz, $CDCl_3$) δ: 193.1, 157.7, 156.4, 140.5, 138.0, 136.2, 134.1, 131.1, 128.2, 126.9, 126.8, 126.6, 122.0, 112.7, 49.5, 28.2; IR (neat): $\tilde{\nu}$ = 2926, 1654, 1580, 1556, 1473, 1456, 1436, 1284, 1090 cm^{-1}; HRMS (ESI) *m/z*: $[M + Na]^+$ Calcd for $C_{19}H_{14}N_3OClNa$ 358.0718; found 358.0717.

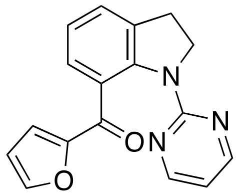

Furan-2-yl[1-(pyrimidin-2-yl)indolin-7-yl]methanone (5ak):

The title compound was obtained as a pale yellow oil (1st run: 29.7 mg, 51%; 2nd run: 29.4 mg, 51%); Purified by preparative TLC (Hexane/AcOEt = 2:3); ^{1}H NMR (500 MHz, $CDCl_3$) δ: 8.11 (d, J = 4.6 Hz, 2H), 7.53 (s, 1H), 7.38 (d, J = 7.4 Hz, 1H), 7.35 (d, J = 7.4 Hz, 1H), 7.06–7.01 (m, 2H), 6.55 (t, J = 4.9 Hz, 1H), 6.45 (dd, J = 3.4, 1.7 Hz, 1H), 4.41 (t, J = 8.3 Hz, 2H), 3.24 (t, J = 8.3 Hz, 2H); ^{13}C NMR (125 MHz, $CDCl_3$) δ: 182.3, 157.9, 156.6, 153.2, 145.9, 140.2, 134.2, 127.1, 126.9, 126.2, 122.1, 117.1, 112.5, 111.8, 49.6, 28.2; IR (neat): $\tilde{\nu}$ = 3134, 2914, 652, 1575, 1464, 1382, 1273, 1023 cm^{-1}; HRMS (ESI) *m/z*: $[M + Na]^+$ Calcd for $C_{17}H_{13}N_3O_2Na$ 314.0900 ; found 314.0904.

4. A Large-Scale Synthesis

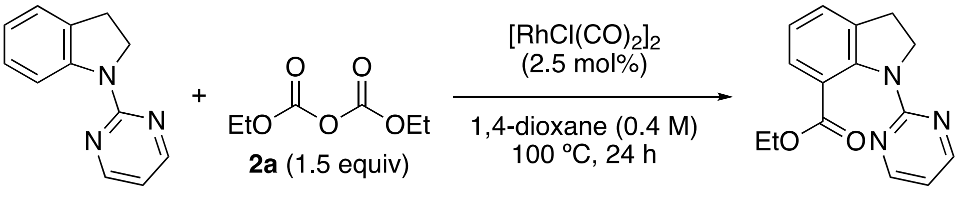

1a
(5 mmol)

3aa, 72% yield
(0.97 g)

To an oven-dried test tube containing $[RhCl(CO)_2]_2$ (48.3 mg, 0.124 mmol) and 1-(pyrimidin-2-yl)indoline (**1a**, 987.4 mg, 5.006 mmol) in 1,4-dioxane (12.5 mL) was added diethyl dicarbonate (**2a**, 1.21 g, 7.46 mmol) at room temperature and stirred under argon atmosphere at 100 ℃ in an oil bath for 24 h. The reaction mixture was cooled to room temperature and concentrate in *vacuo*. The residue was purified by flash column chromatography (hexane/EtOAc = 2:1) to give the product **3aa** (965.5 mg, 3.585 mmol, 72%).

5. Preliminary Mechanistic Studies

5.1. H/D Exchange Experiment

To an oven-dried test tube containing $[RhCl(CO)_2]_2$ (1.9 mg, 5.0×10^{-3} mmol) and 1-(pyrimidin-2-yl)indoline (**1a**, 39.4 mg, 0.200 mmol) in acetonitrile (MeCN, 0.5 mL) was added D_2O (20.1 mg, 1.00 mmol) at room temperature and stirred under argon atmosphere at 100 °C in an oil bath for 18 h. The reaction mixture was cooled to room temperature and concentrate in *vacuo*. The residue was purified by preparative thin-layer chromatography (hexane/EtOAc = 2:1) to give the product **1a-*d*** (35.8 mg, 90%) as a white solid.

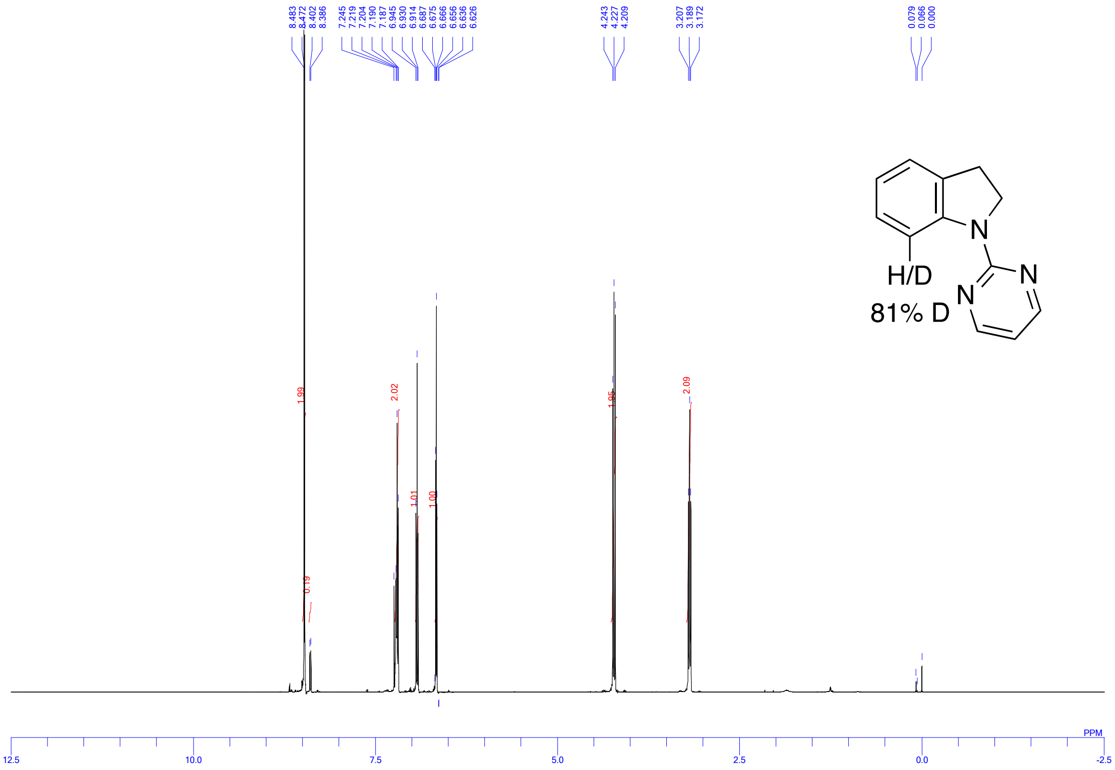

To an oven-dried test tube containing $[RhCl(CO)_2]_2$ (1.9 mg, 5.0 × 10^{-3} mmol) and 1-(pyrimidin-2-yl)indoline (**1a**, 39.4 mg, 0.200 mmol) in acetonitrile (MeCN, 0.5 mL) was added diethyl dicarbonate (**2a**, 48.7 mg, 0.300 mmol) and D_2O (20.2 mg, 1.01 mmol) at room temperature and stirred under argon atmosphere at 100 ℃ in an oil bath for 3 h. The reaction mixture was cooled to room temperature and concentrate in *vacuo*. The residue was purified by preparative thin-layer chromatography (hexane/EtOAc) to give the product **1a-*d*** (18.5 mg, 47%) as a white solid and the product **3aa** (27.4 mg, 51%) as a white solid.

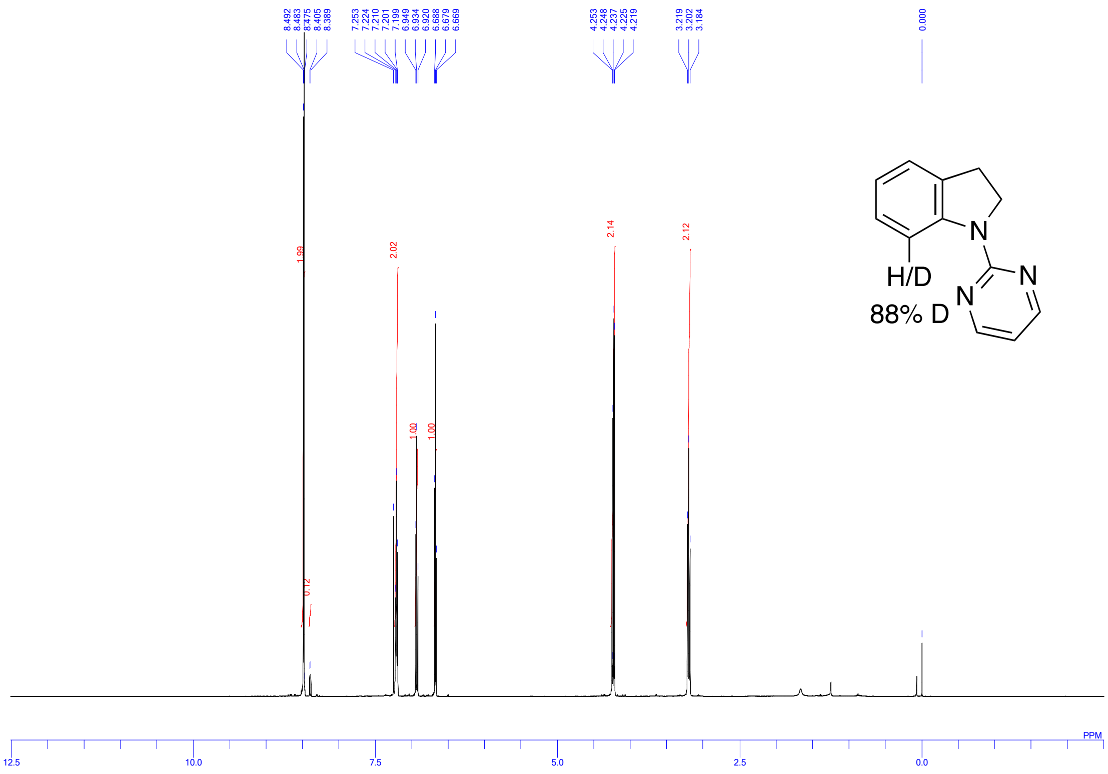

5.2. KIE Experiment

Kinetic isotope effect (KIE) was measured by two sets of parallel experiments using 1-(pyrimidin-2-yl)indoline **1a** and **1a-*d***. To an oven-dried test tube equipped with a stirring bar charged with 1-(pyrimidin-2-yl)indoline (**1a**, 39.5 mg, 0.200 mmol) and $[RhCl(CO)_2]_2$ (1.9 mg, 5.0×10^{-3} mmol) was added MeCN (0.5 mL) followed by the addition of diethyl dicarbonate (**2a**, 47.6 mg, 0.294 mmol). To another oven-dried test tube equipped with a stirring bar charged with 1-(pyrimidin-2-yl)indoline-7-*d* (**1a-*d***, 39.6 mg, 0.200 mmol) and $[RhCl(CO)_2]_2$ (1.9 mg, 5.0×10^{-3} mmol) was added MeCN (0.5 mL) followed by the addition of diethyl dicarbonate (**2a**, 48.5 mg, 0.299 mmol). Both reactions were allowed to stir at 100 °C in an oil bath for 15 min. The resulting solutions were concentrated in *vacuo*. The ^{1}H NMR yields of **3aa** for each reaction were given using 1,2,4,5-tetramethylbenzene as an internal standard. The yields were 36.2% and 36.3% respectively, and a KIE value of 1.0 was determined on the basis of the ^{1}H NMR yields.

6. References

1. Vilhanová, B.; Ranocchiari, M.; van Bokhoven, J. A. *ChemCatChem* **2016**, *8*, 308–312.
2. Pritzuis, A. B.; Breit, B. *Angew. Chem., Int. Ed.* **2015**, *54*, 3121–3125.
3. Leitch, J. A.; Heron, C. J.; McKnight, J.; Kociok-Köhn, G.; Bhonoah, Y.; Frost, C. G. *Chem. Commun.* **2017**, *53*, 13039–13042.
4. Qiu, X.; Wang, P.; Wang, D.; Wang, M.; Yuan Y.; Shi, Z. *Angew. Chem., Int. Ed.* **2019**, *58*, 1504–1508.
5. Xie, W.; Li, B.; Wang, B. *J. Org. Chem.* **2016**, *81*, 396–403.
6. Du, R.; Zhao, K.; Liu, J.; Han, F.; Xia C.; Yang, L. *Org. Lett.* **2019**, *21*, 6418–6422.
7. Xie, G.; Zhao, Y.; Cai, C.; Deng G.-J.; Gong, H. *Org. Lett.* **2021**, *23*, 410–415.

7. Copies of ^{1}H, ^{13}C and ^{19}F NMR Spectra for the Products

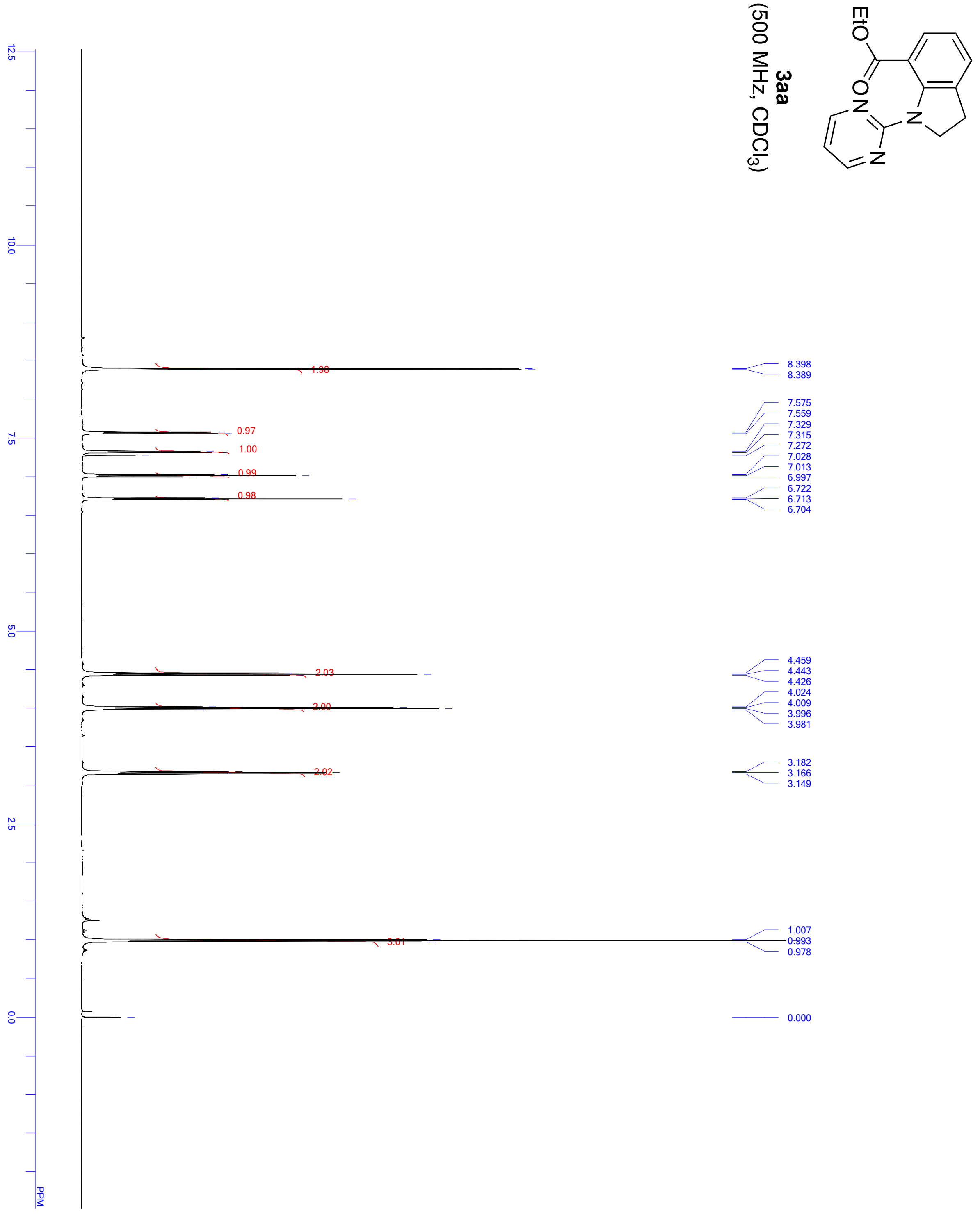

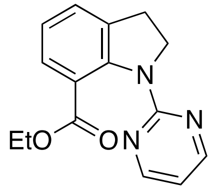

3aa
(125 MHz, $CDCl_3$)

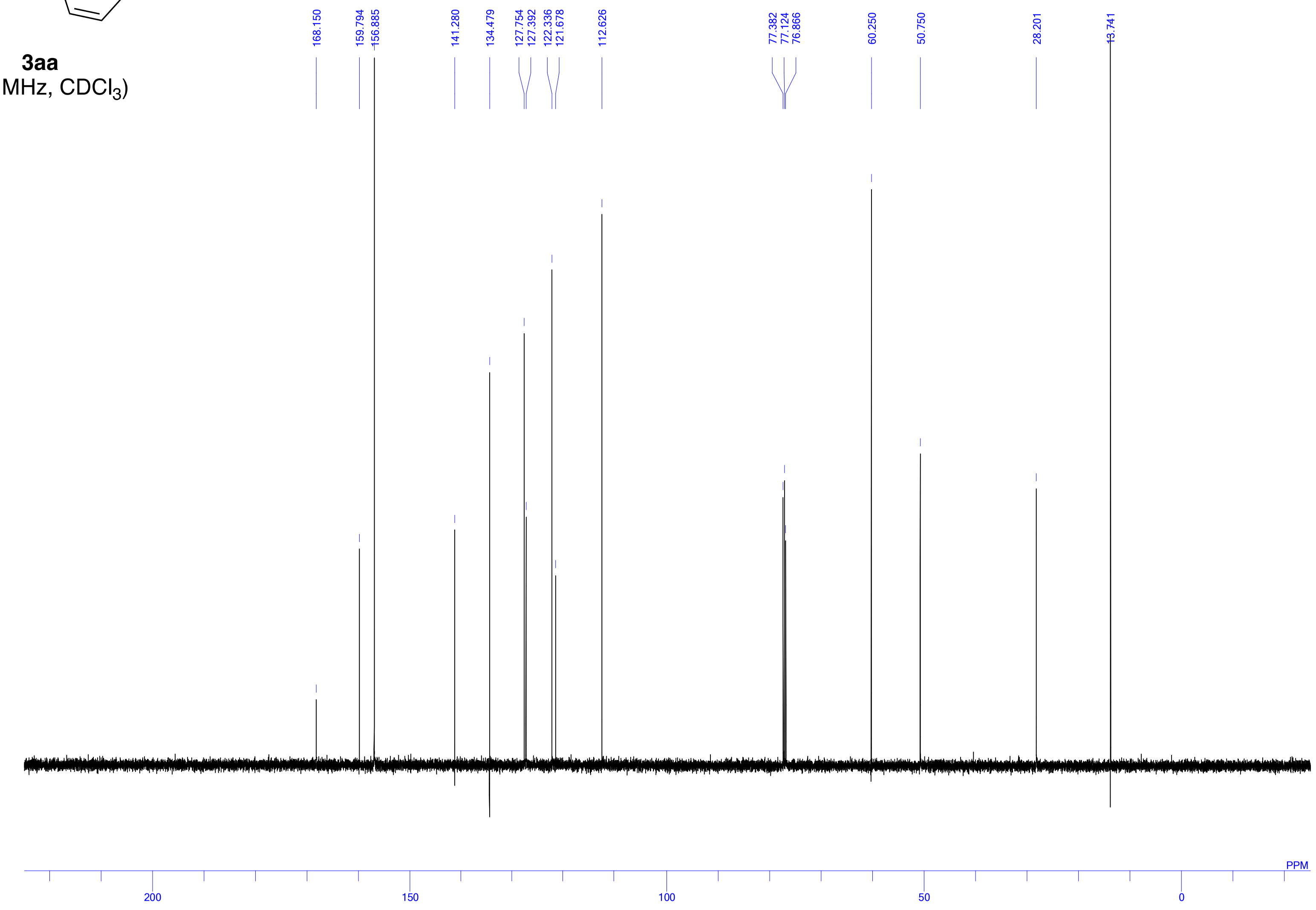

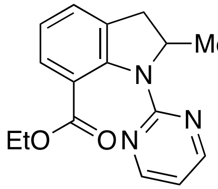

3ba
(500 MHz, $CDCl_3$)

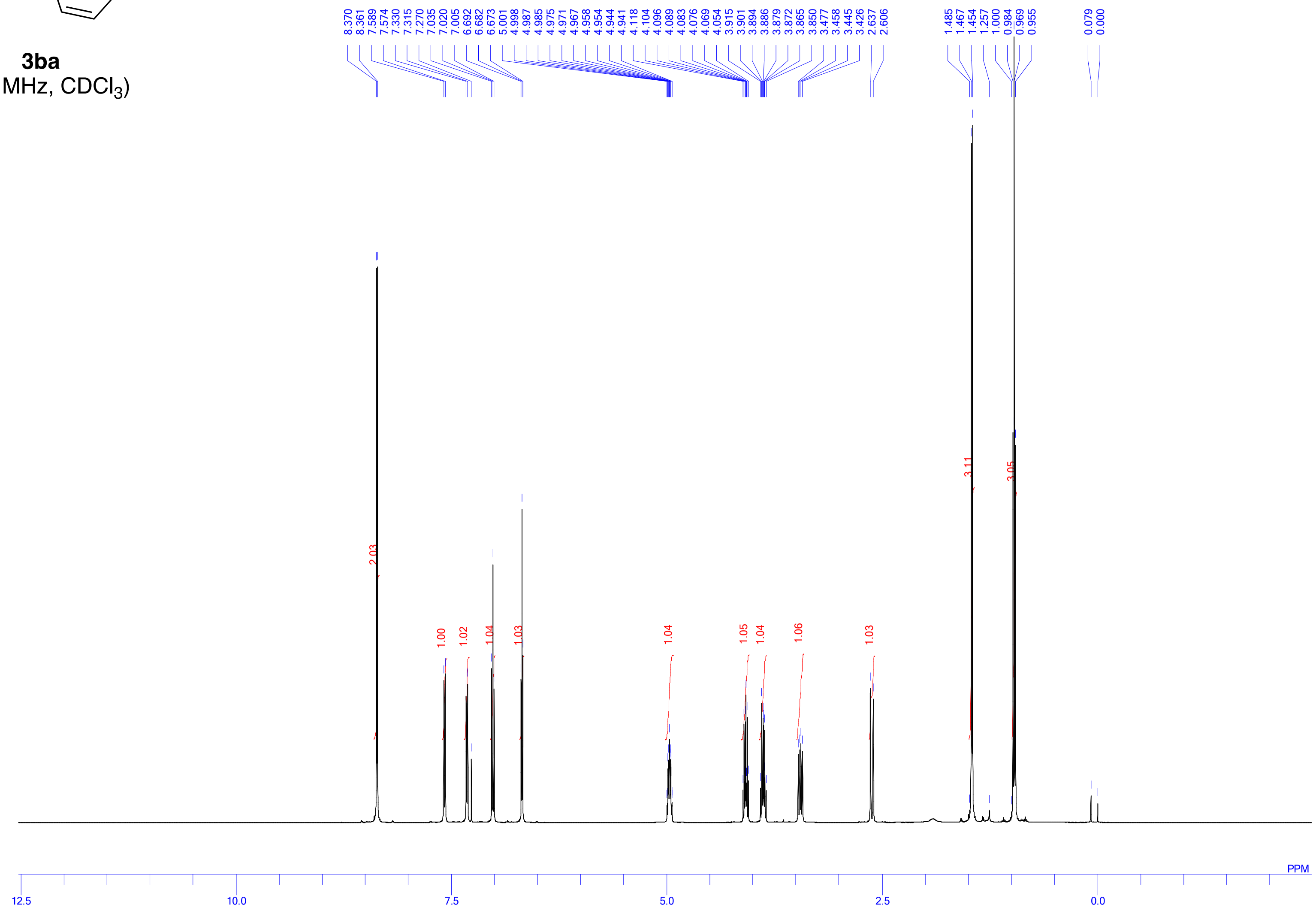

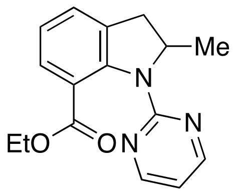

3ba
(125 MHz, $CDCl_3$)

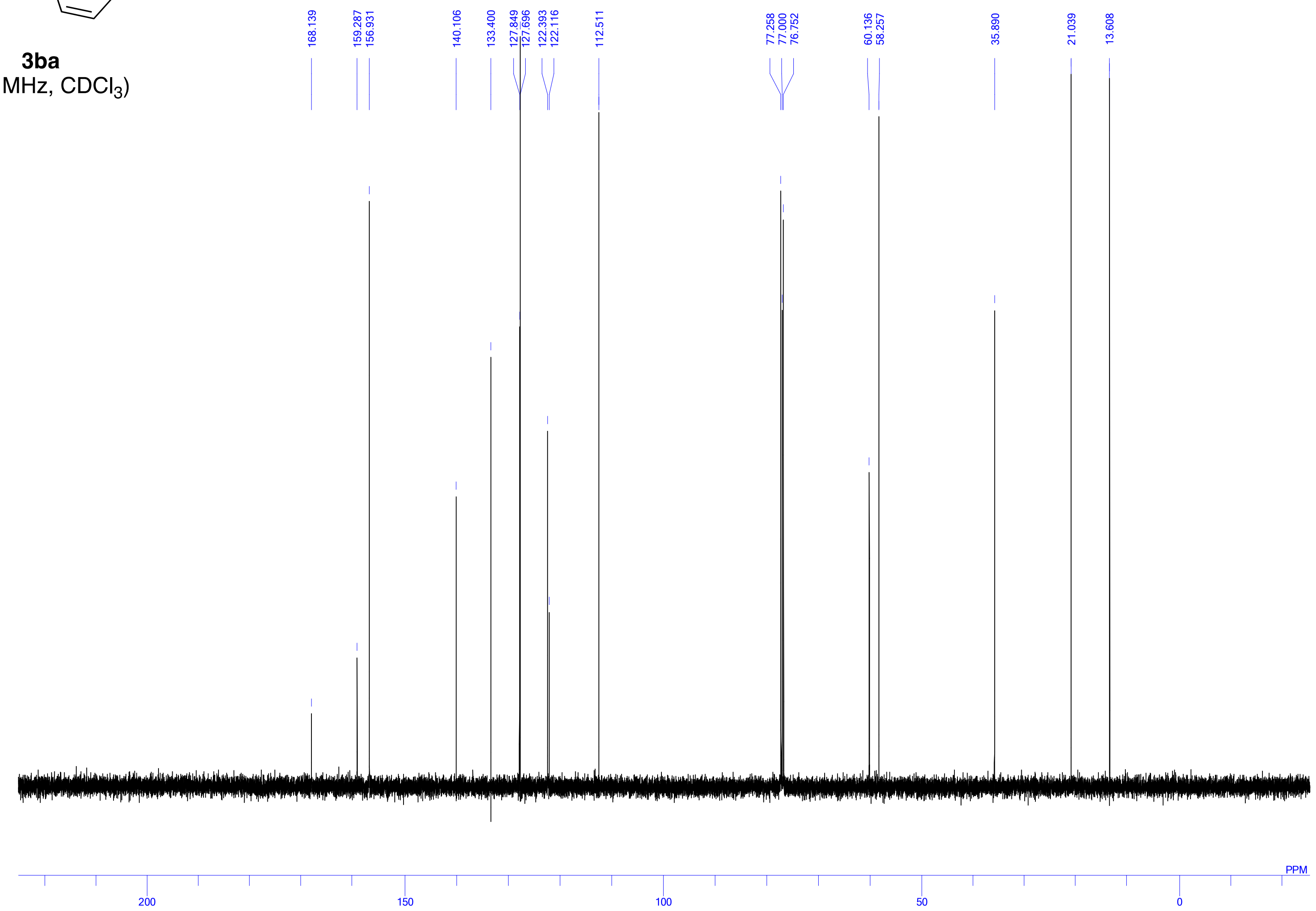

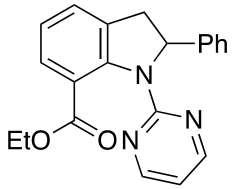

3ca
(500 MHz, $CDCl_3$)

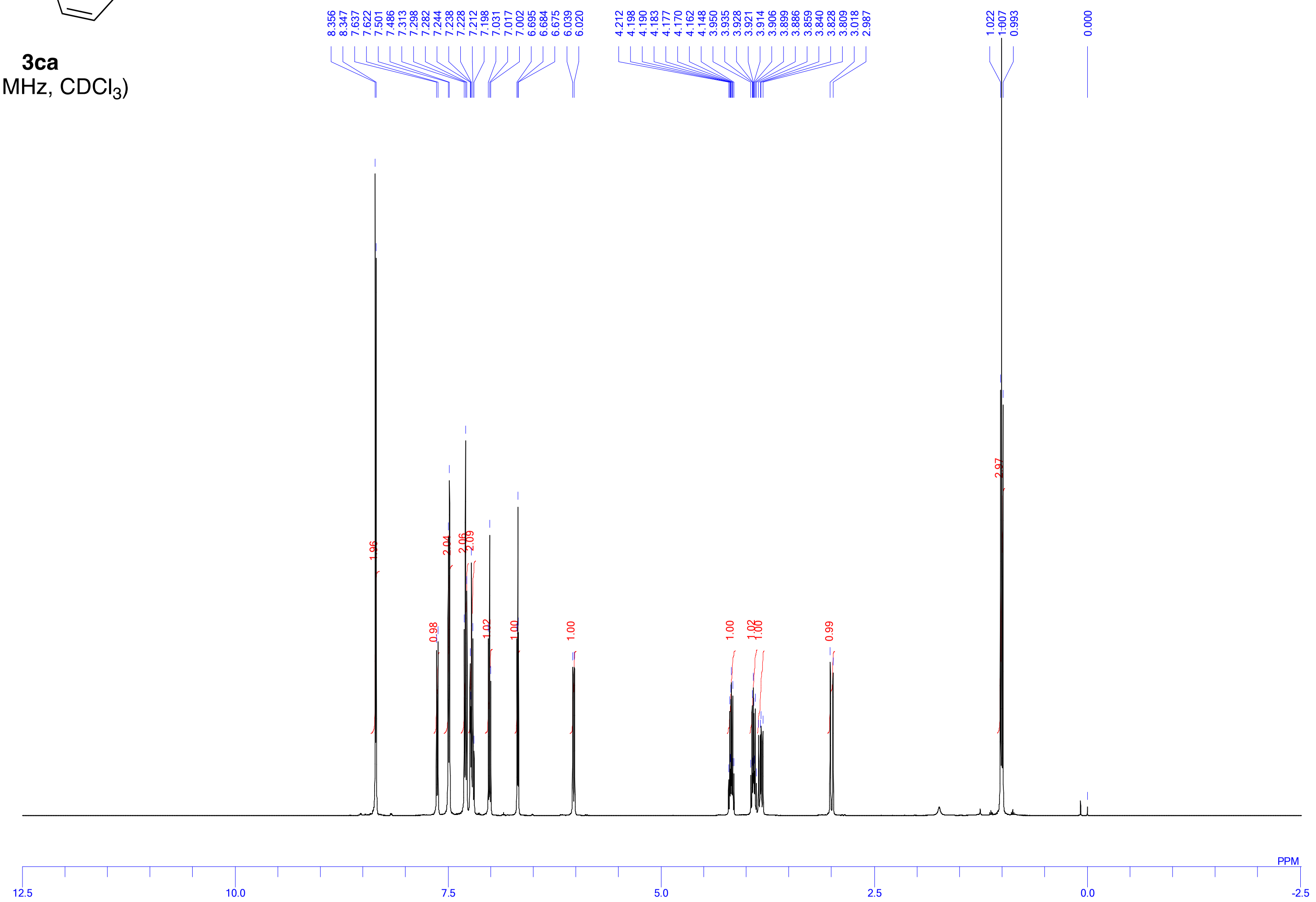

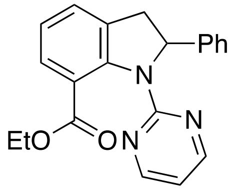

3ca
(125 MHz, $CDCl_3$)

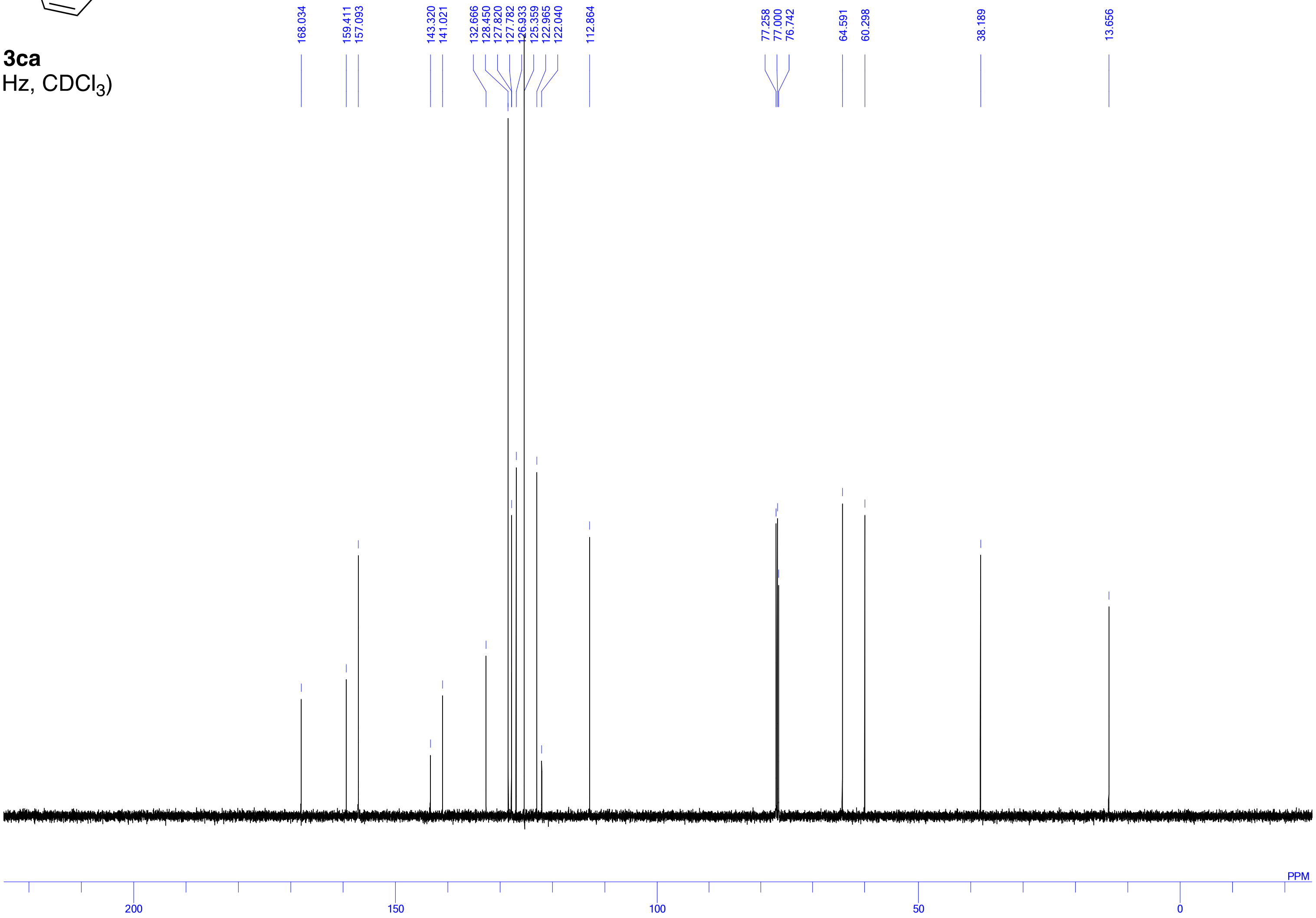

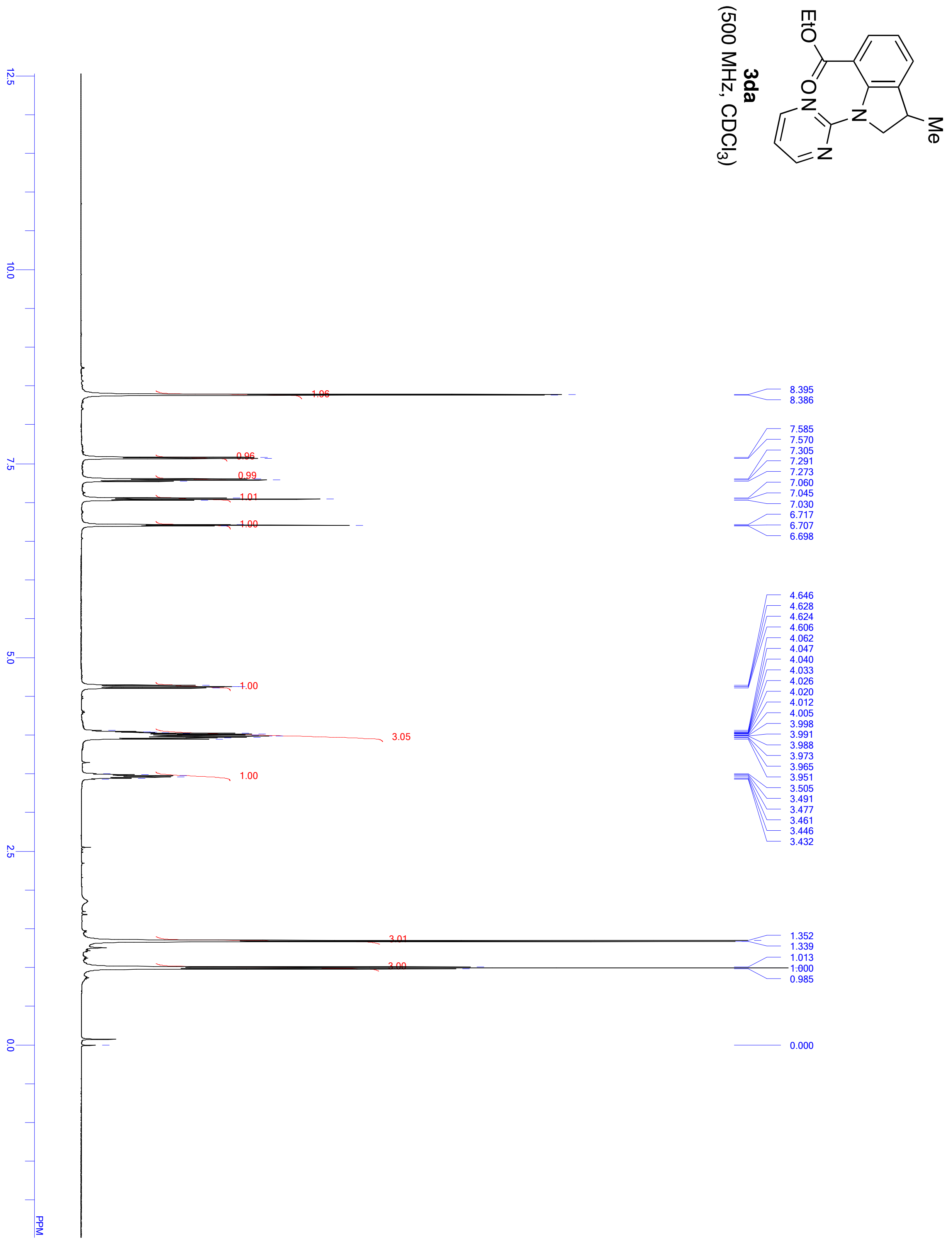
EtO
O
N
N
N
Me
3da
(500 MHz, CDCl3)
8.395
8.386
7.585
7.570
7.305
7.291
7.273
7.060
7.045
7.030
6.717
6.707
6.698
4.646
4.628
4.624
4.606
4.062
4.047
4.040
4.033
4.026
4.020
4.012
4.005
3.998
3.991
3.988
3.973
3.965
3.951
3.505
3.491
3.477
3.461
3.446
3.432
1.352
1.339
1.013
1.000
0.985
0.000
1.06
0.96
0.99
1.01
1.00
1.00
3.05
1.00
3.01
3.00
12.5
10.0
7.5
5.0
2.5
0.0
PPM

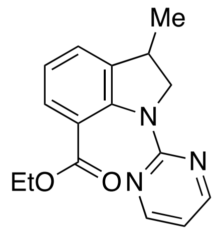

3da
(125 MHz, $CDCl_3$)

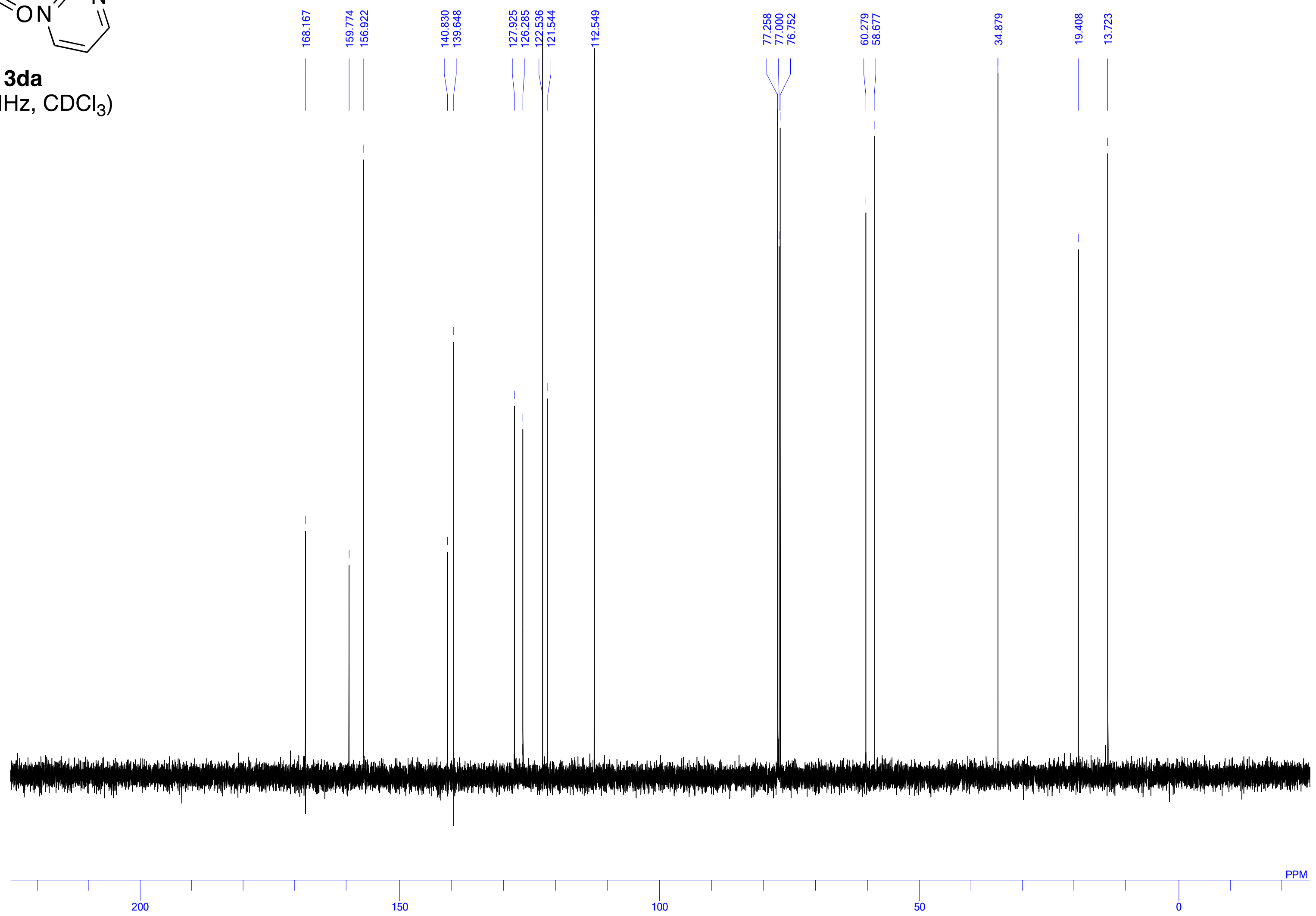

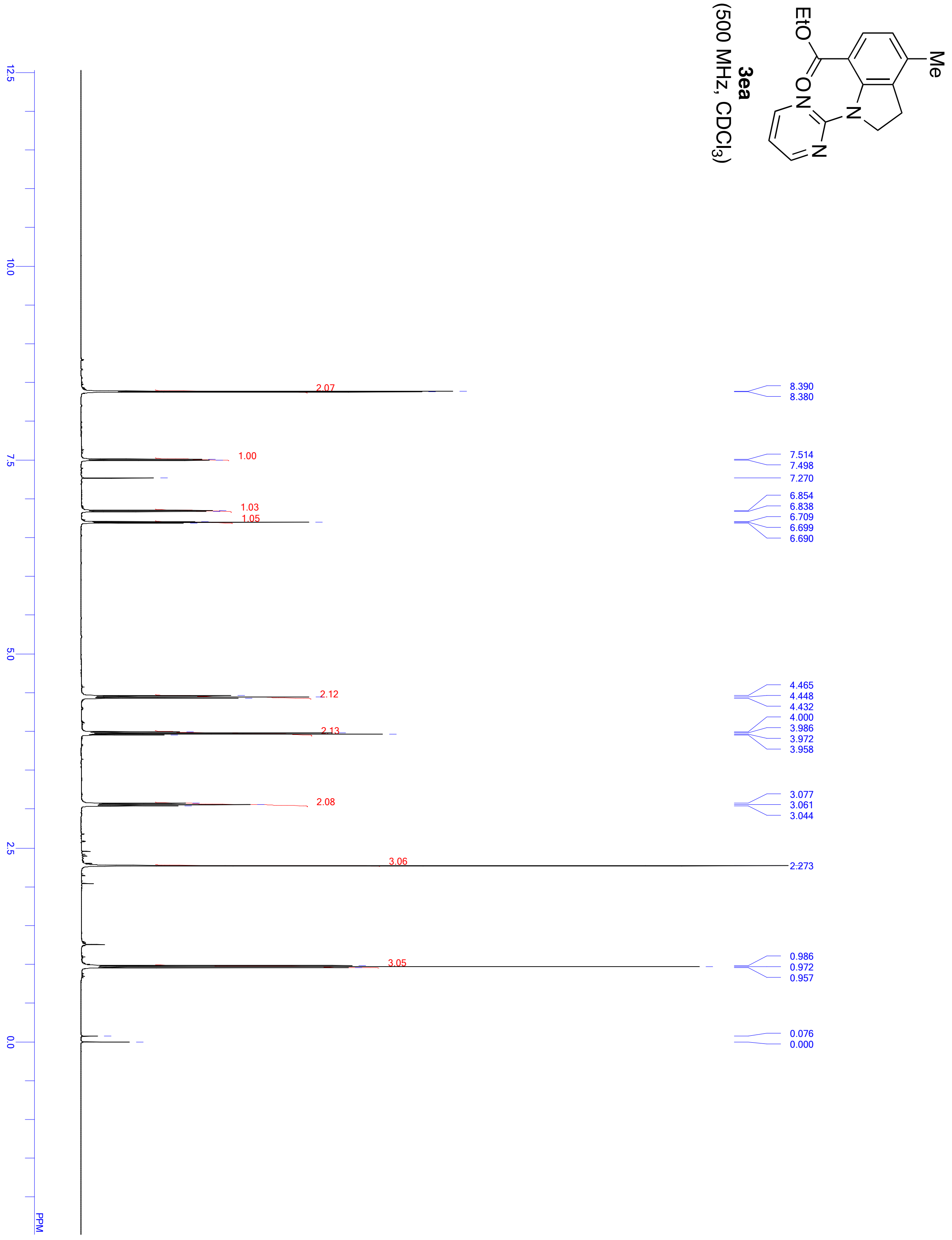

3ea
(500 MHz, $CDCl_3$)

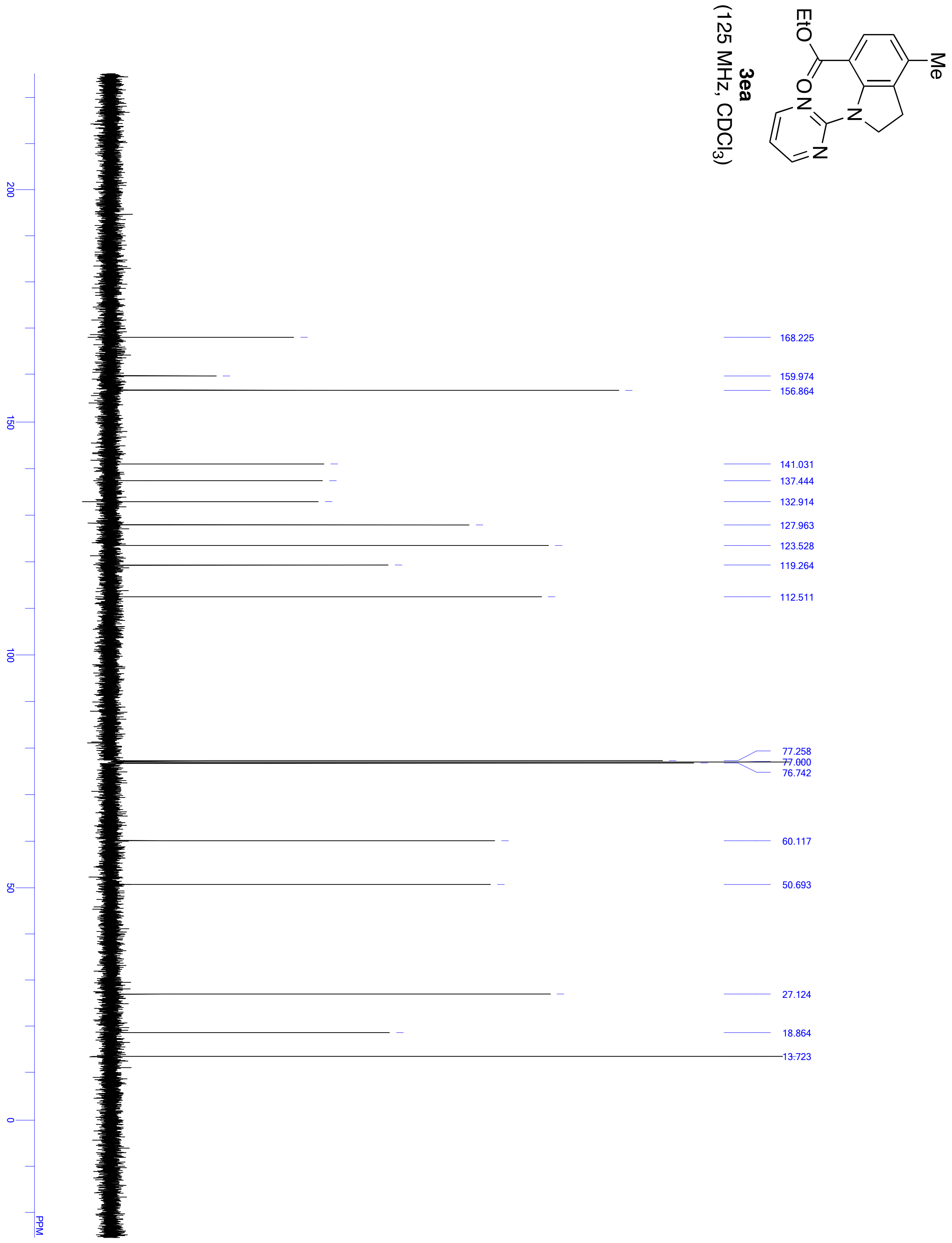
Me
EtO
O
N
N
N
3ea
(125 MHz, CDCl3)
168.225
159.974
156.864
141.031
137.444
132.914
127.963
123.528
119.264
112.511
77.258
77.000
76.742
60.117
50.693
27.124
18.864
13.723
200
150
100
50
0
PPM

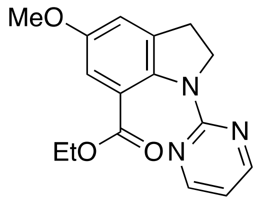

3fa
(500 MHz, $CDCl_3$)

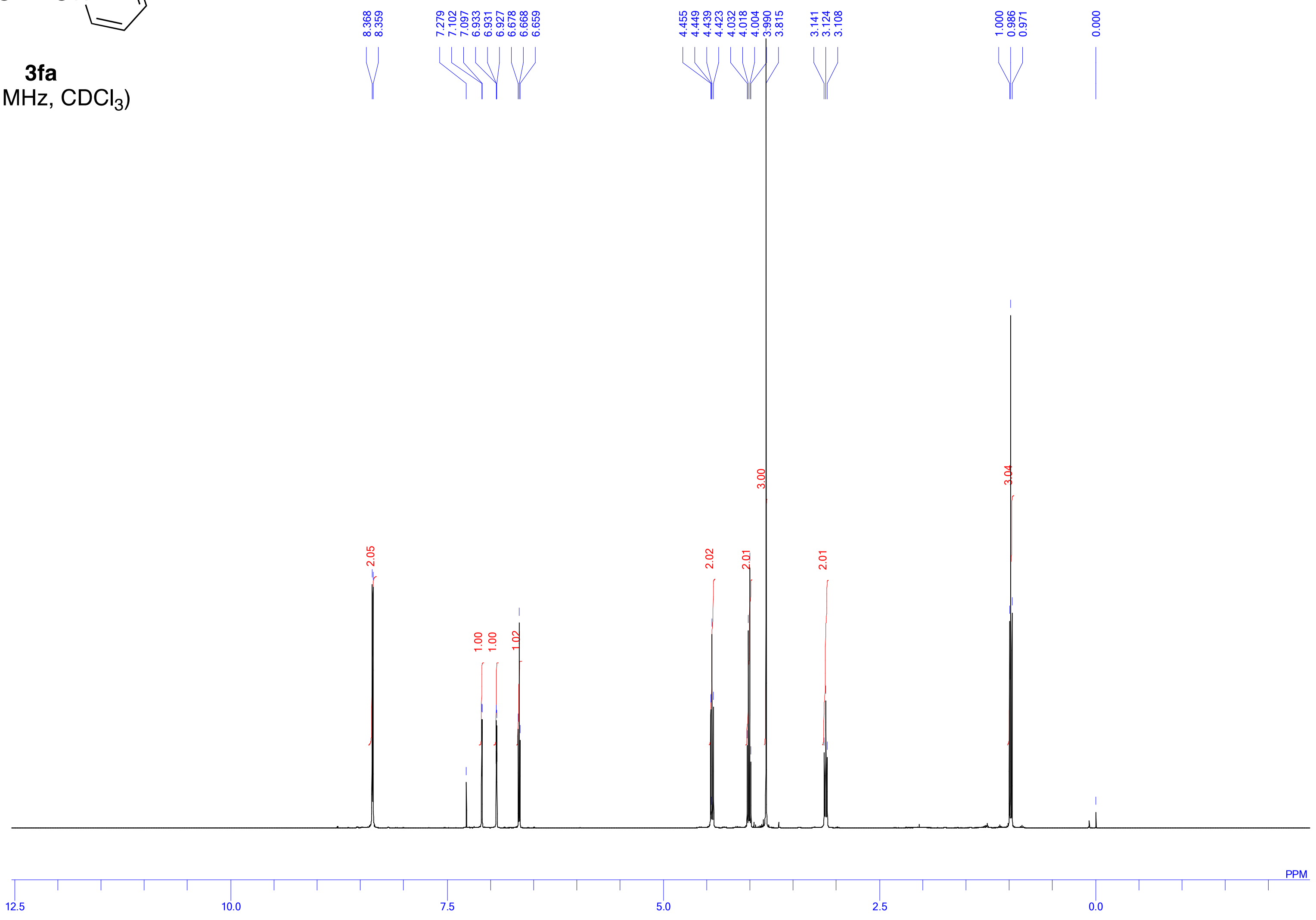

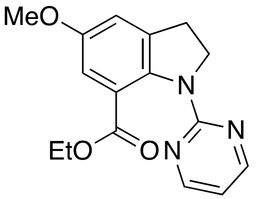

3fa
(125 MHz, $CDCl_3$)

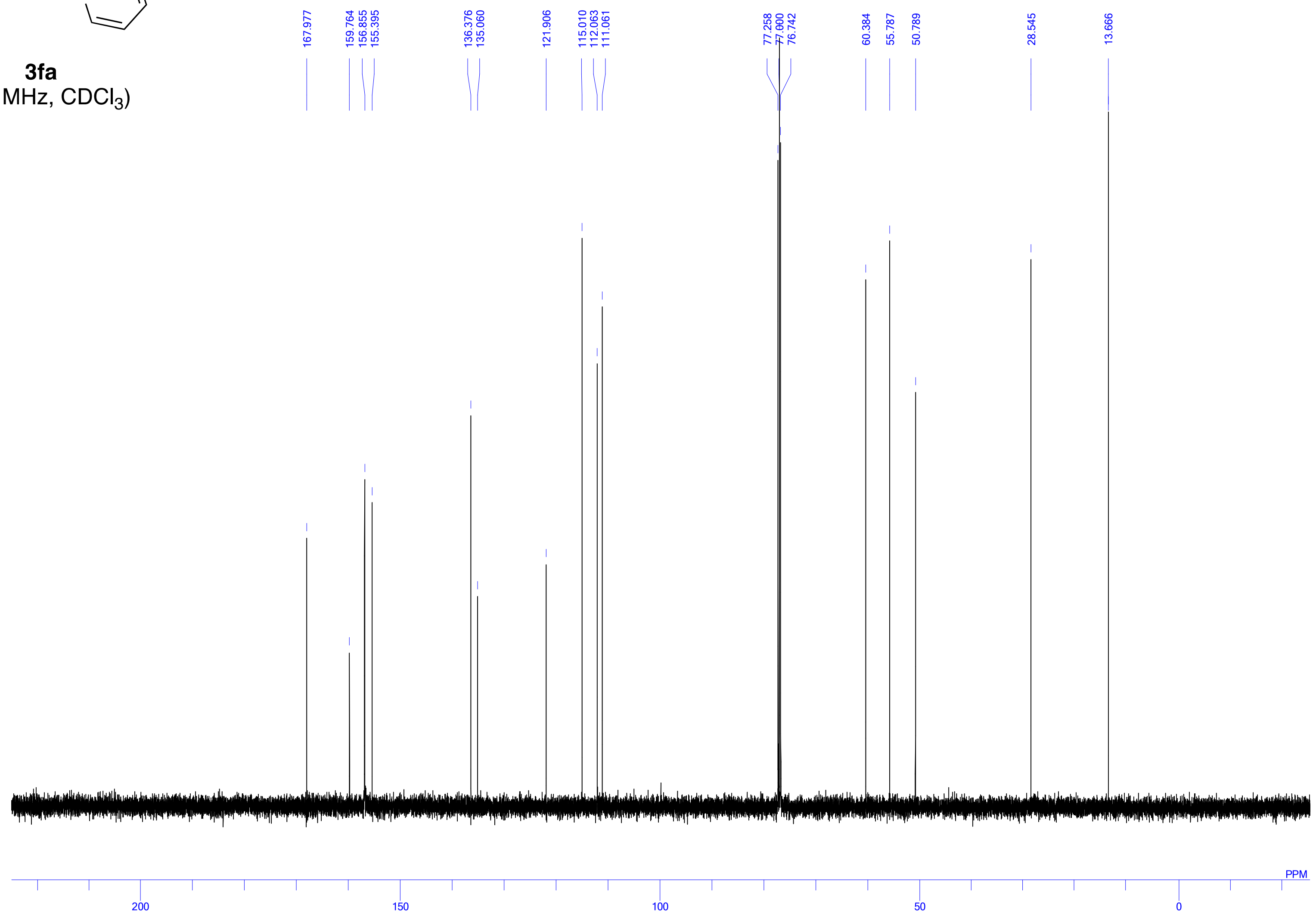

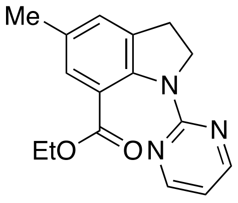

3ga
(500 MHz, $CDCl_3$)

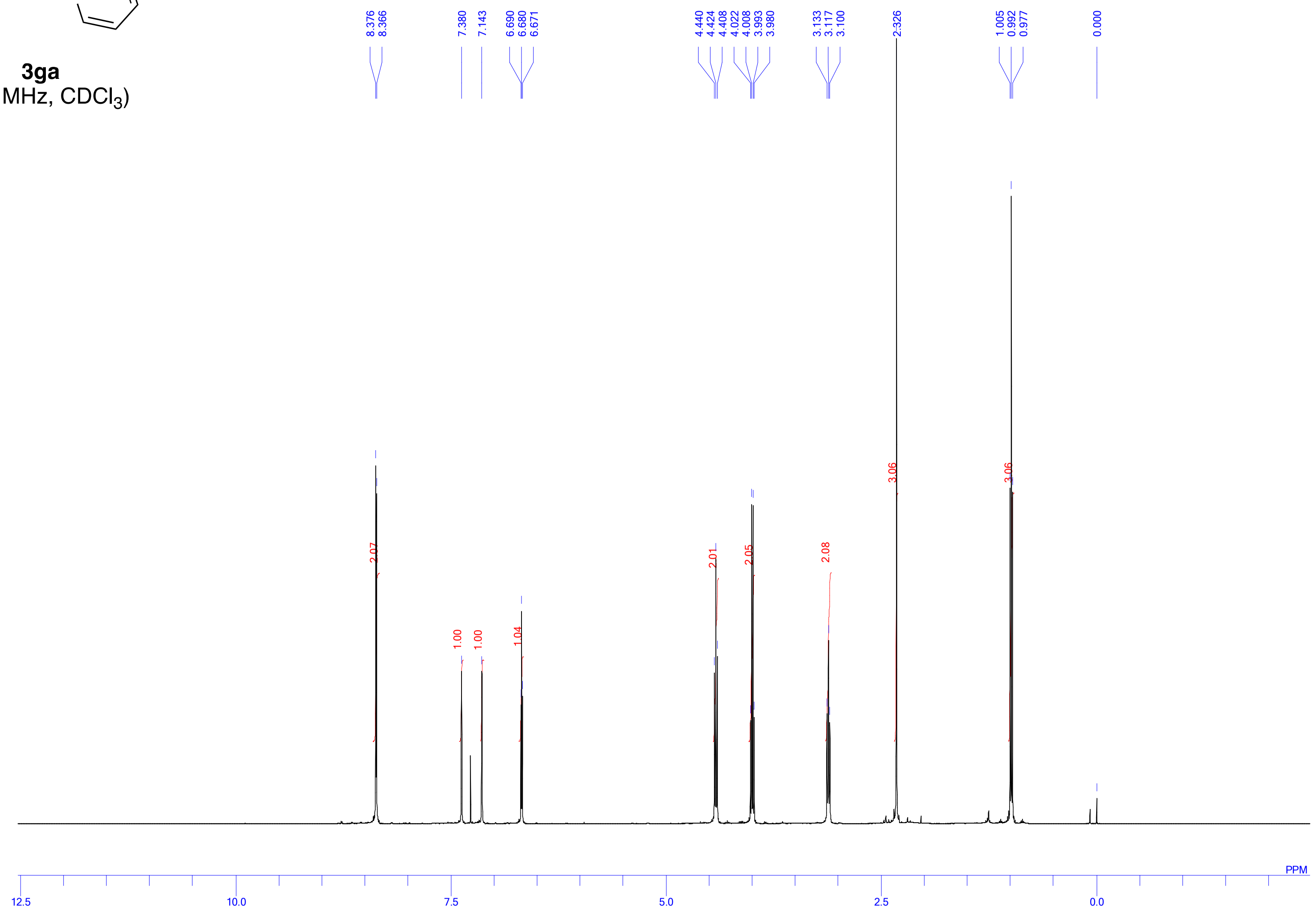

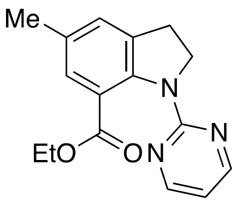

3ga
(125 MHz, $CDCl_3$)

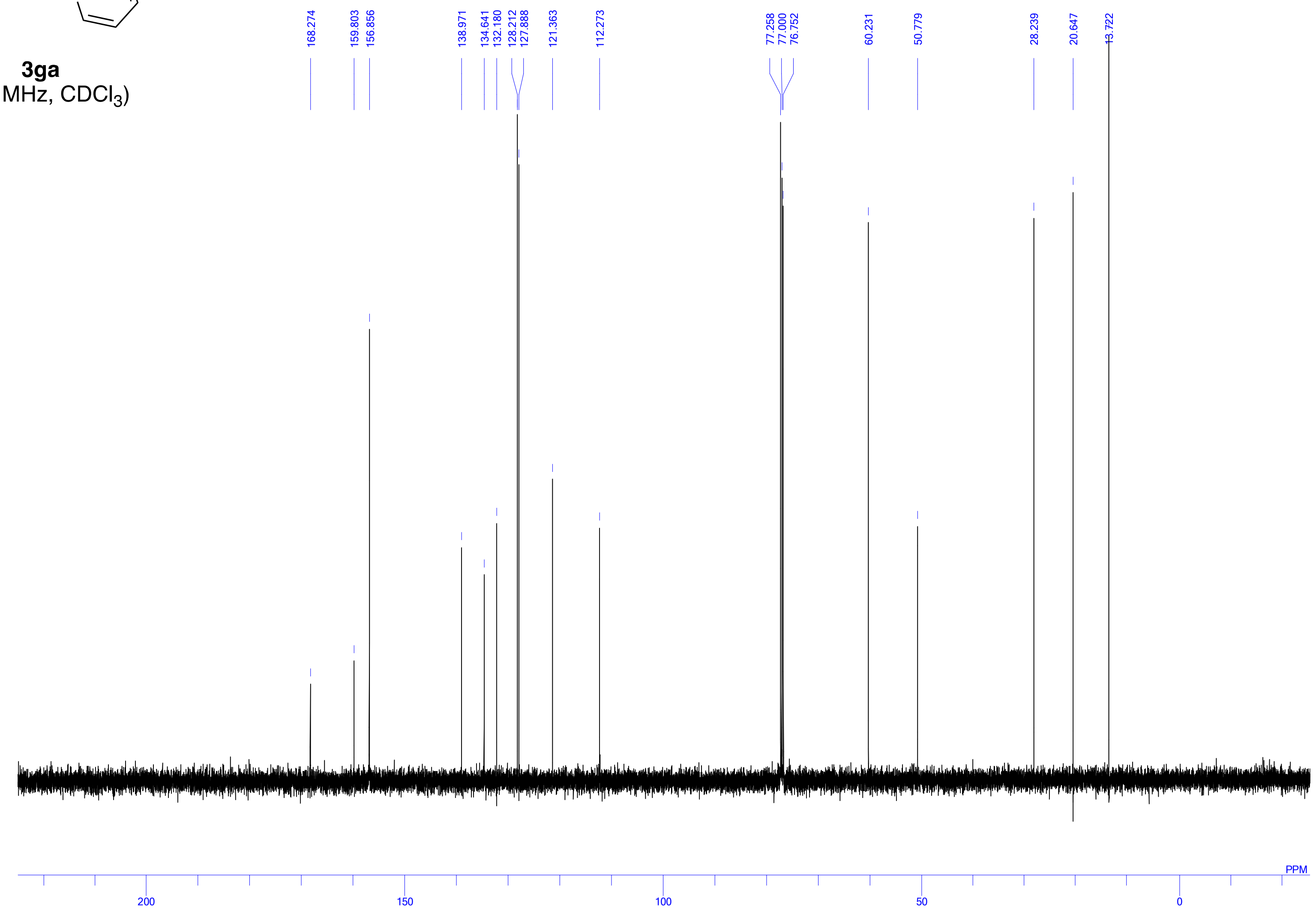

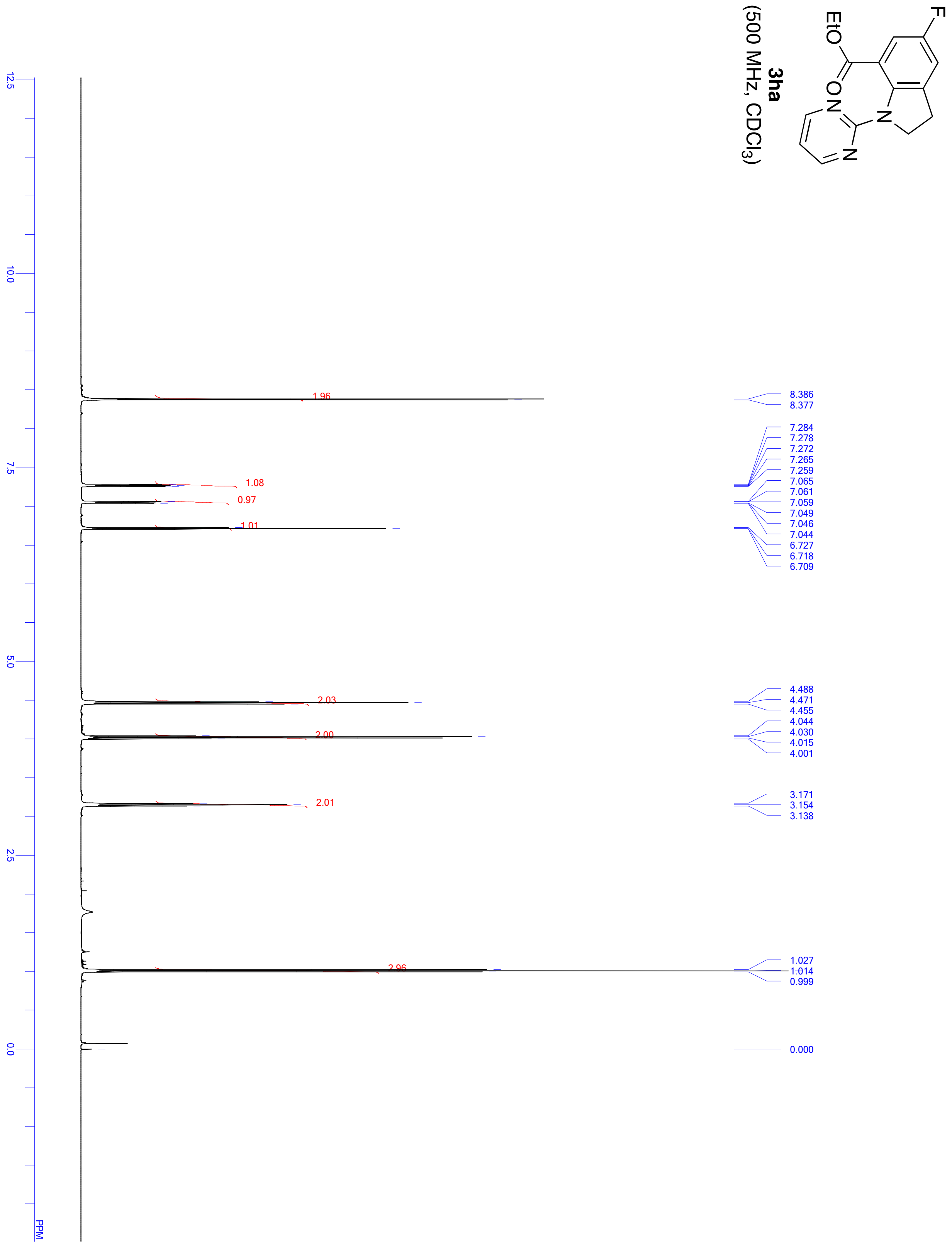

F
EtO
O
N
N
N
3ha
(500 MHz, CDCl3)
8.386
8.377
7.284
7.278
7.272
7.265
7.259
7.065
7.061
7.059
7.049
7.046
7.044
6.727
6.718
6.709
4.488
4.471
4.455
4.044
4.030
4.015
4.001
3.171
3.154
3.138
1.027
1.014
0.999
0.000
1.96
1.08
0.97
1.01
2.03
2.00
2.01
2.96
12.5
10.0
7.5
5.0
2.5
0.0
PPM

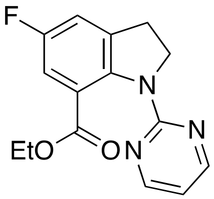

3ha
(125 MHz, $CDCl_3$)

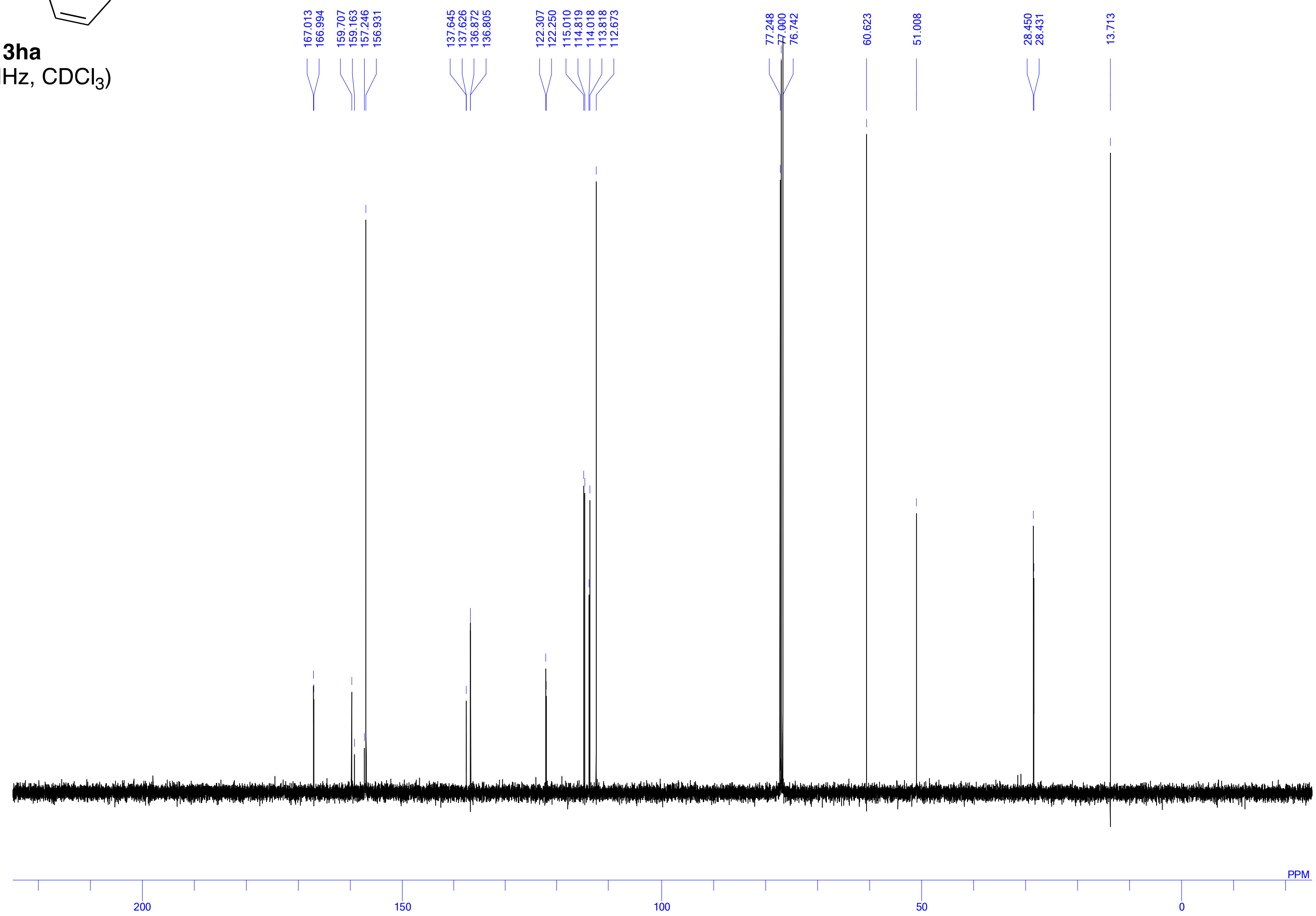

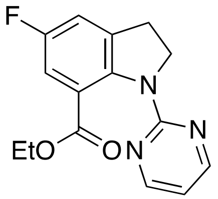

3ha
(470 MHz, $CDCl_3$)

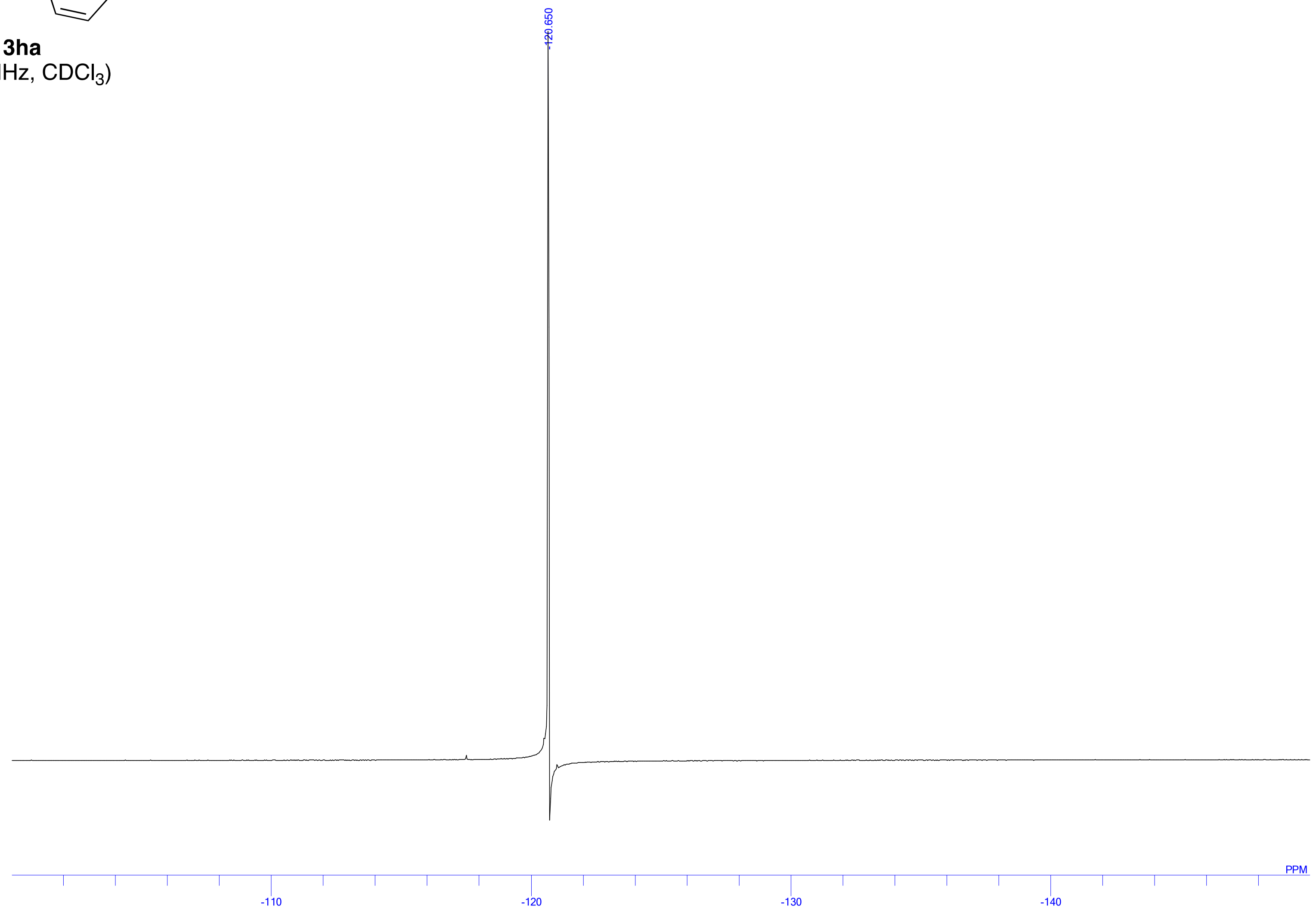

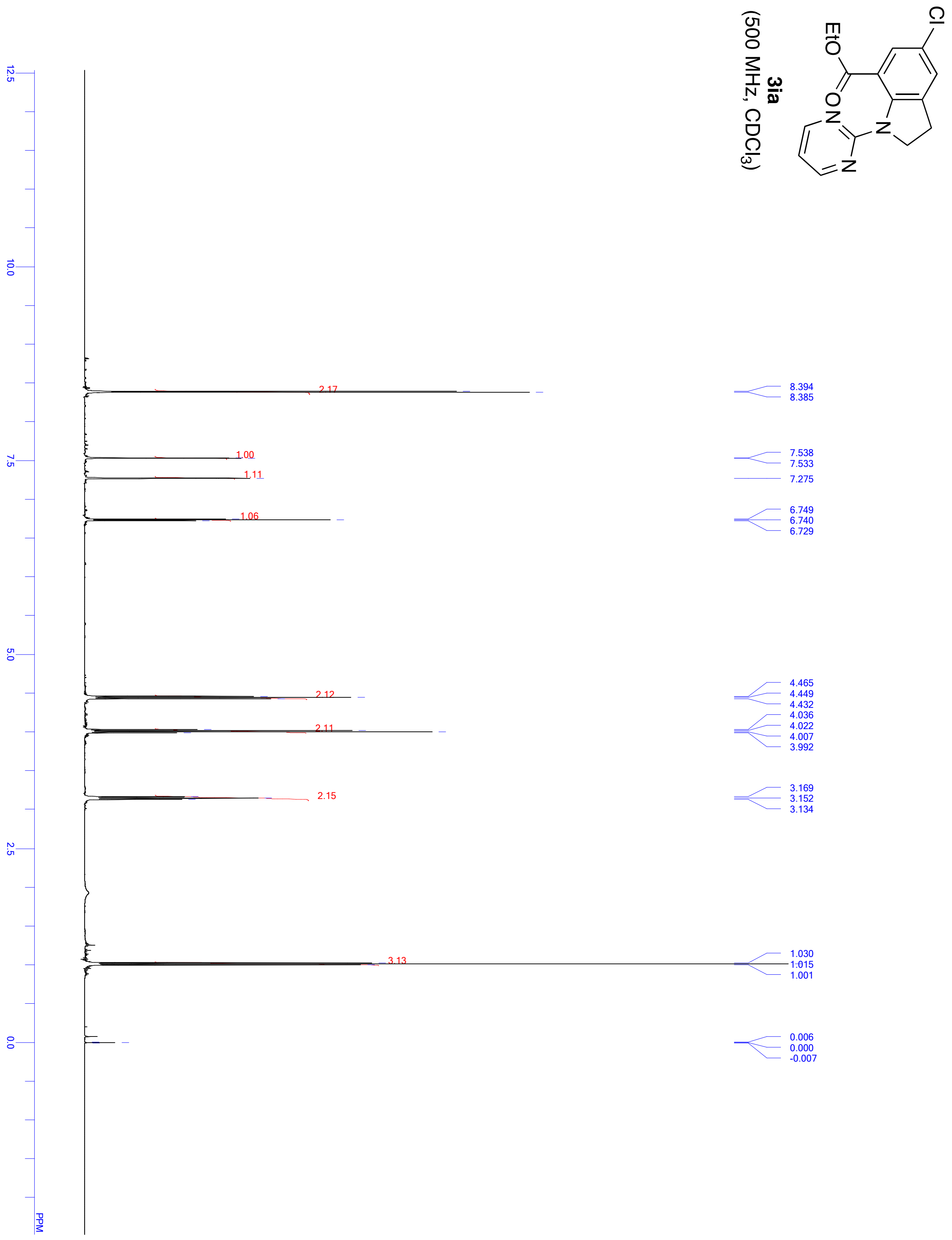
Cl
EtO
O
N
N
N
3ia
(500 MHz, CDCl3)
8.394
8.385
7.538
7.533
7.275
6.749
6.740
6.729
4.465
4.449
4.432
4.036
4.022
4.007
3.992
3.169
3.152
3.134
1.030
1.015
1.001
0.006
0.000
-0.007
2.17
1.00
1.11
1.06
2.12
2.11
2.15
3.13
12.5
10.0
7.5
5.0
2.5
0.0
PPM

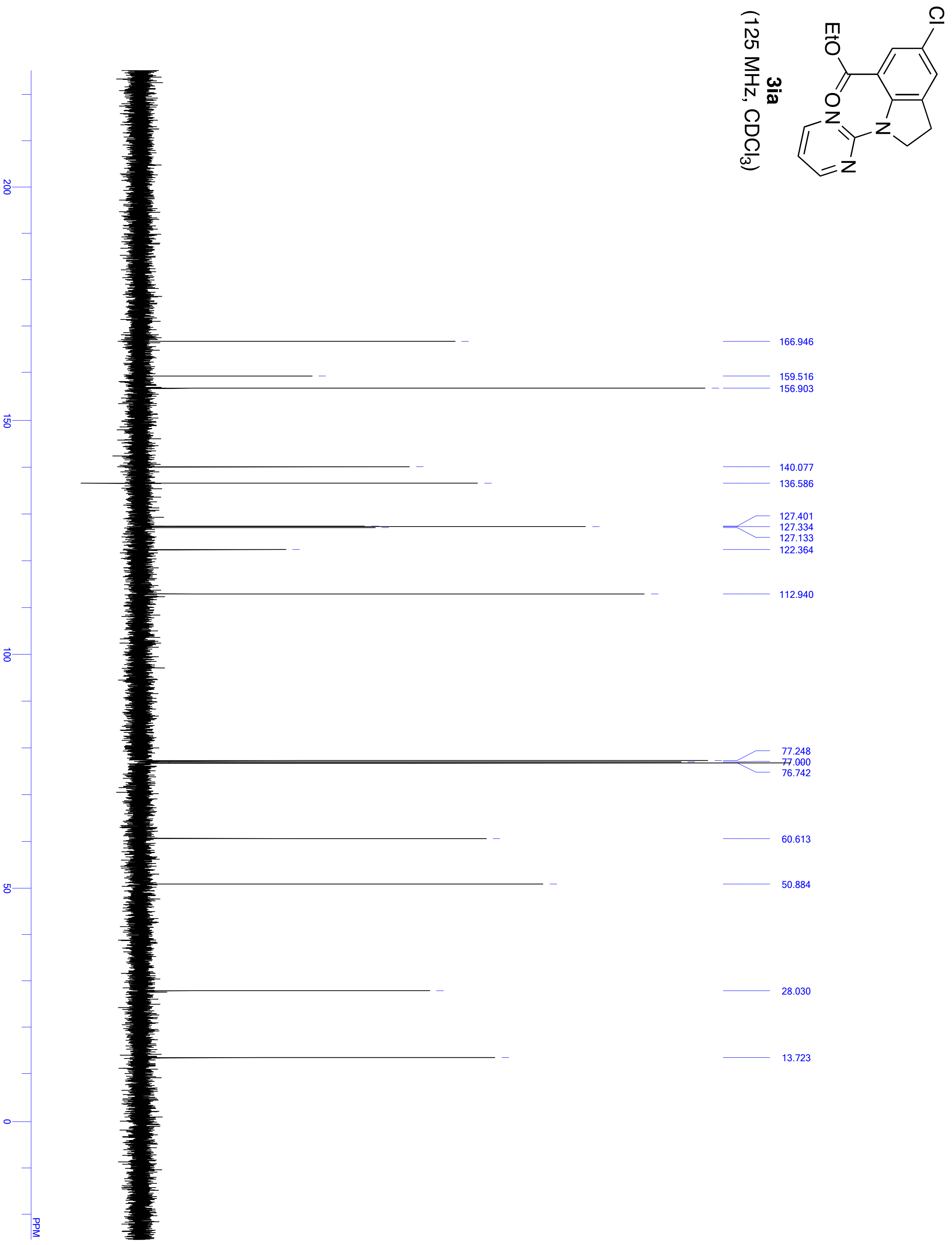

Cl
EtO
O
N
N
N
3ia
(125 MHz, CDCl3)
166.946
159.516
156.903
140.077
136.586
127.401
127.334
127.133
122.364
112.940
77.248
77.000
76.742
60.613
50.884
28.030
13.723
200
150
100
50
0
PPM

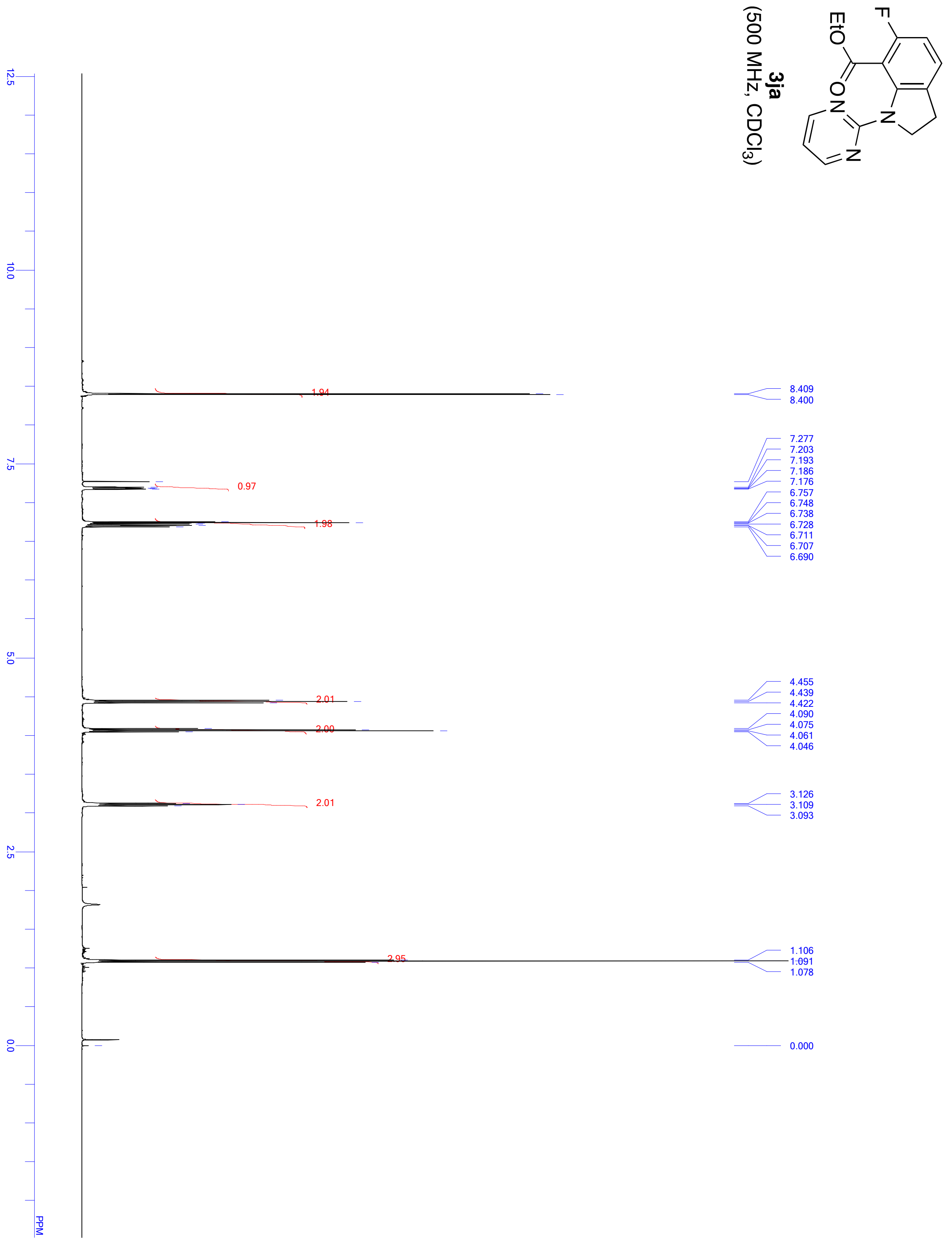
3ja
(500 MHz, CDCl3)
EtO
F
O
N
N
N
12.5
10.0
7.5
5.0
2.5
0.0
PPM
1.94
0.97
1.98
2.01
2.00
2.01
2.95
8.409
8.400
7.277
7.203
7.193
7.186
7.176
6.757
6.748
6.738
6.728
6.711
6.707
6.690
4.455
4.439
4.422
4.090
4.075
4.061
4.046
3.126
3.109
3.093
1.106
1.091
1.078
0.000

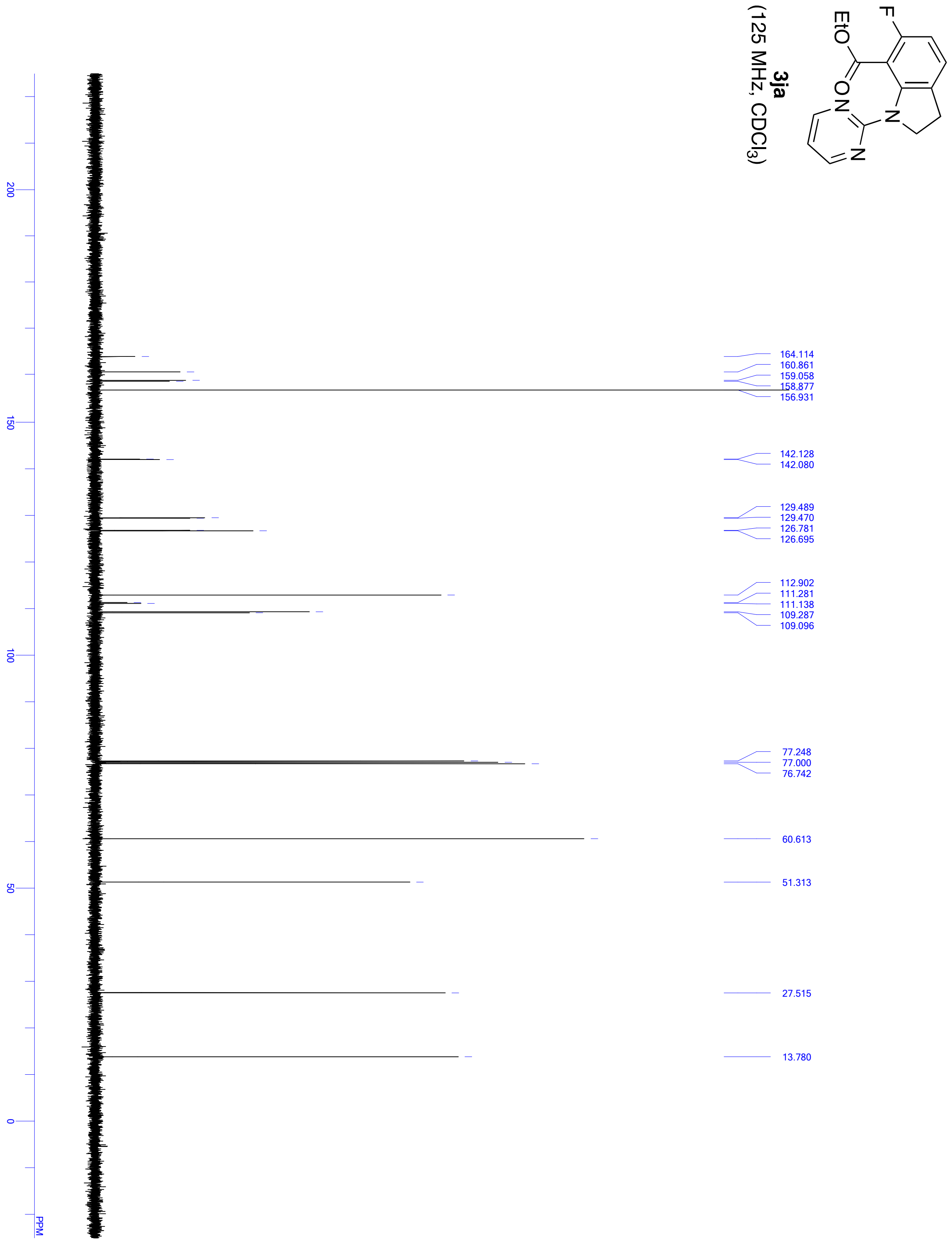
F
EtO
O
N
N
N
3ja
(125 MHz, CDCl3)
164.114
160.861
159.058
158.877
156.931
142.128
142.080
129.489
129.470
126.781
126.695
112.902
111.281
111.138
109.287
109.096
77.248
77.000
76.742
60.613
51.313
27.515
13.780
200
150
100
50
0
PPM

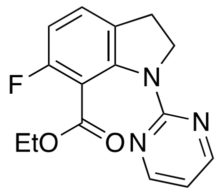

3ja
(470 MHz, $CDCl_3$)

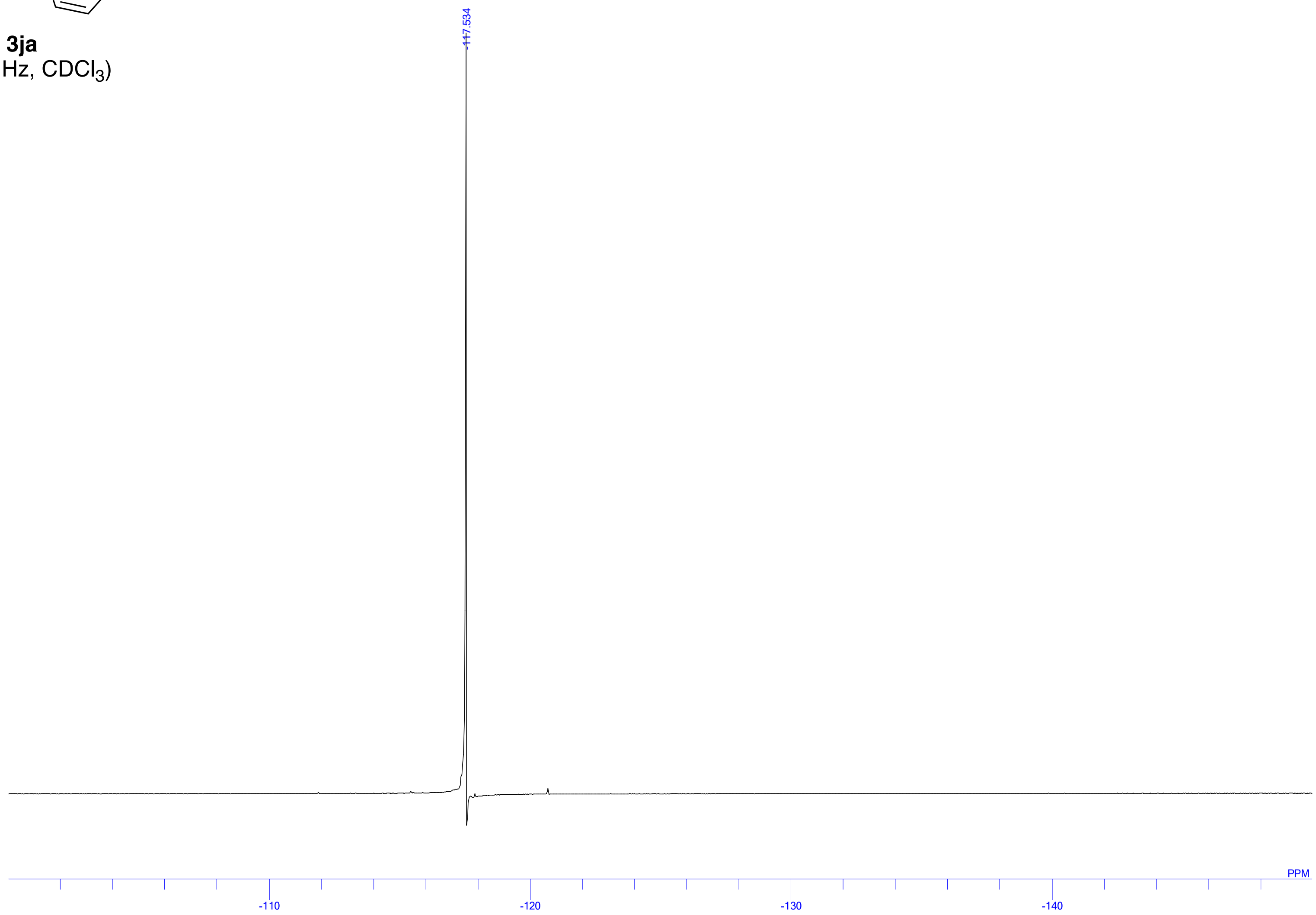

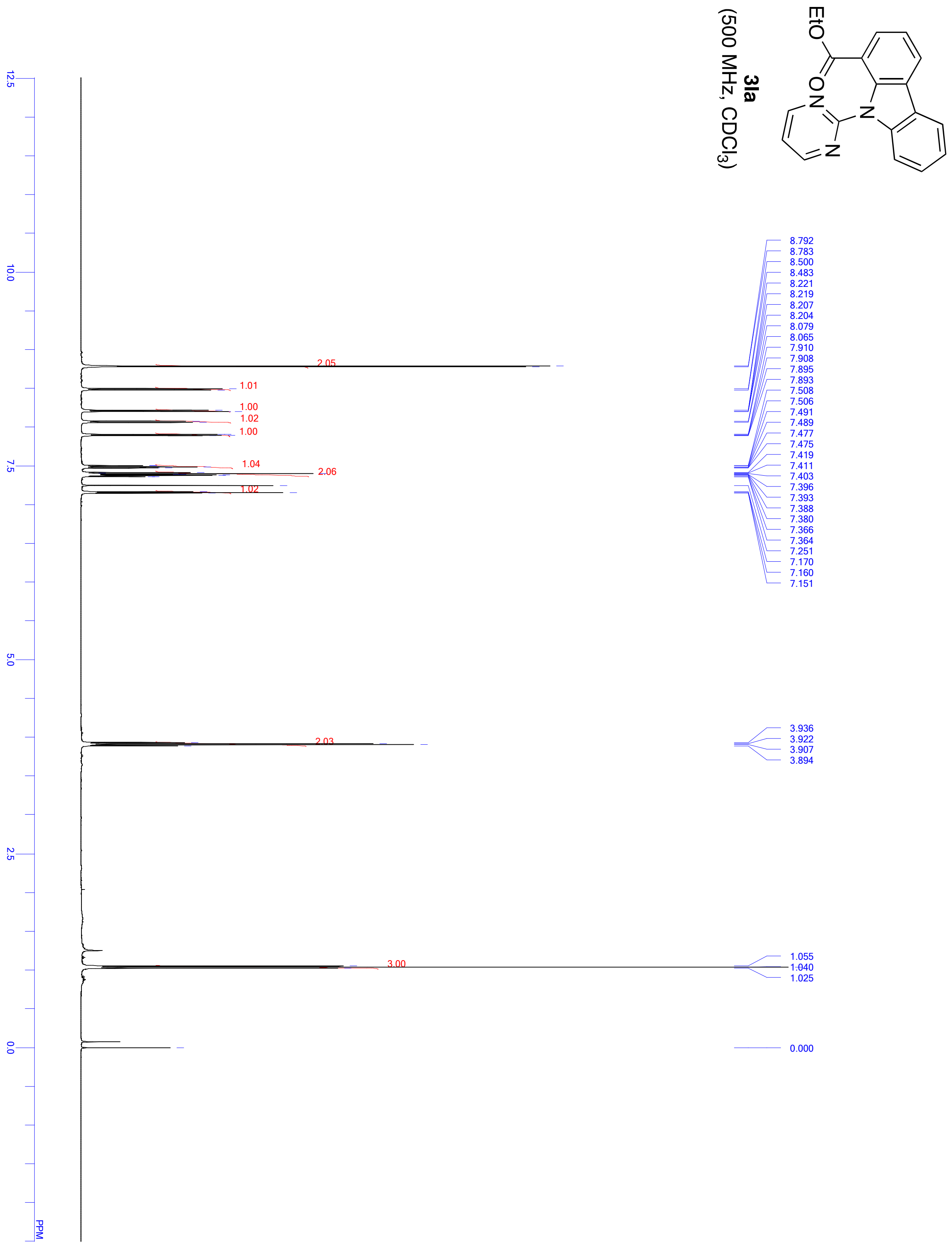
3la
(500 MHz, CDCl3)
8.792
8.783
8.500
8.483
8.221
8.219
8.207
8.204
8.079
8.065
7.910
7.908
7.895
7.893
7.508
7.506
7.491
7.489
7.477
7.475
7.419
7.411
7.403
7.396
7.393
7.388
7.380
7.366
7.364
7.251
7.170
7.160
7.151
3.936
3.922
3.907
3.894
1.055
1.040
1.025
0.000
2.05
1.01
1.00
1.02
1.00
1.04
2.06
1.02
2.03
3.00
12.5
10.0
7.5
5.0
2.5
0.0
PPM

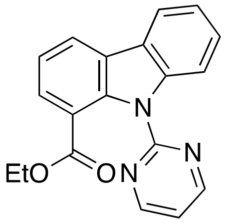

3la
(125 MHz, $CDCl_3$)

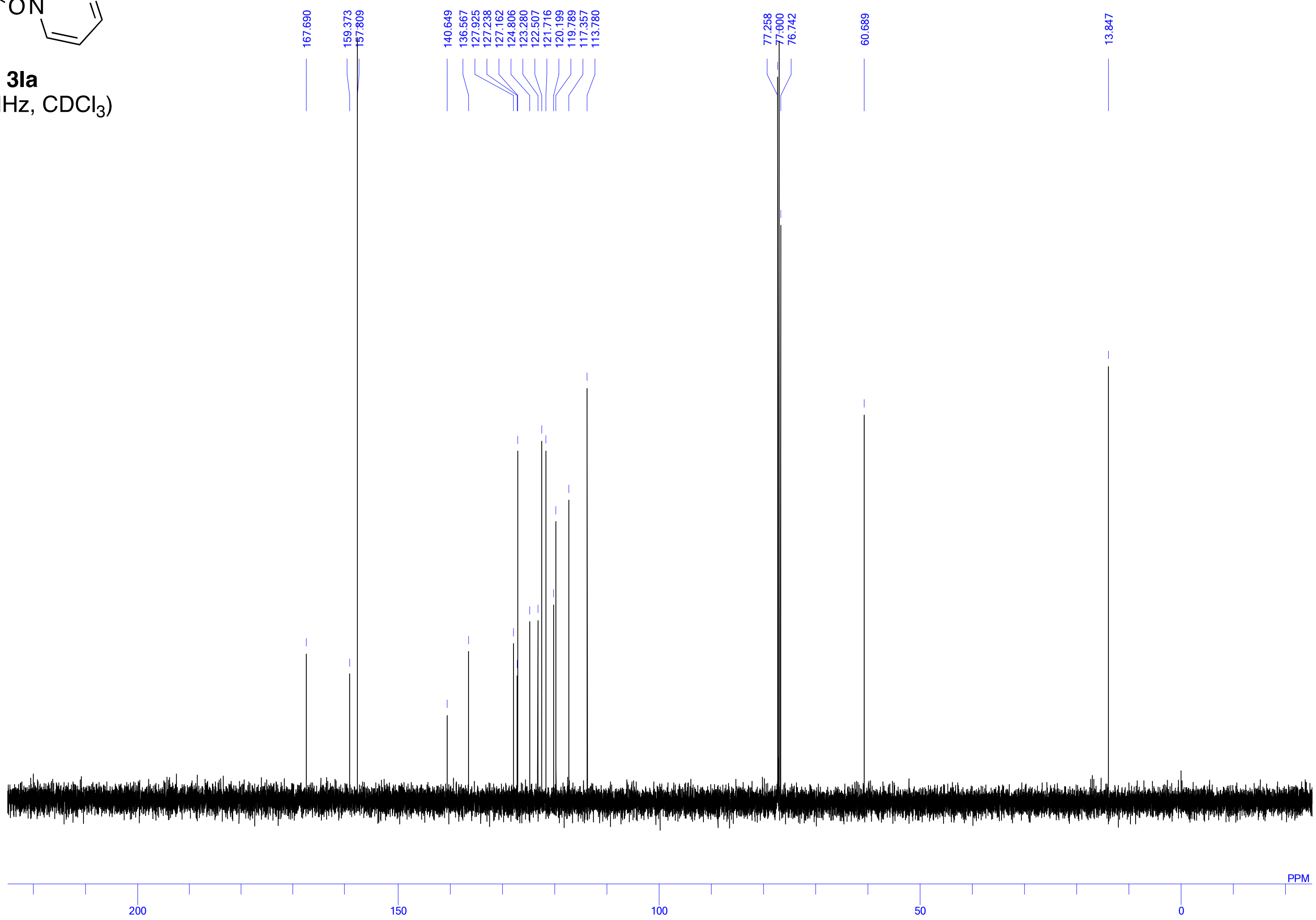

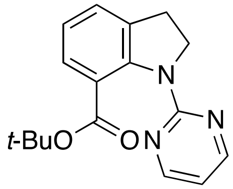

3ab
(500 MHz, $CDCl_3$)

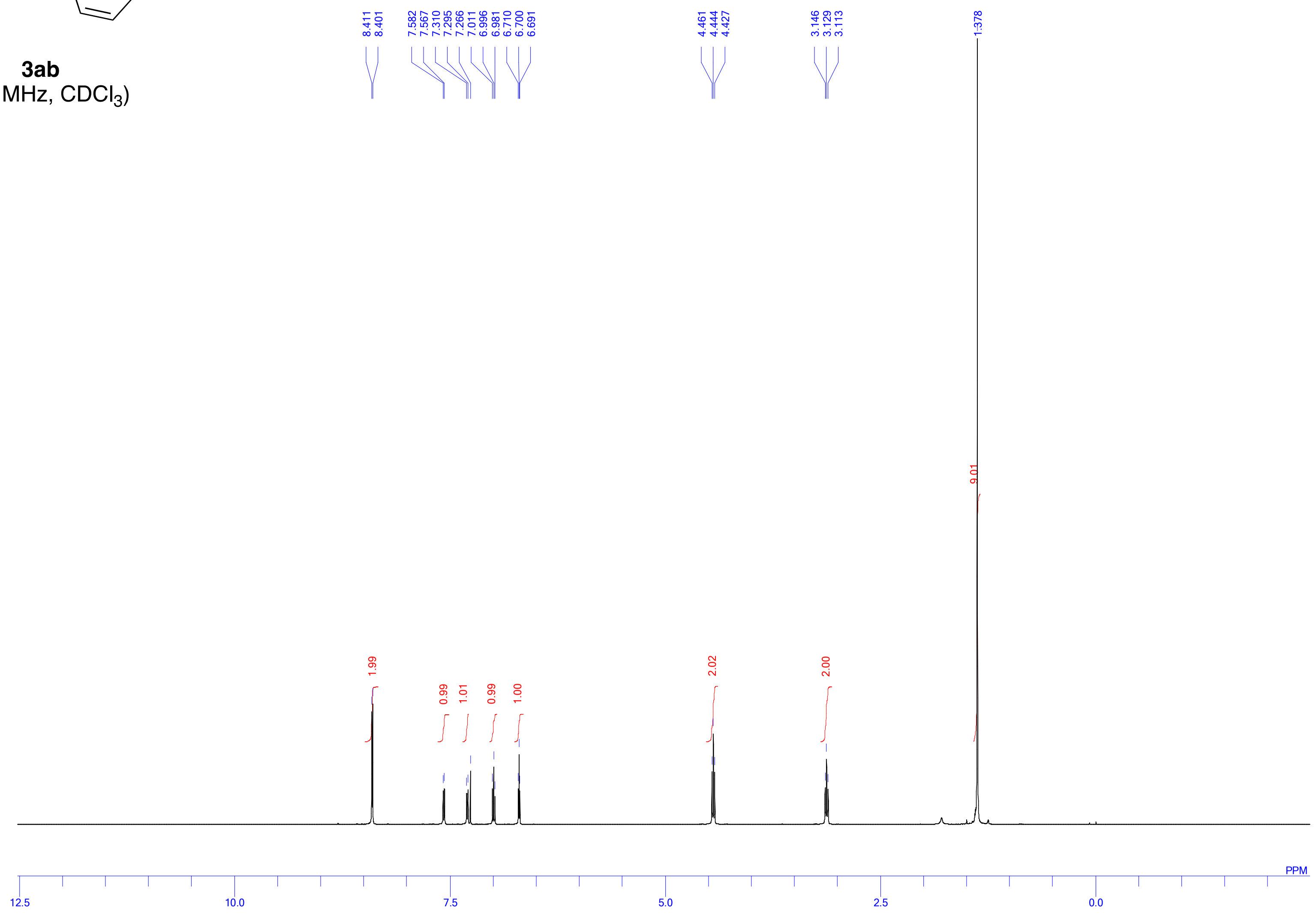

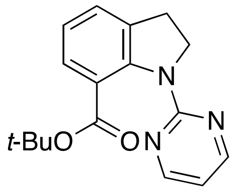

3ab
(125 MHz, $CDCl_3$)

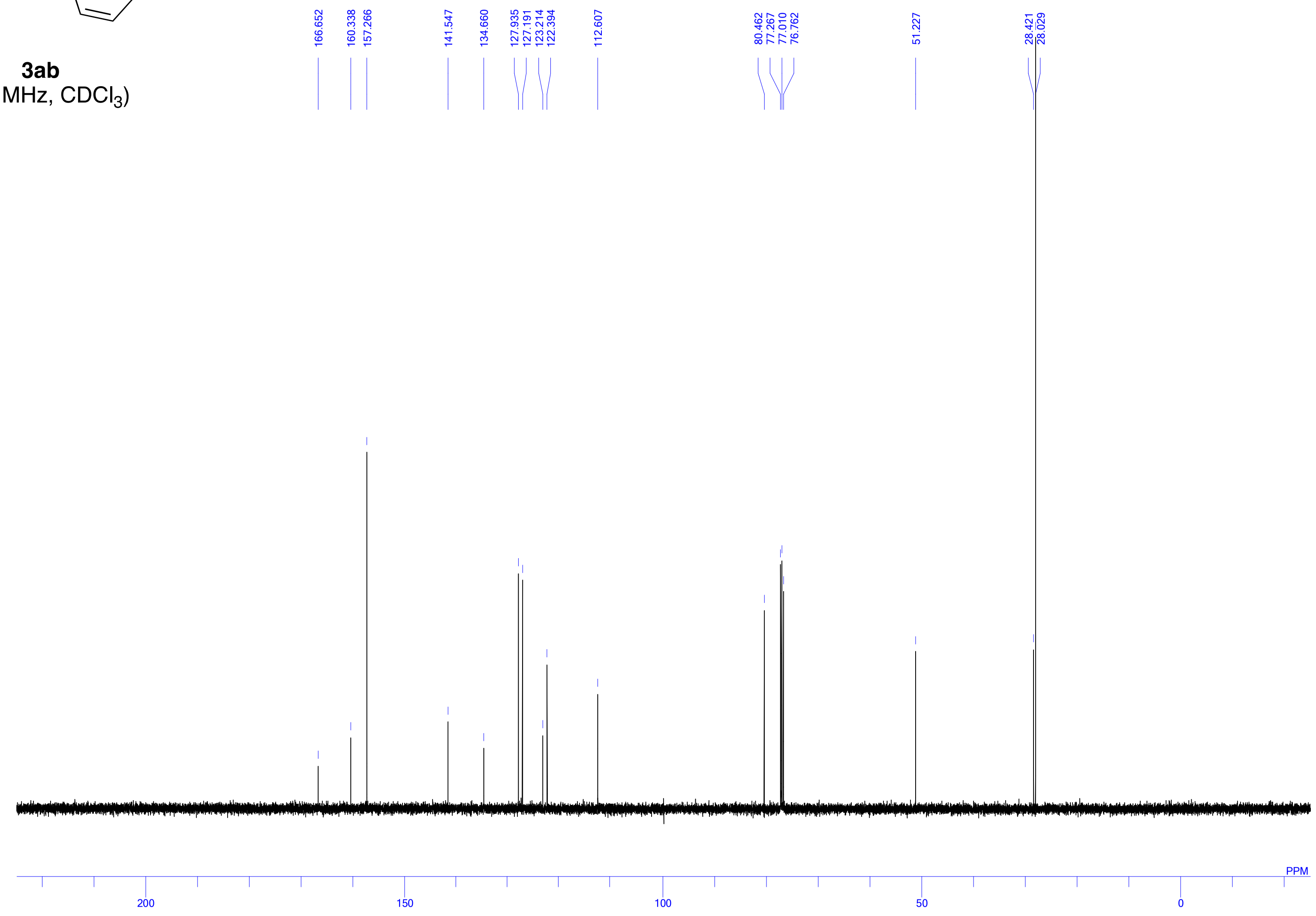

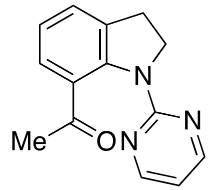

5aa
(500 MHz, $CDCl_3$)

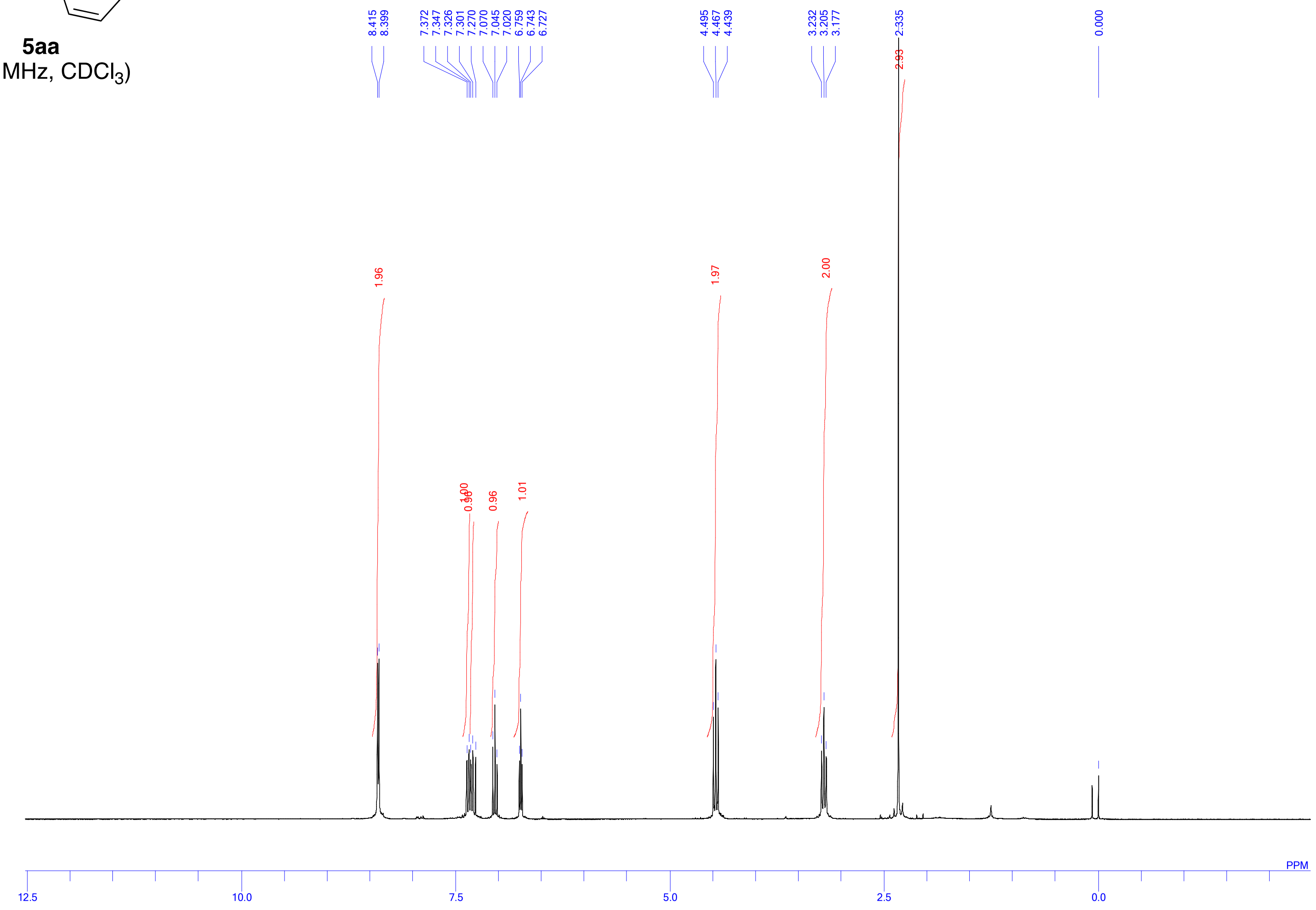

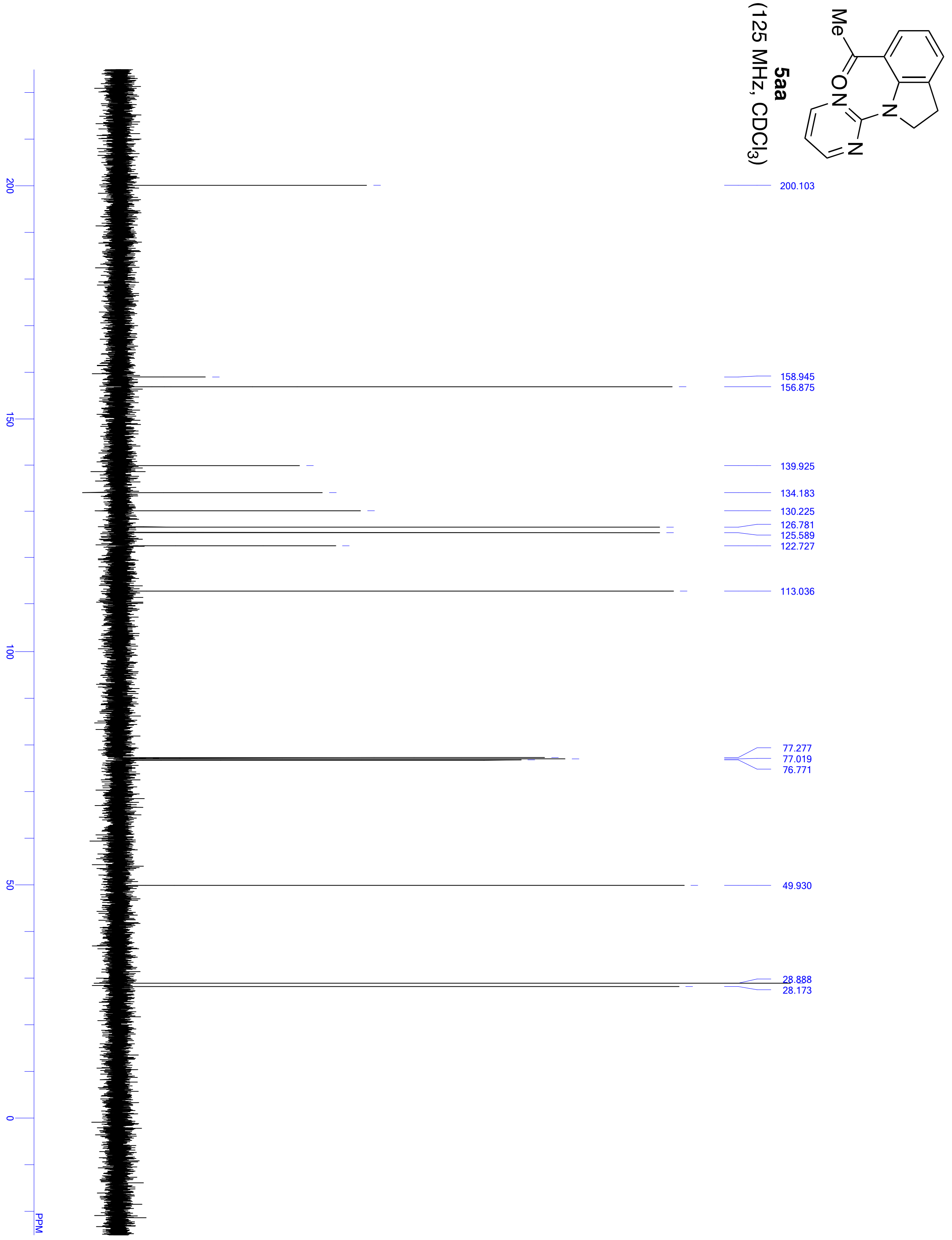
5aa
(125 MHz, CDCl3)
200.103
158.945
156.875
139.925
134.183
130.225
126.781
125.589
122.727
113.036
77.277
77.019
76.771
49.930
28.888
28.173
200
150
100
50
0
PPM

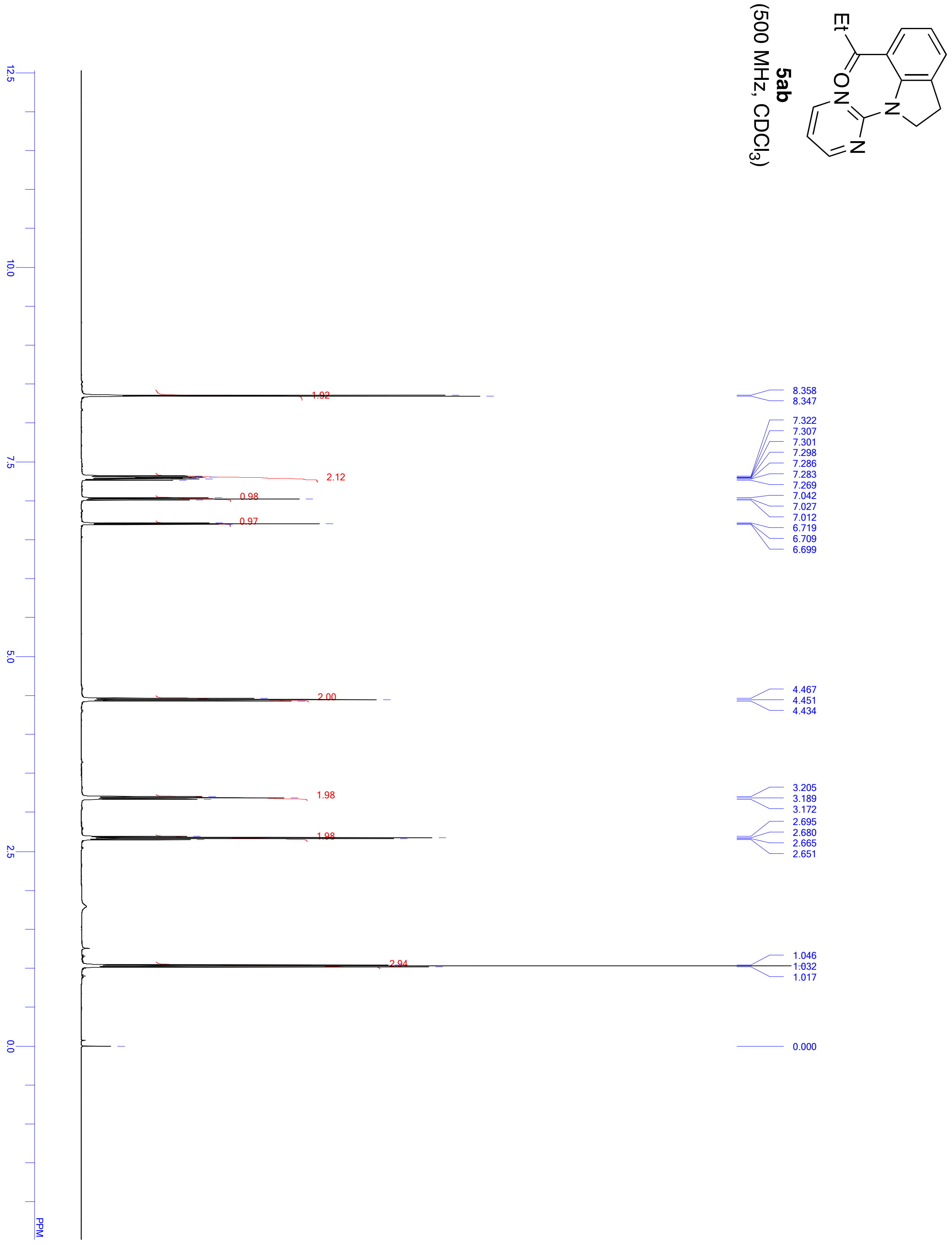
Et
O
N
N
N
5ab
(500 MHz, CDCl3)
8.358
8.347
7.322
7.307
7.301
7.298
7.286
7.283
7.269
7.042
7.027
7.012
6.719
6.709
6.699
4.467
4.451
4.434
3.205
3.189
3.172
2.695
2.680
2.665
2.651
1.046
1.032
1.017
0.000
1.92
2.12
0.98
0.97
2.00
1.98
1.98
2.94
12.5
10.0
7.5
5.0
2.5
0.0
PPM

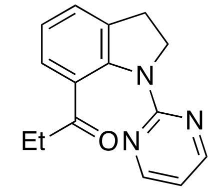

5ab
(125 MHz, $CDCl_3$)

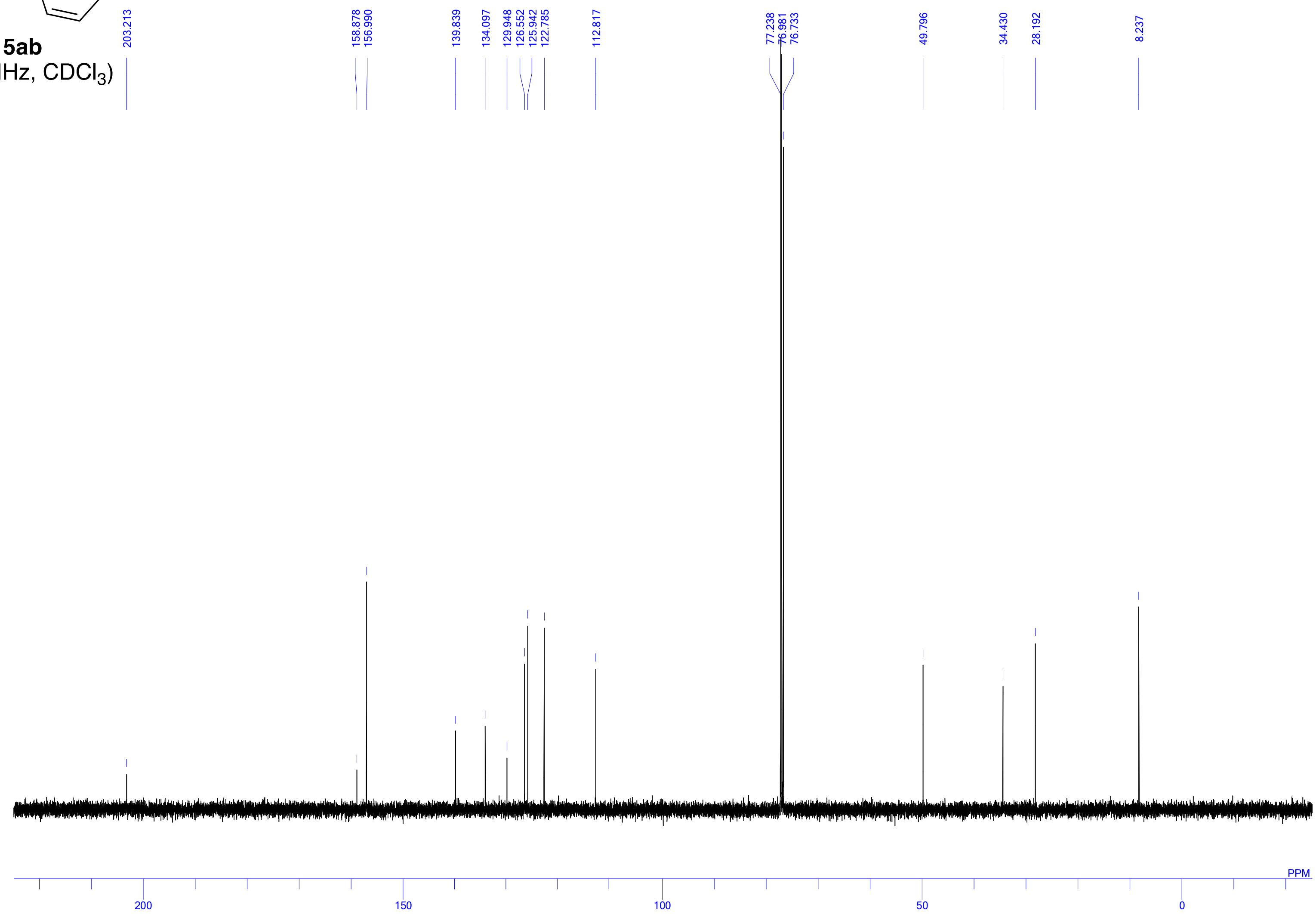

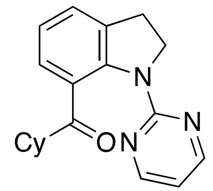

5ac
(500 MHz, $CDCl_3$)

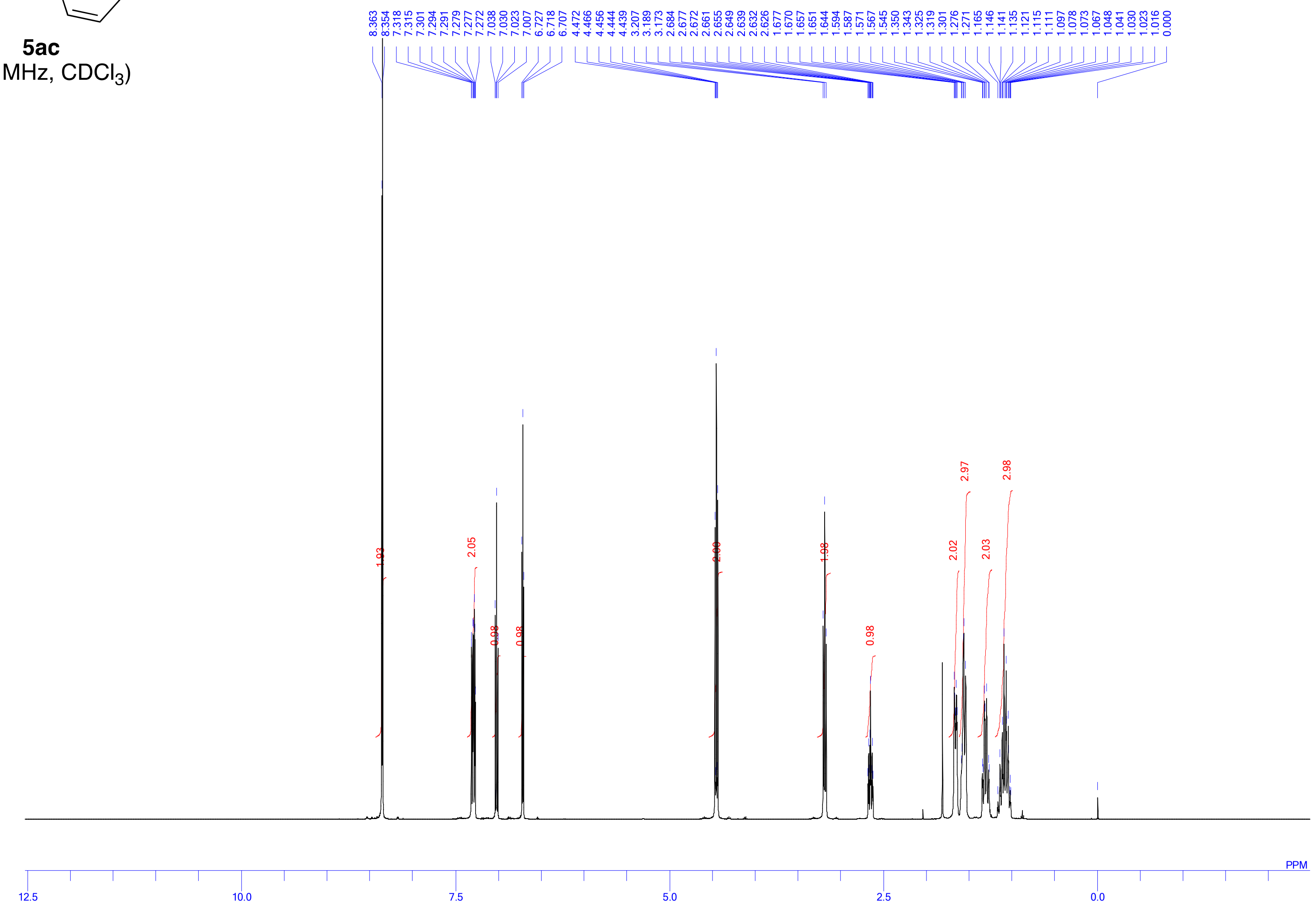

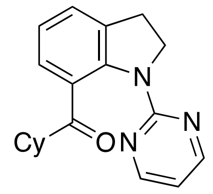

5ac
(125 MHz, $CDCl_3$)

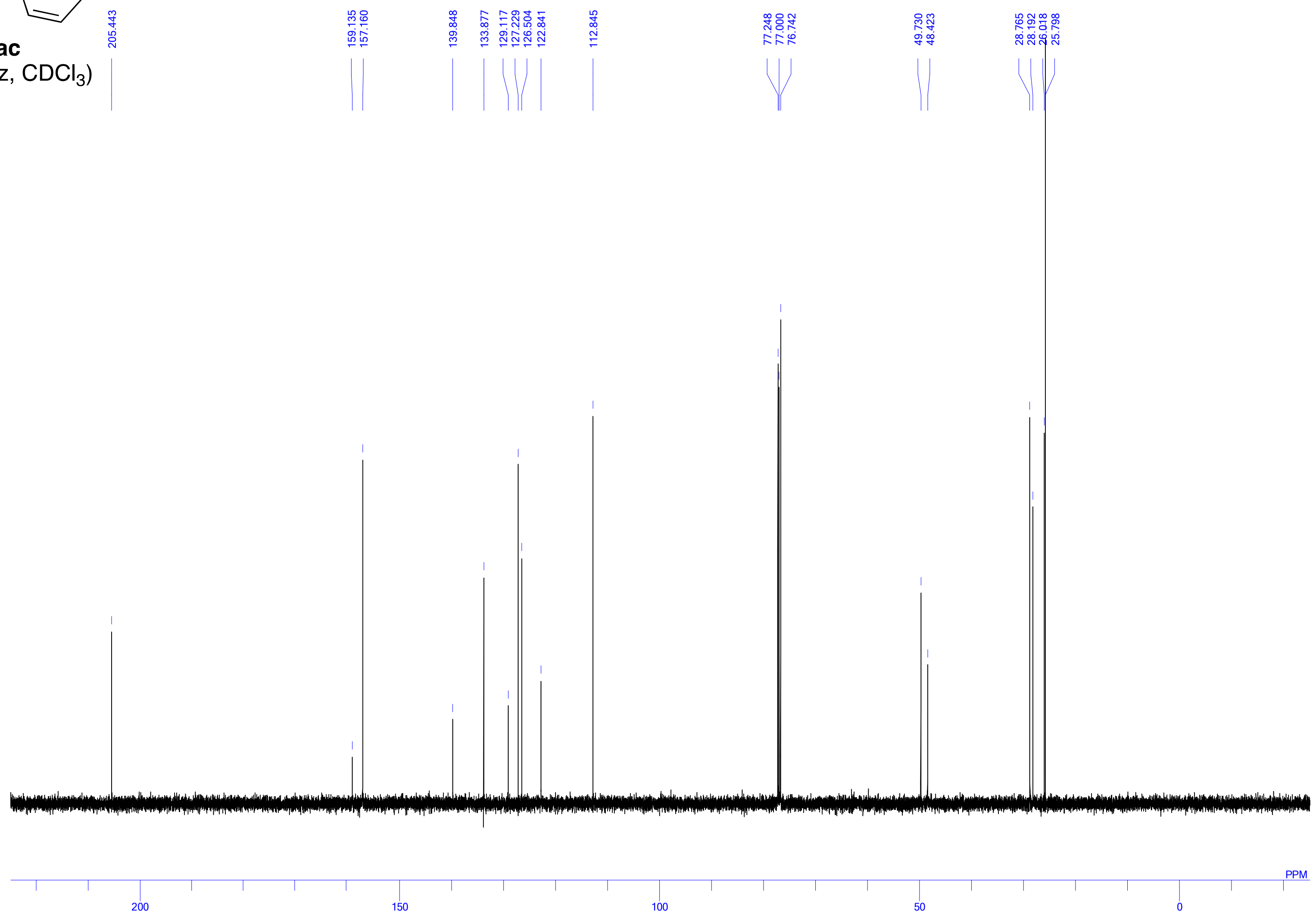

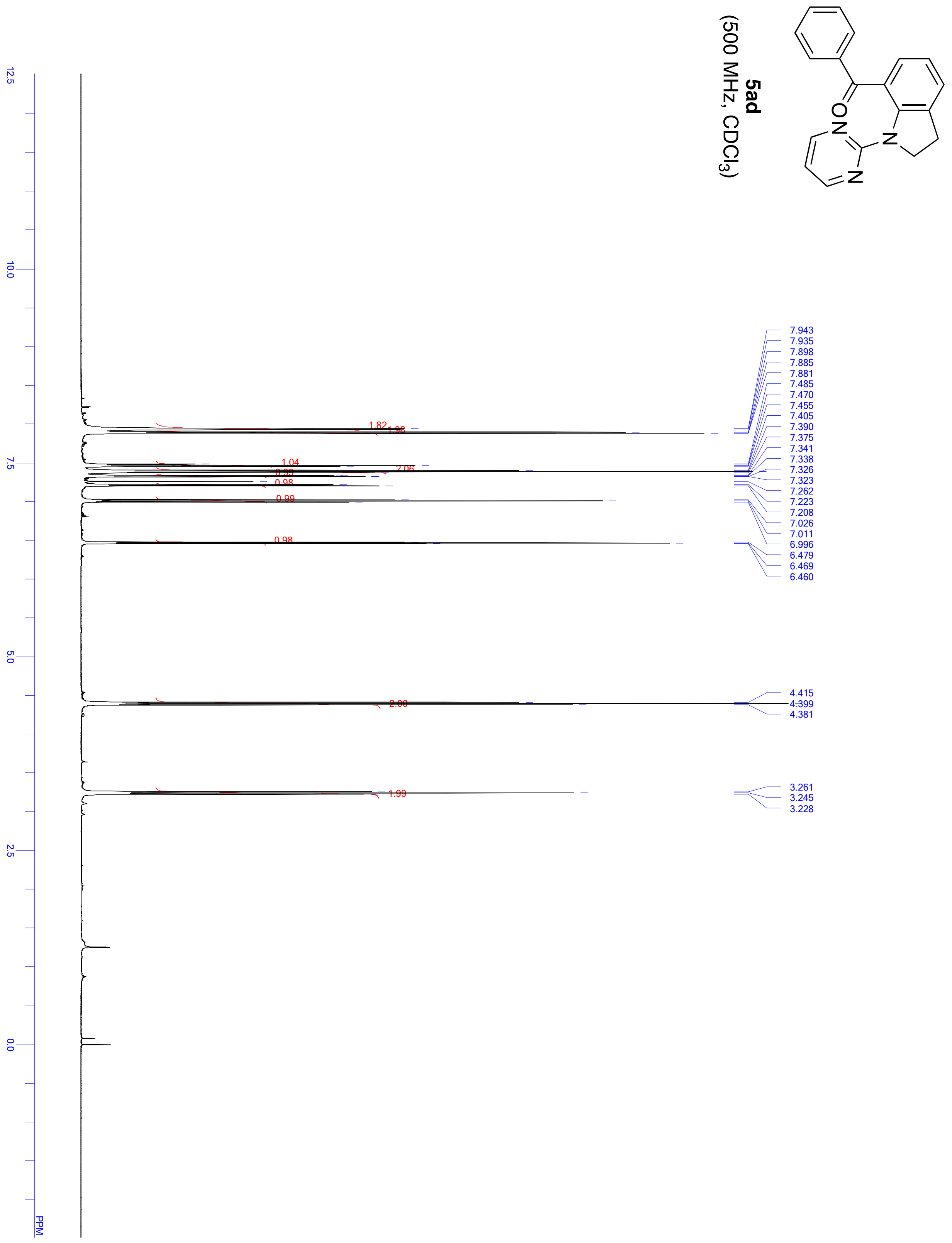
5ad
(500 MHz, CDCl$_3$)
7.943
7.935
7.898
7.885
7.881
7.485
7.470
7.455
7.405
7.390
7.375
7.341
7.338
7.326
7.323
7.262
7.223
7.208
7.026
7.011
6.996
6.479
6.469
6.460
4.415
4.399
4.381
3.261
3.245
3.228
1.82
1.96
1.04
2.06
0.98
0.99
0.98
2.00
1.99
12.5
10.0
7.5
5.0
2.5
0.0
PPM

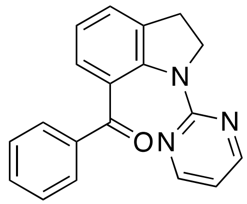

5ad
(125 MHz, $CDCl_3$)

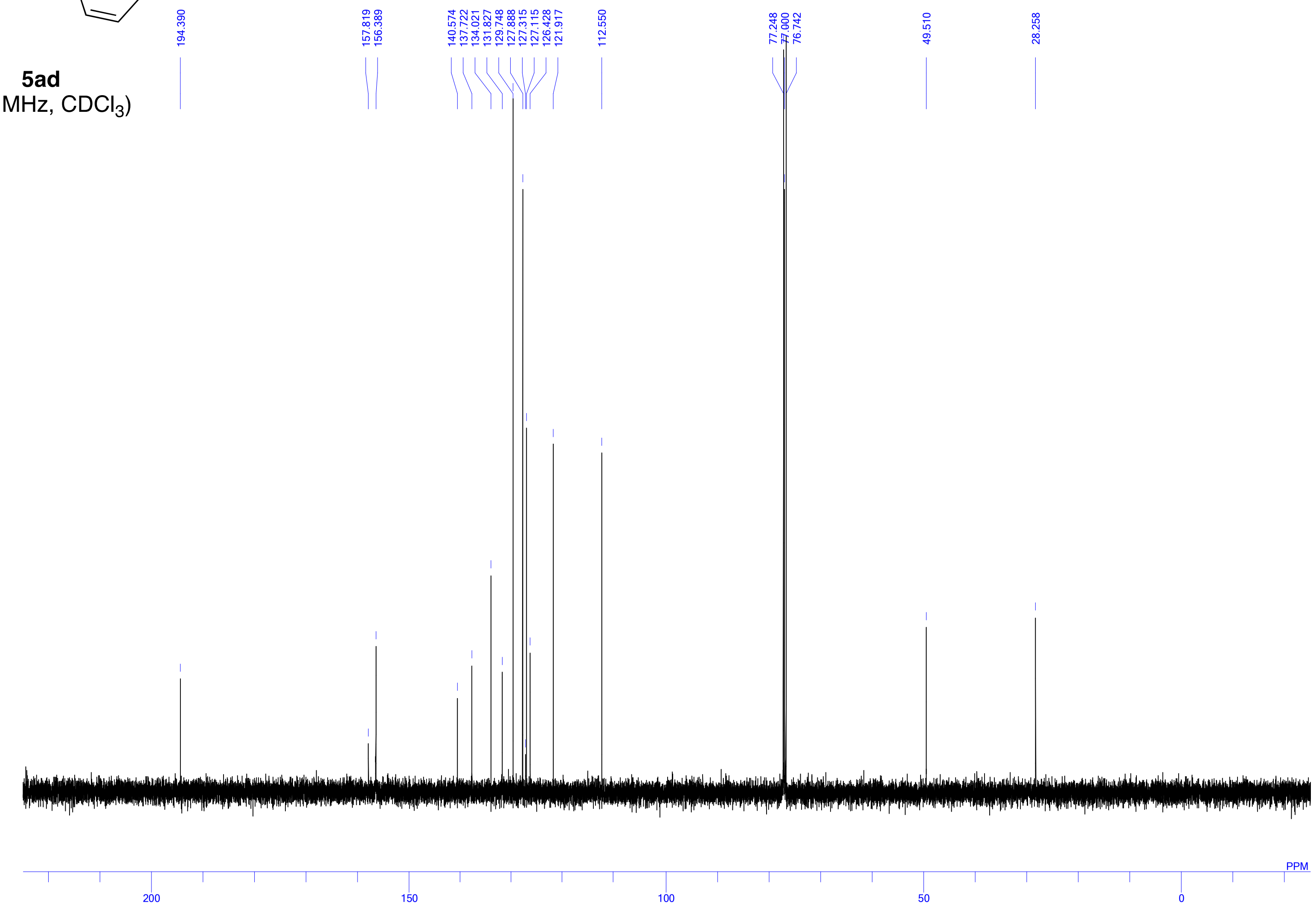

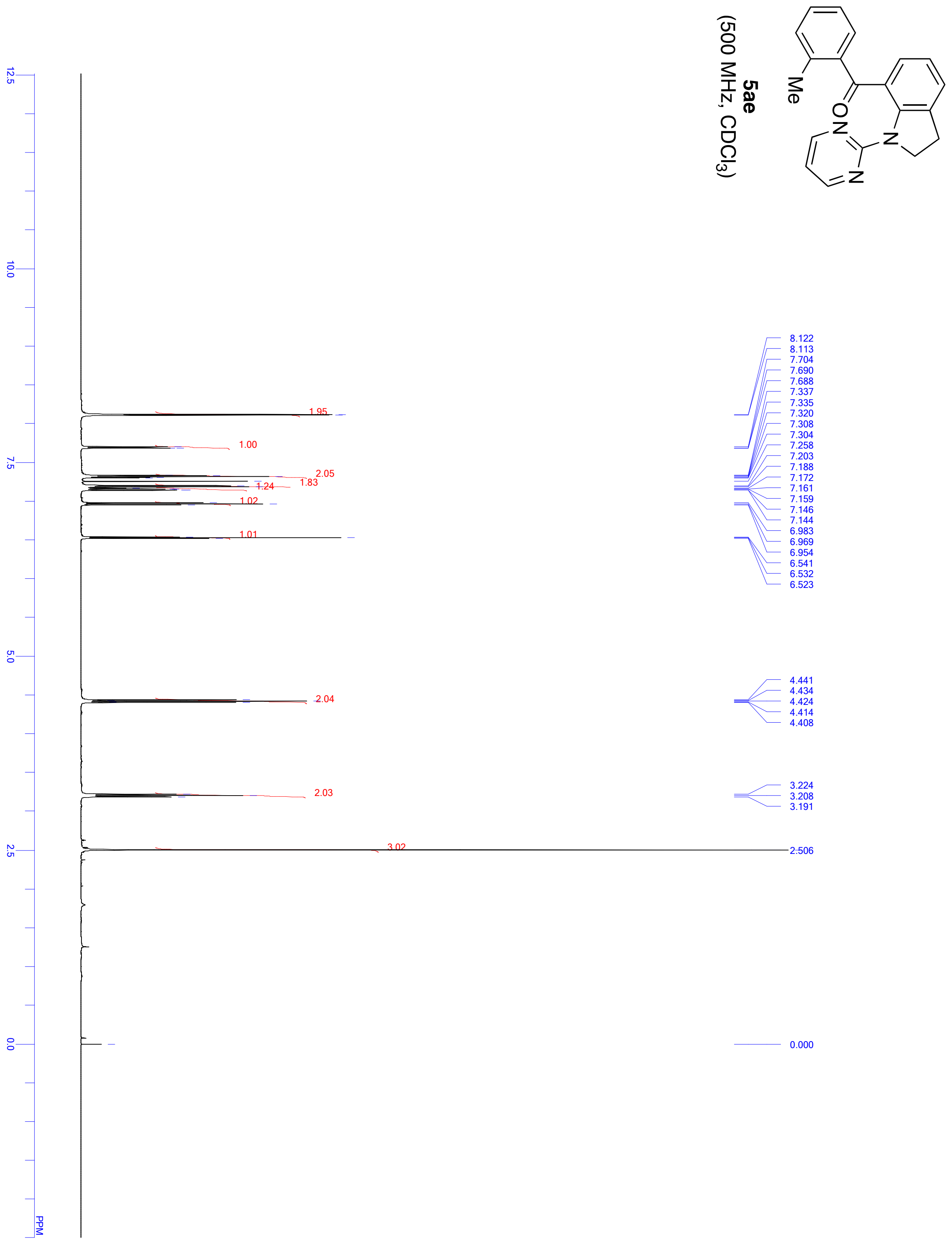
5ae
(500 MHz, CDCl3)
Me
O
N
N
N
8.122
8.113
7.704
7.690
7.688
7.337
7.335
7.320
7.308
7.304
7.258
7.203
7.188
7.172
7.161
7.159
7.146
7.144
6.983
6.969
6.954
6.541
6.532
6.523
4.441
4.434
4.424
4.414
4.408
3.224
3.208
3.191
2.506
0.000
1.95
1.00
2.05
1.83
1.24
1.02
1.01
2.04
2.03
3.02
12.5
10.0
7.5
5.0
2.5
0.0
PPM

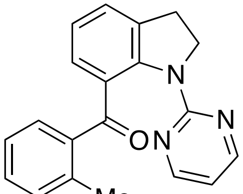

5ae
(125 MHz, $CDCl_3$)

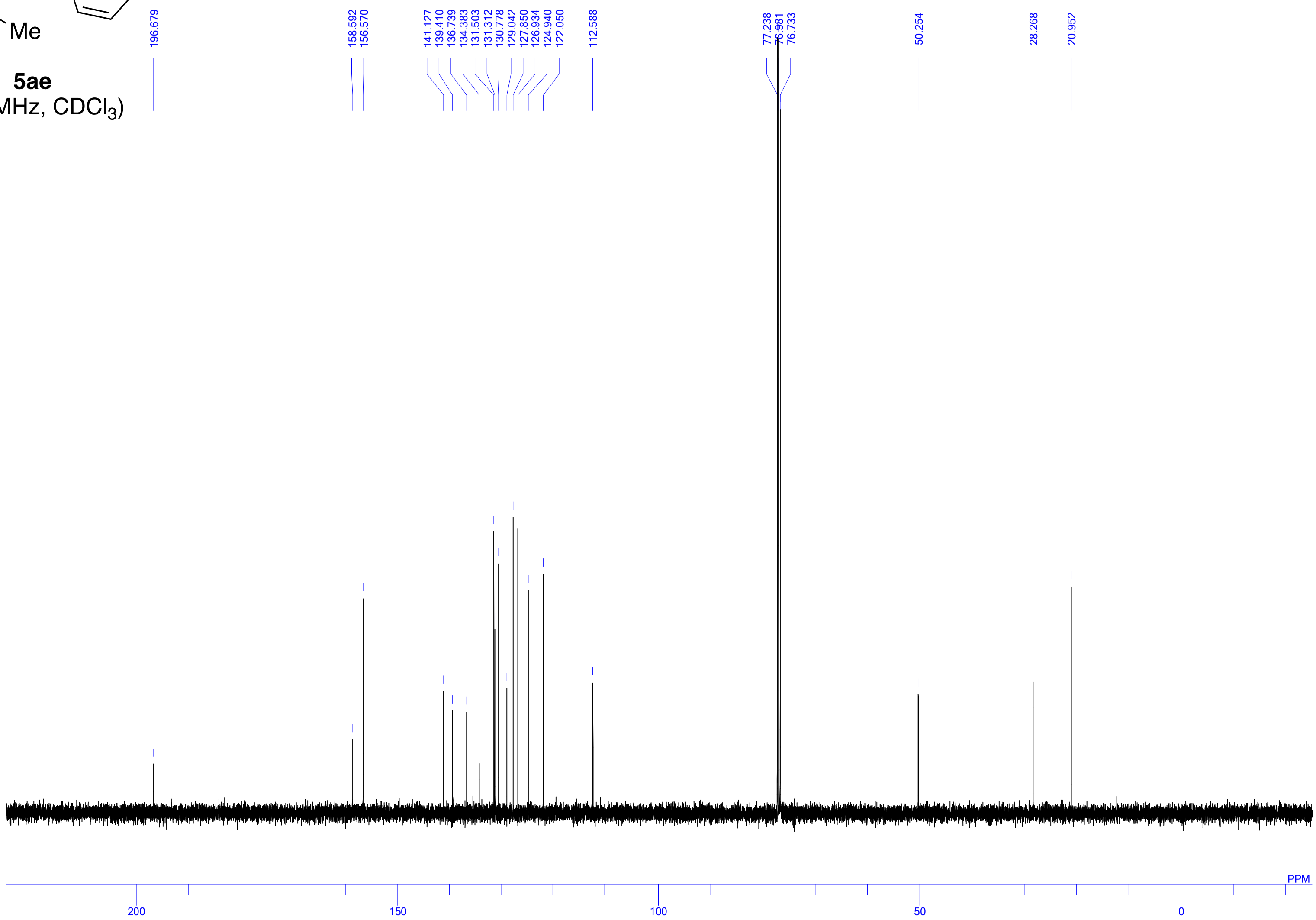

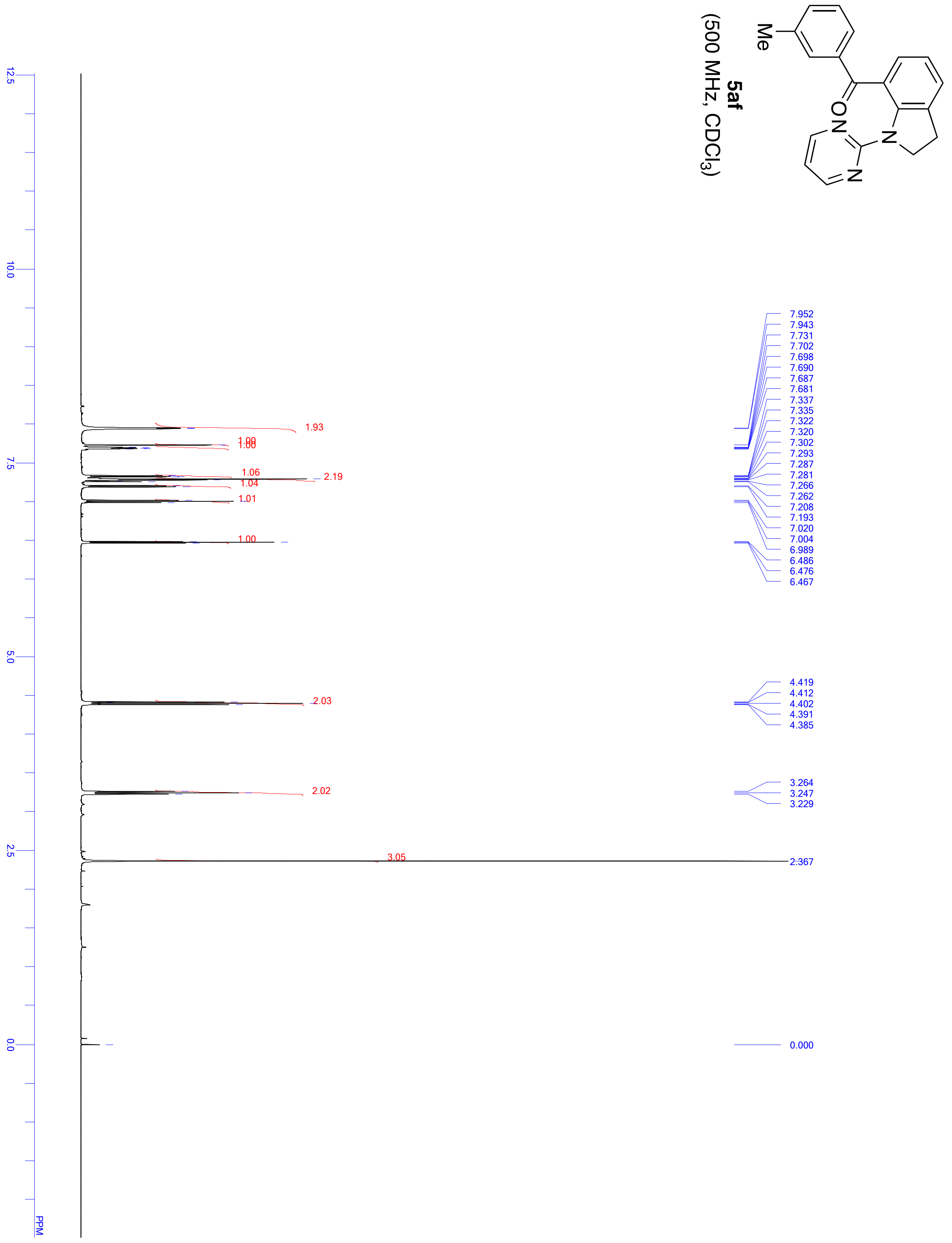

5af
(500 MHz, $CDCl_3$)
Me
O
N
N
N
7.952
7.943
7.731
7.702
7.698
7.690
7.687
7.681
7.337
7.335
7.322
7.320
7.302
7.293
7.287
7.281
7.266
7.262
7.208
7.193
7.020
7.004
6.989
6.486
6.476
6.467
4.419
4.412
4.402
4.391
4.385
3.264
3.247
3.229
2.367
0.000
1.93
1.00
1.00
1.06
2.19
1.04
1.01
1.00
2.03
2.02
3.05
12.5
10.0
7.5
5.0
2.5
0.0
PPM

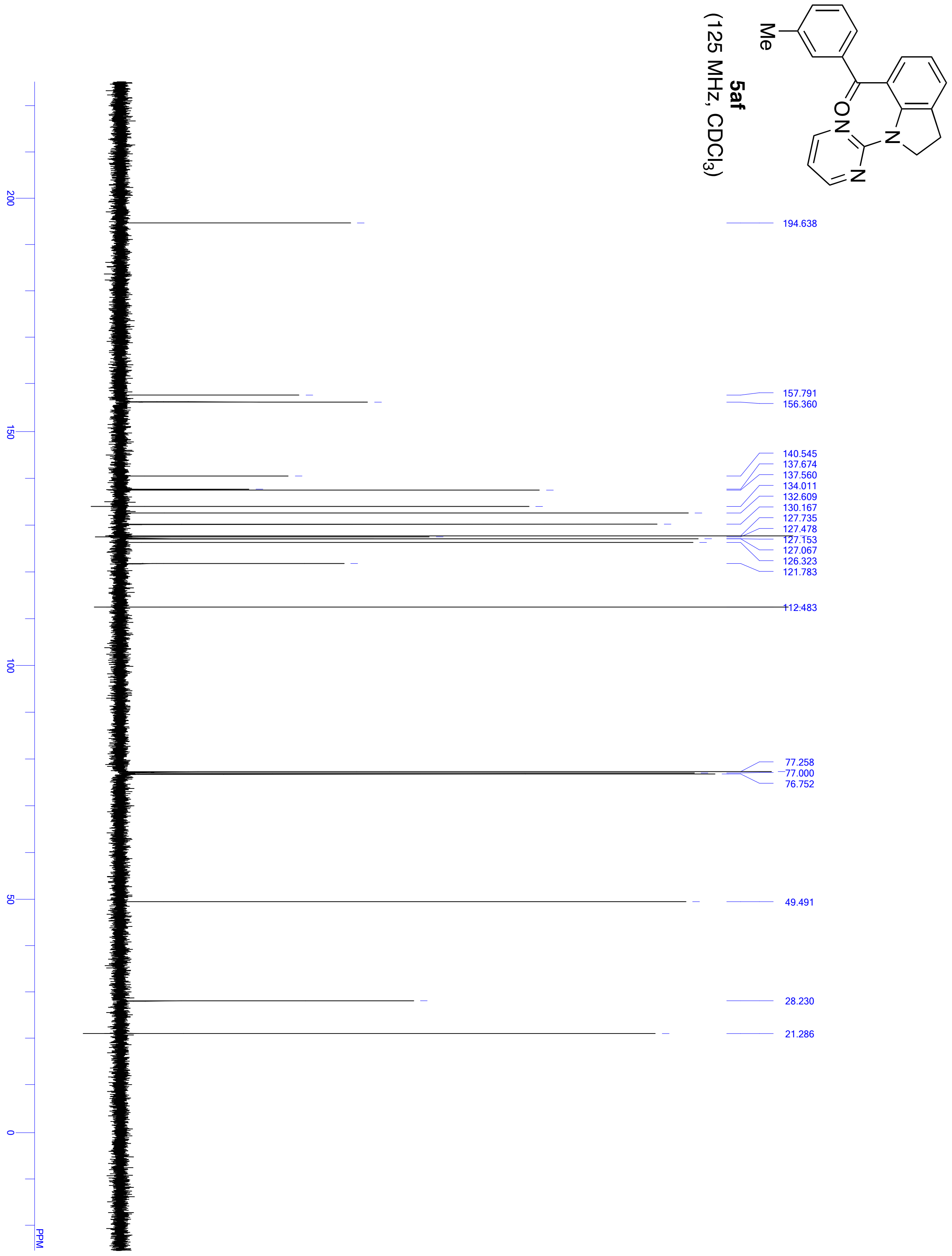
Me
O
N
N
N
5af
(125 MHz, CDCl3)
194.638
157.791
156.360
140.545
137.674
137.560
134.011
132.609
130.167
127.735
127.478
127.153
127.067
126.323
121.783
112.483
77.258
77.000
76.752
49.491
28.230
21.286
200
150
100
50
0
PPM

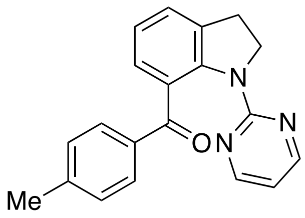

5ag
(500 MHz, $CDCl_3$)

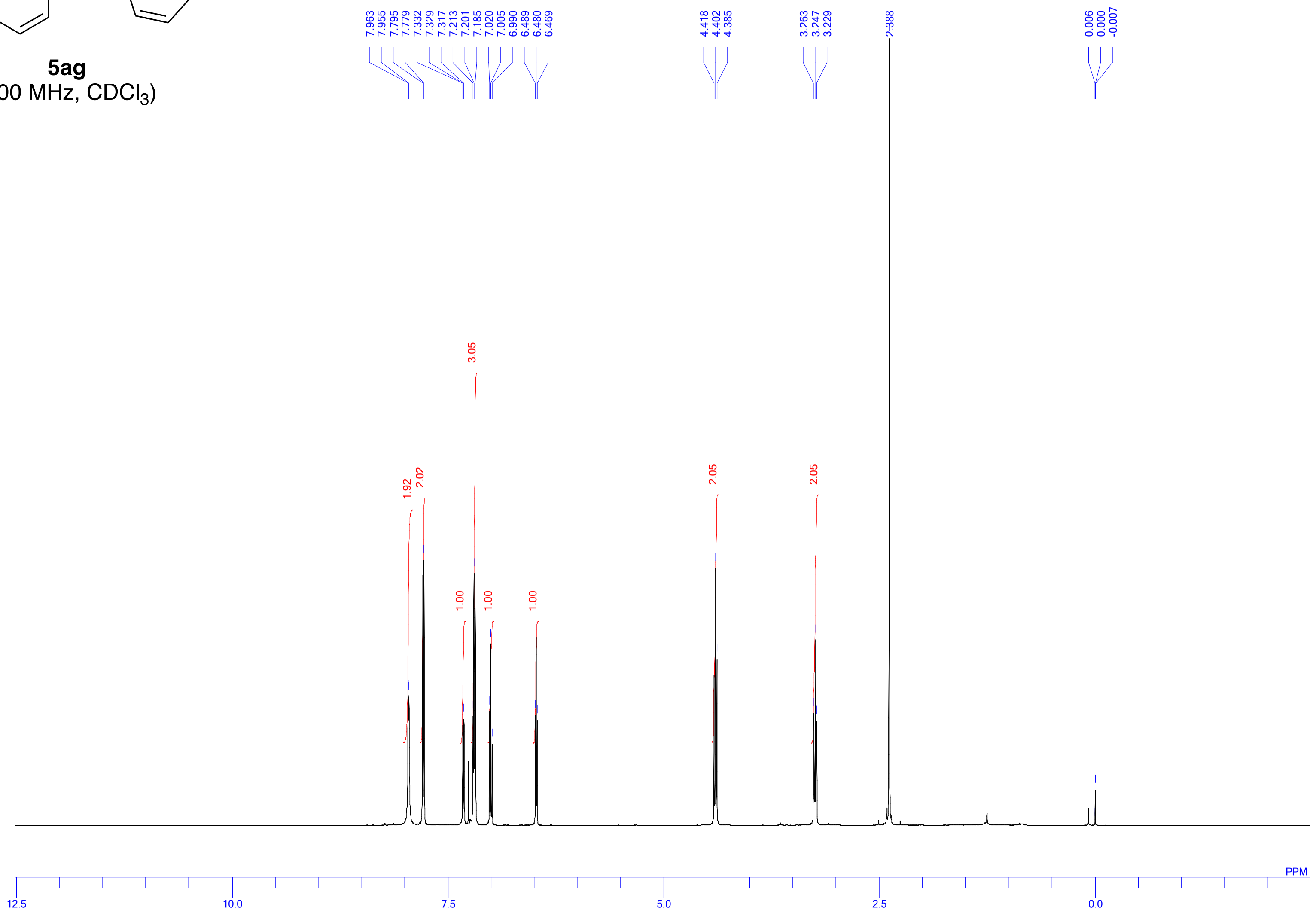

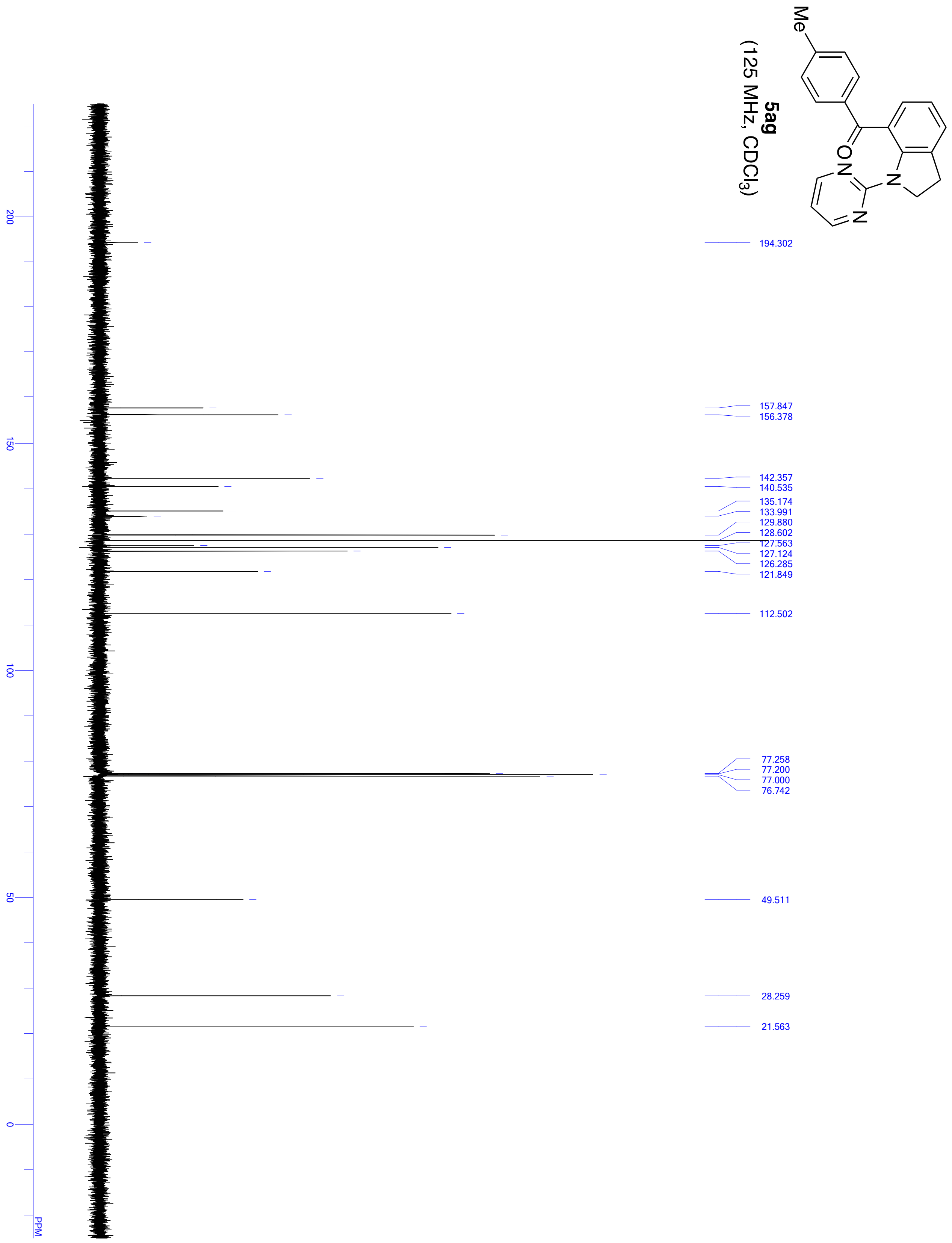
5ag
(125 MHz, CDCl3)
Me
O
N
N
N
194.302
157.847
156.378
142.357
140.535
135.174
133.991
129.880
128.602
127.563
127.124
126.285
121.849
112.502
77.258
77.200
77.000
76.742
49.511
28.259
21.563
200
150
100
50
0
PPM

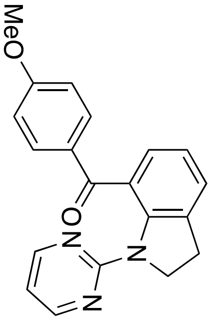

5ah
(500 MHz, $CDCl_3$)

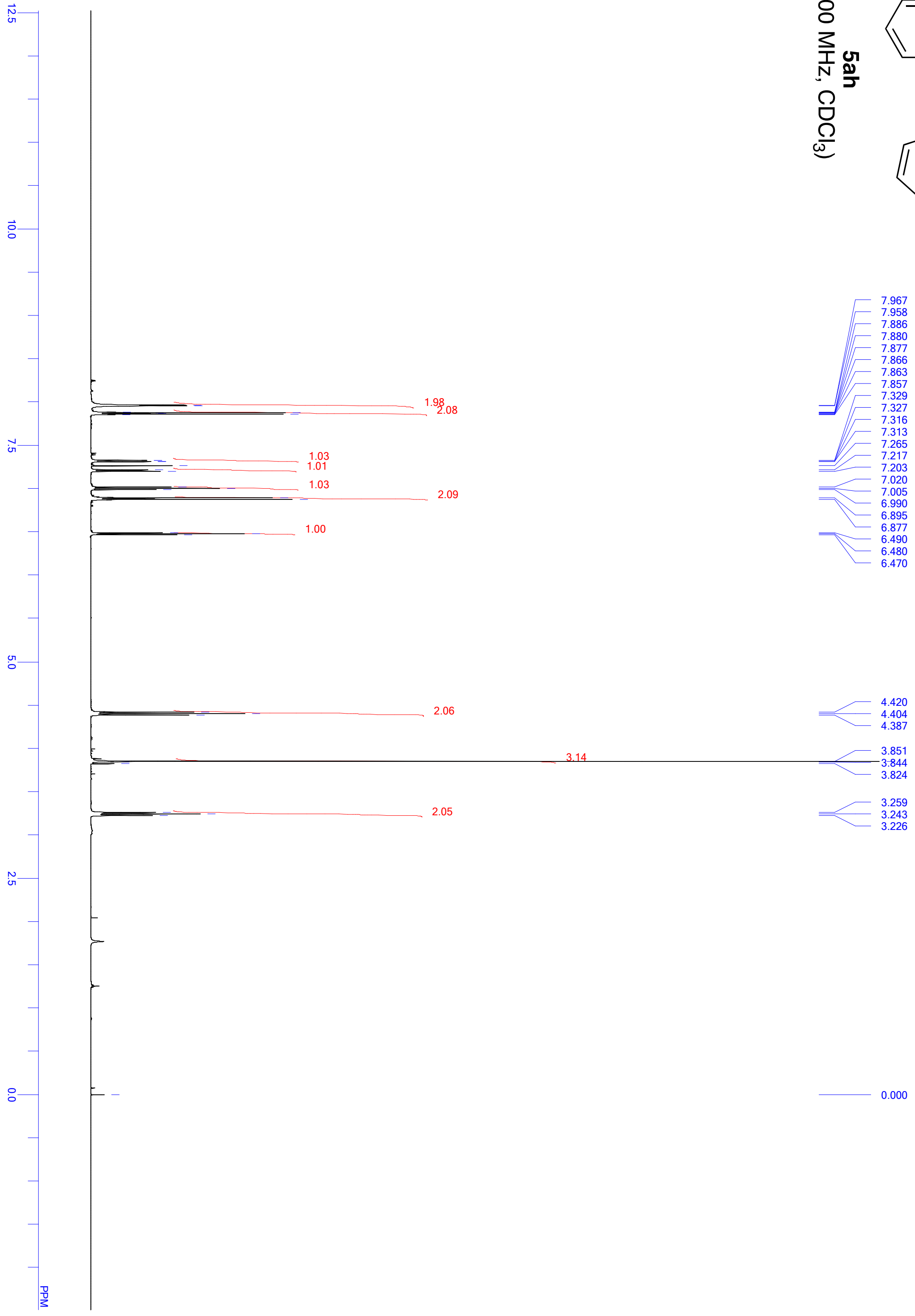

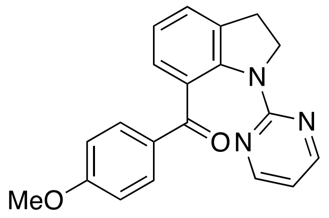

5ah
(125 MHz, $CDCl_3$)

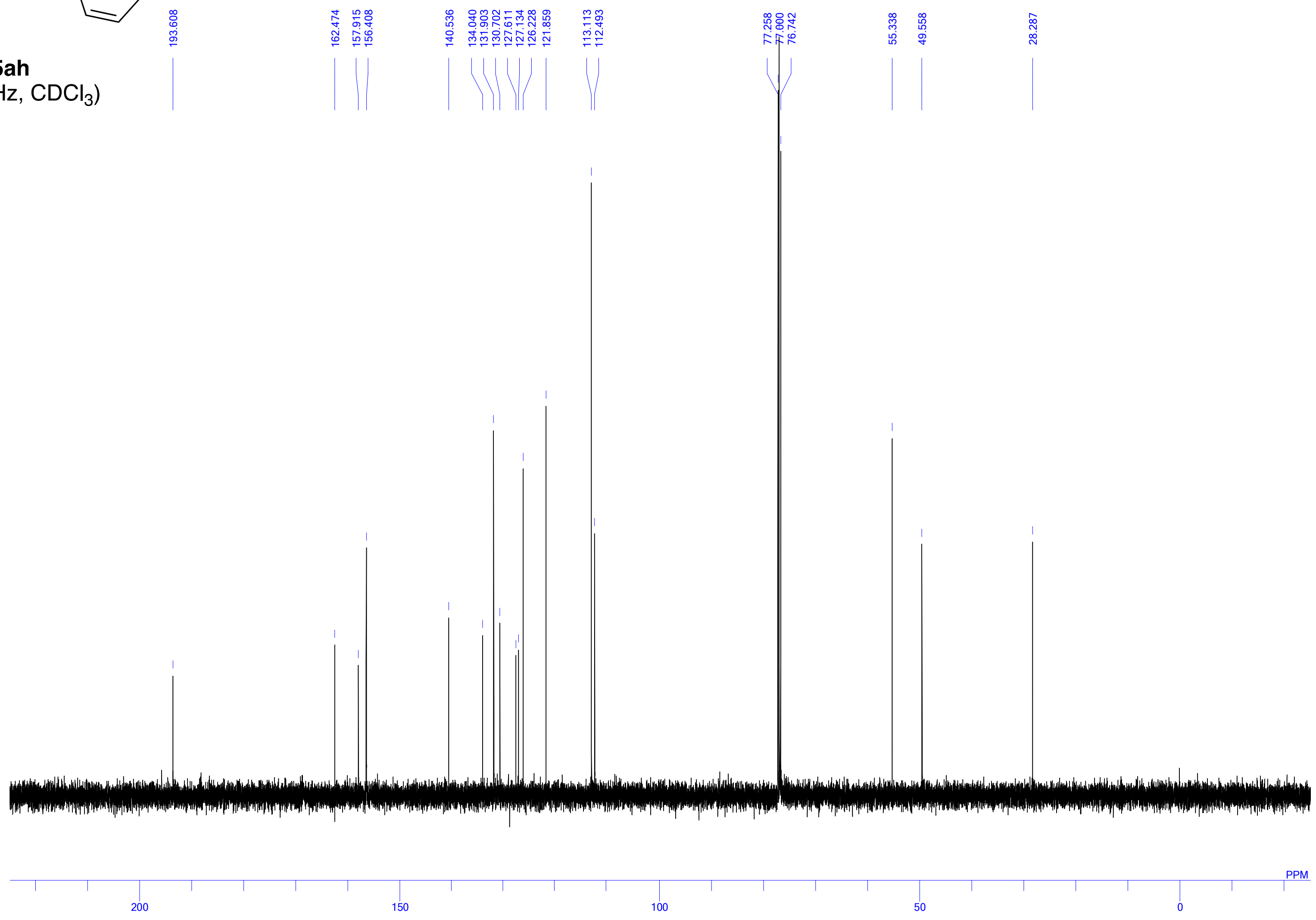

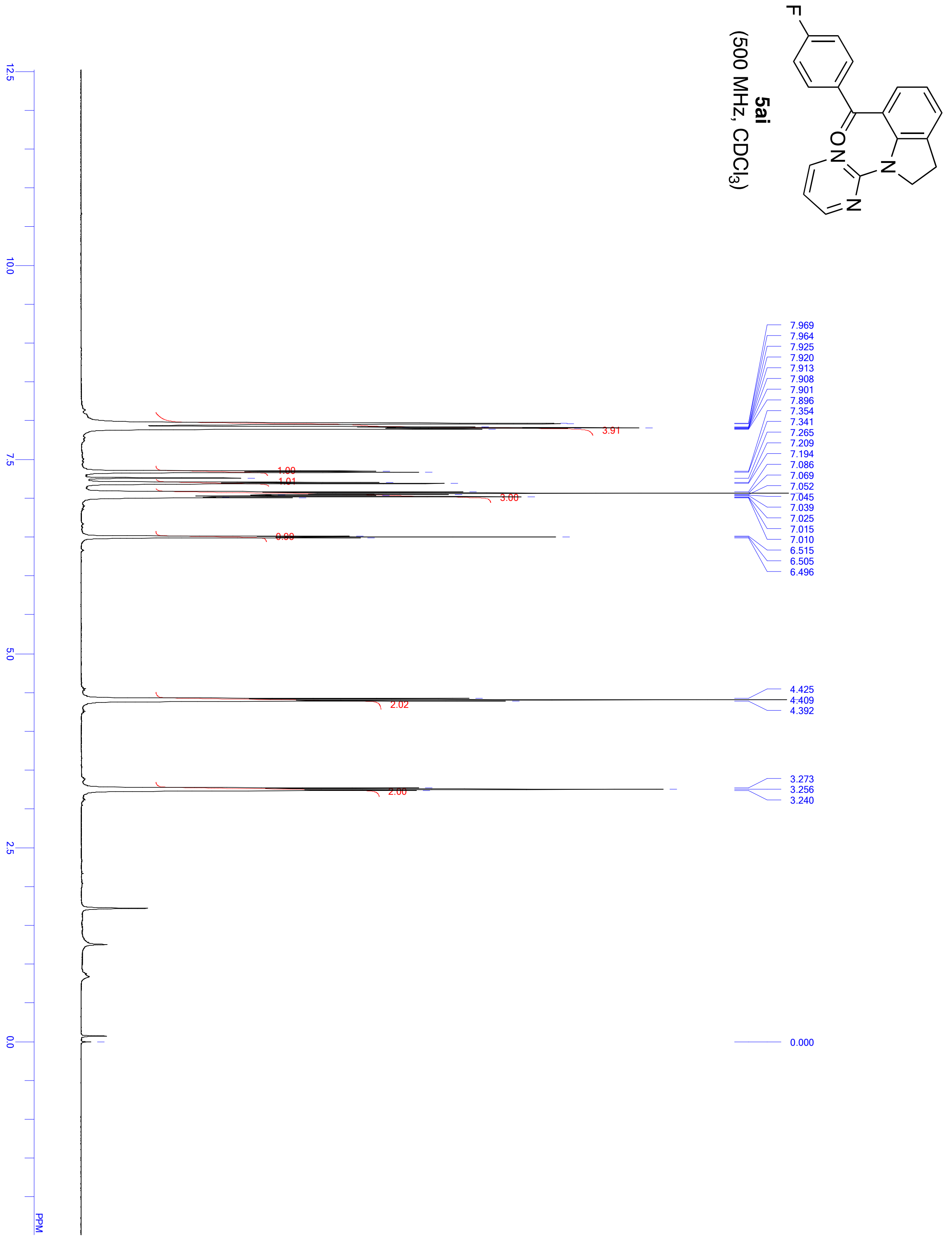

5ai
(500 MHz, CDCl3)
7.969
7.964
7.925
7.920
7.913
7.908
7.901
7.896
7.354
7.341
7.265
7.209
7.194
7.086
7.069
7.052
7.045
7.039
7.025
7.015
7.010
6.515
6.505
6.496
4.425
4.409
4.392
3.273
3.256
3.240
0.000
3.91
1.00
1.01
3.00
0.99
2.02
2.00
12.5
10.0
7.5
5.0
2.5
0.0
PPM

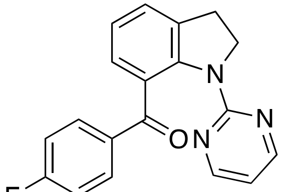

5ai
(125 MHz, $CDCl_3$)

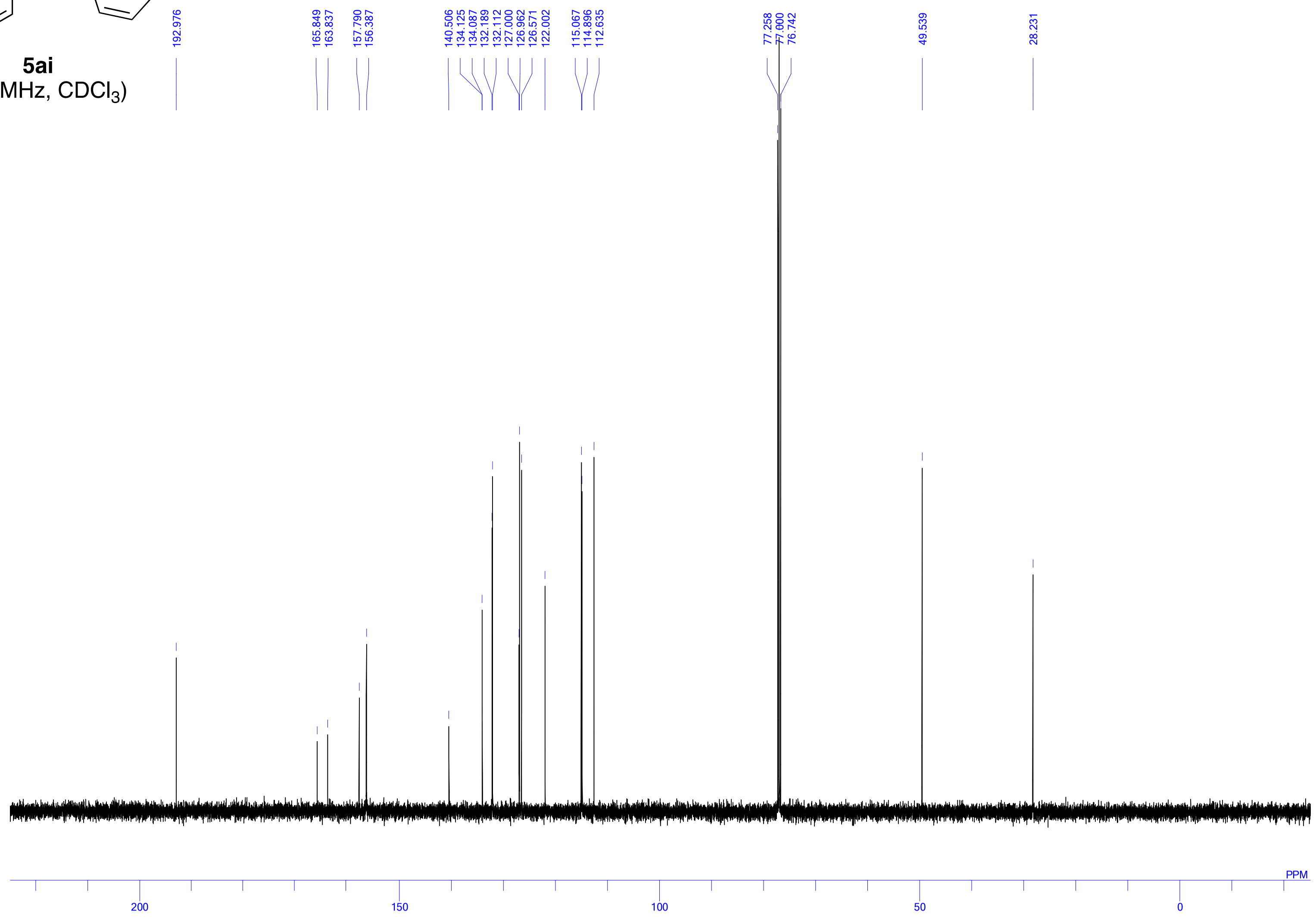

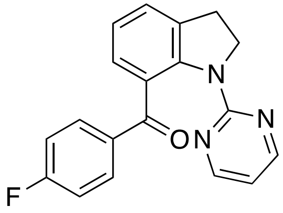

5ai
(470 MHz, $CDCl_3$)

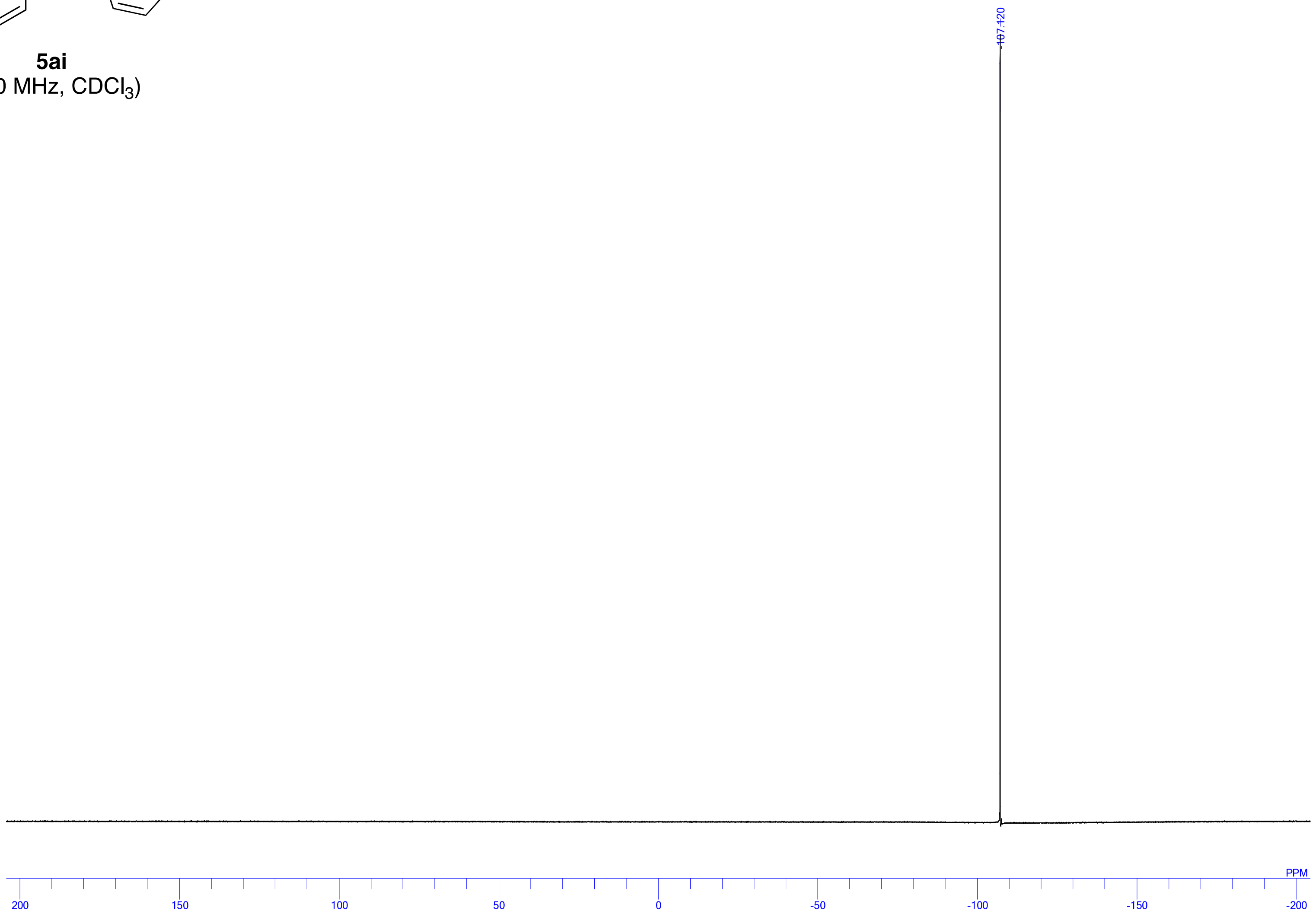

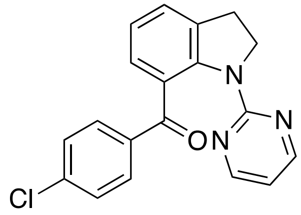

5aj
(500 MHz, $CDCl_3$)

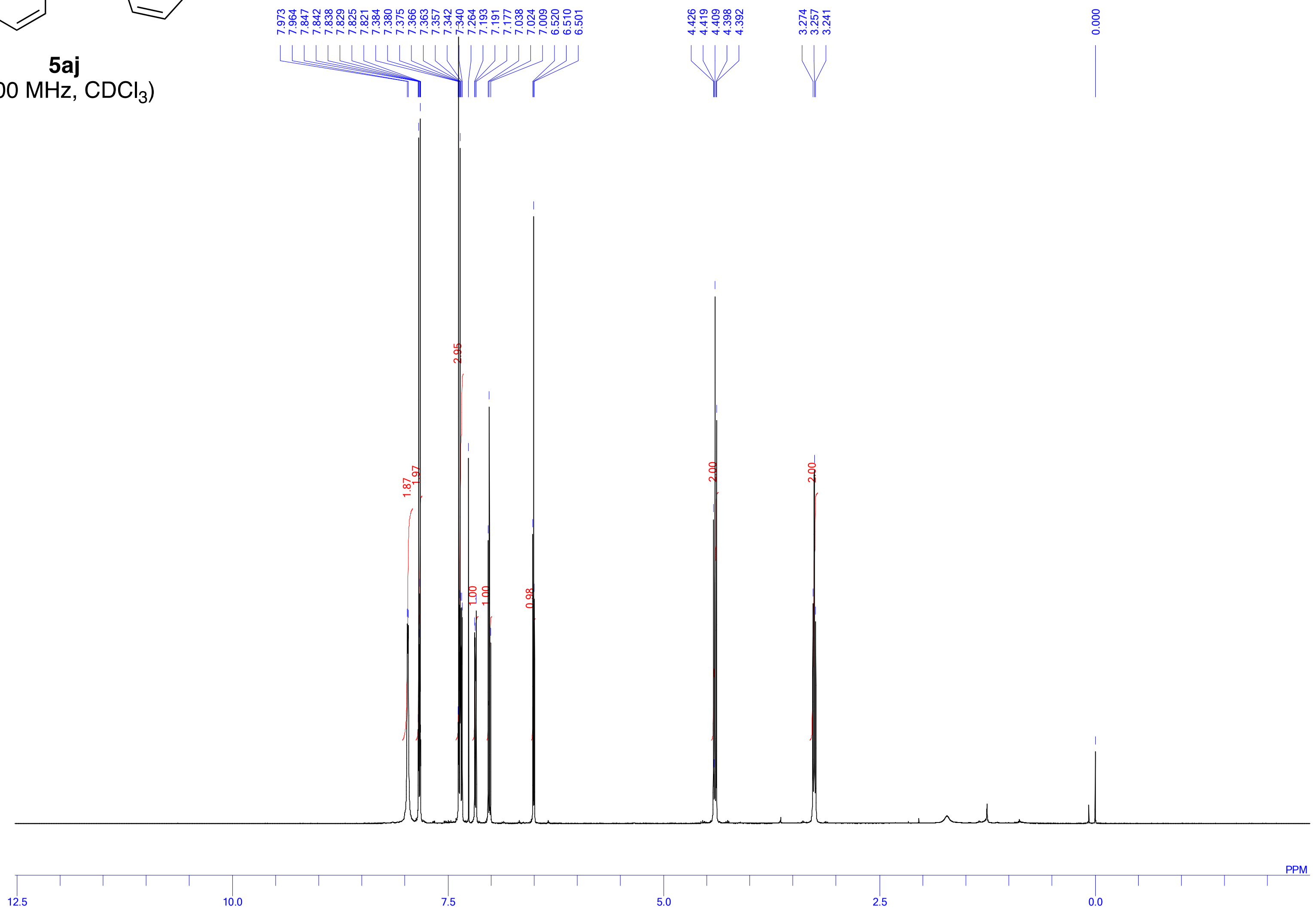

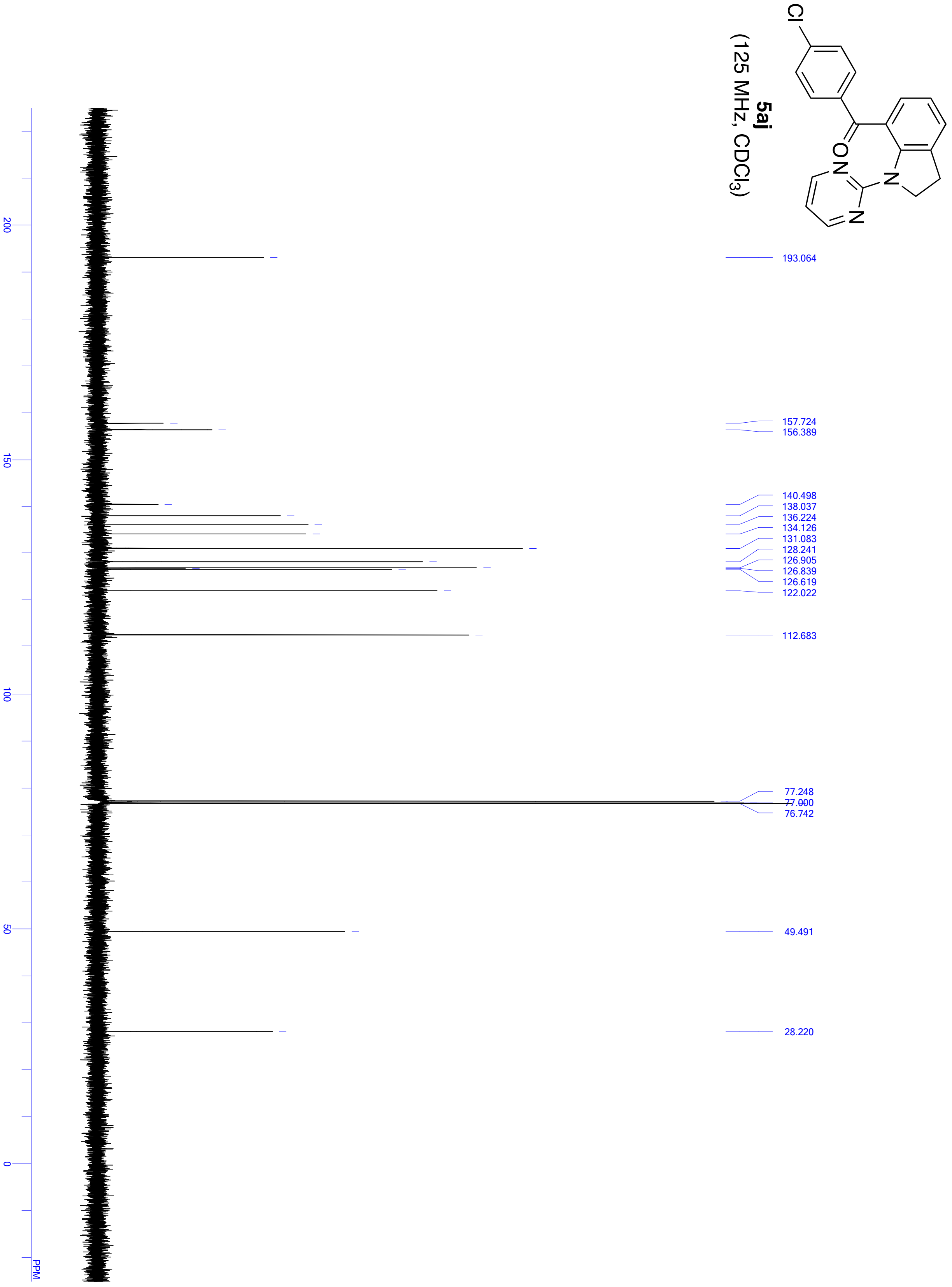

Cl
O
N
N
N
5aj
(125 MHz, CDCl3)
193.064
157.724
156.389
140.498
138.037
136.224
134.126
131.083
128.241
126.905
126.839
126.619
122.022
112.683
77.248
77.000
76.742
49.491
28.220
200
150
100
50
0
PPM

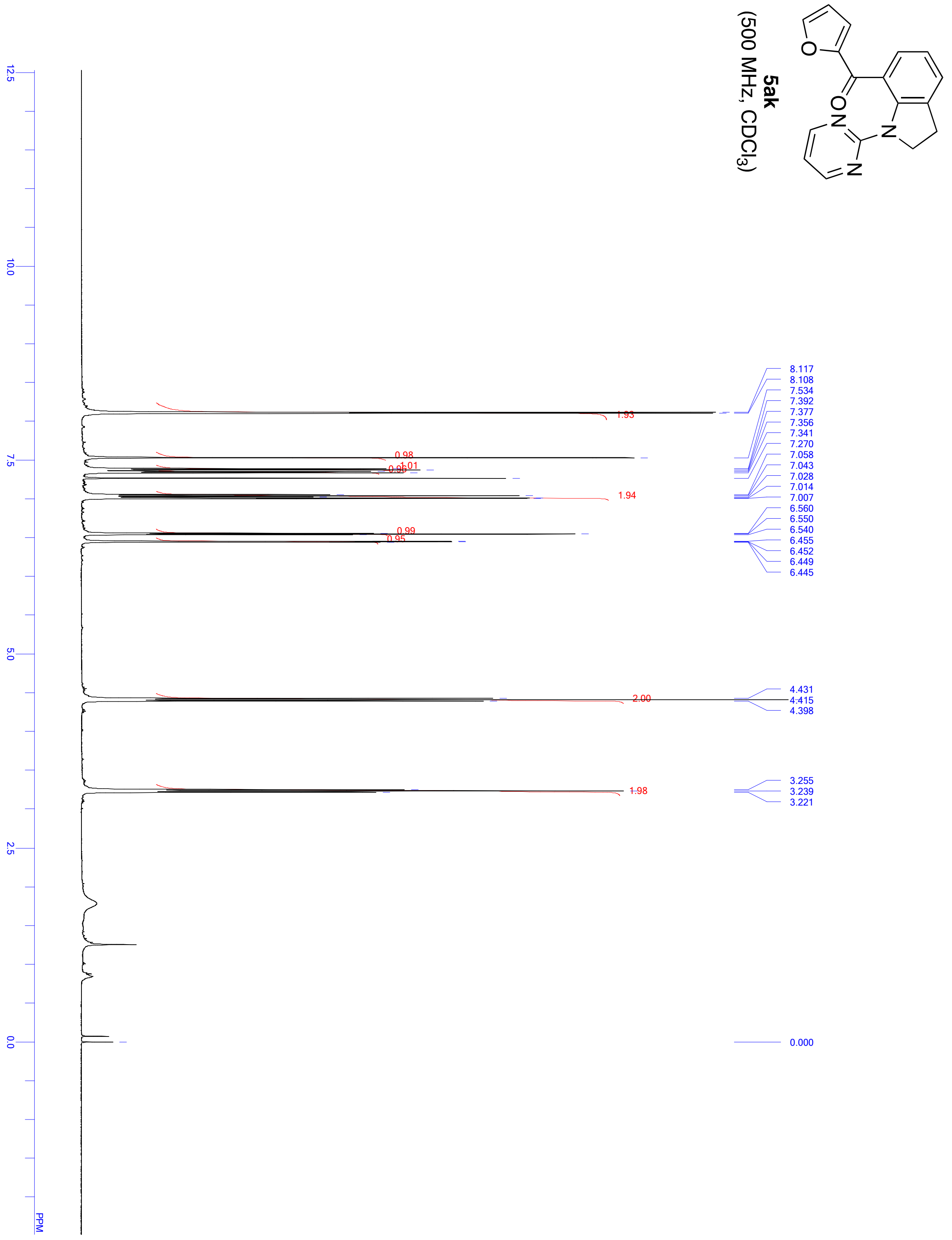
5ak
(500 MHz, CDCl3)
8.117
8.108
7.534
7.392
7.377
7.356
7.341
7.270
7.058
7.043
7.028
7.014
7.007
6.560
6.550
6.540
6.455
6.452
6.449
6.445
4.431
4.415
4.398
3.255
3.239
3.221
0.000
1.93
0.98
1.01
0.99
1.94
0.99
0.95
2.00
1.98
12.5
10.0
7.5
5.0
2.5
0.0
PPM

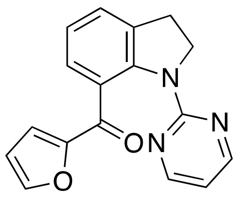

5ak
(125 MHz, $CDCl_3$)

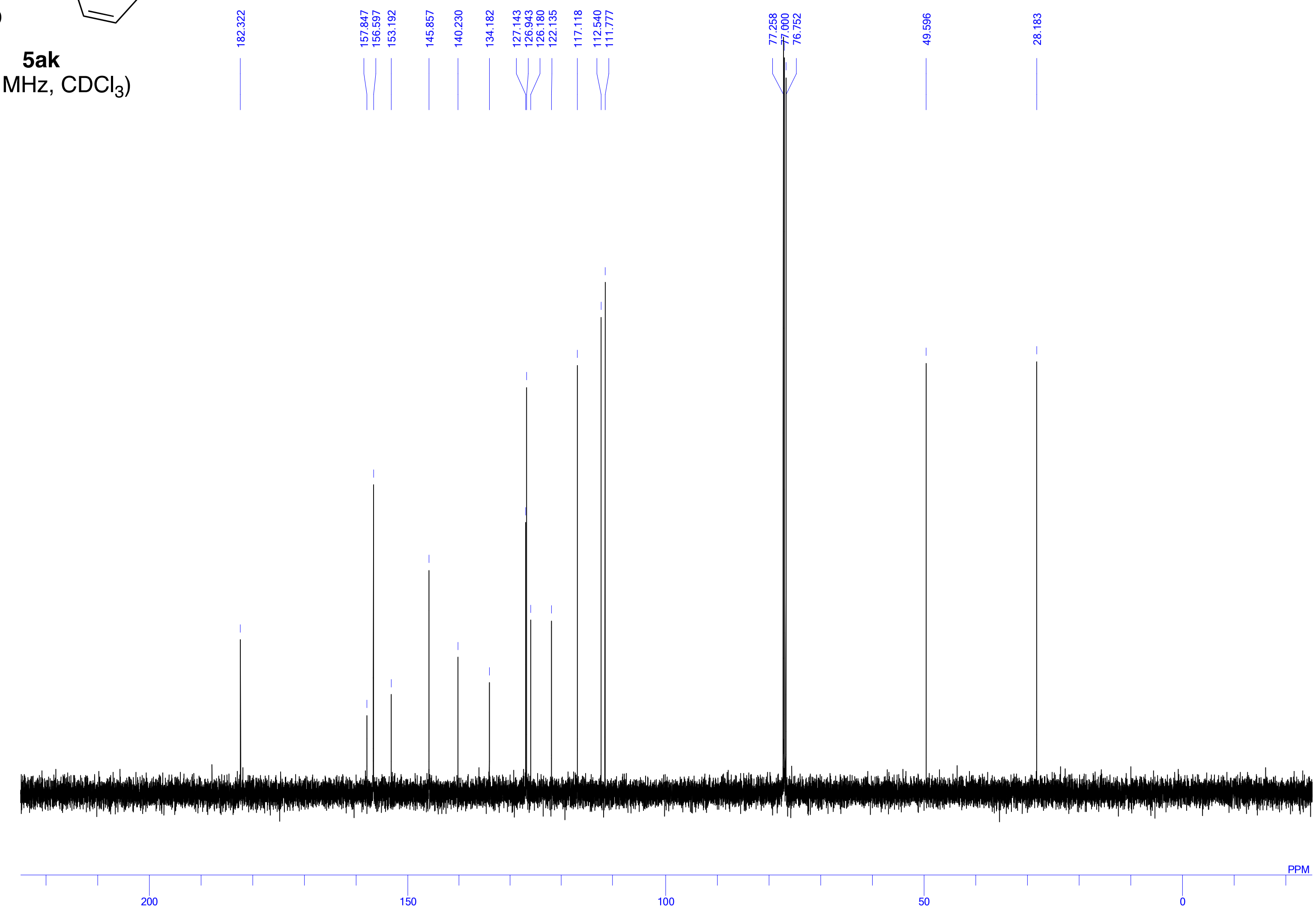

Supplement: Supplementary file 1 — ol1c04195_si_001.pdf [file ol1c04195_si_001.pdf]
